# Virtual Patient Simulations in Health Professions Education: Systematic Review and Meta-Analysis by the Digital Health Education Collaboration

**DOI:** 10.2196/14676

**Published:** 2019-07-02

**Authors:** Andrzej A Kononowicz, Luke A Woodham, Samuel Edelbring, Natalia Stathakarou, David Davies, Nakul Saxena, Lorainne Tudor Car, Jan Carlstedt-Duke, Josip Car, Nabil Zary

**Affiliations:** 1 Department of Bioinformatics and Telemedicine, Jagiellonian University Medical College Kraków Poland; 2 Institute of Medical and Biomedical Education, St George’s, University of London London United Kingdom; 3 Department of Learning, Informatics, Management and Ethics, Karolinska Institutet Stockholm Sweden; 4 Department of Medical and Health Sciences Linköping University Linköping Sweden; 5 Learning and Professional Development Group School of Health Sciences Örebro University Örebro Sweden; 6 Warwick Medical School, University of Warwick Coventry United Kingdom; 7 Health Services and Outcomes Research, National Healthcare Group Singapore Singapore; 8 Family Medicine and Primary Care, Lee Kong Chian School of Medicine, Nanyang Technological University Singapore Singapore; 9 Department of Primary Care and Public Health, School of Public Health, Imperial College London London United Kingdom; 10 President’s Office, Nanyang Technological University Singapore Singapore; 11 Centre for Population Health Sciences Lee Kong Chian School of Medicine Nanyang Technological University Singapore Singapore; 12 Global eHealth Unit, Department of Primary Care and Public Health, School of Public Health, Imperial College London London United Kingdom; 13 Games for Health Innovations Centre Lee Kong Chian School of Medicine Nanyang Technological University Singapore Singapore; 14 Mohammed Bin Rashid University of Medicine and Health Sciences Dubai United Arab Emirates

**Keywords:** computer simulation, professional education, computer-assisted instruction, systematic review, meta-analysis

## Abstract

**Background:**

Virtual patients are interactive digital simulations of clinical scenarios for the purpose of health professions education. There is no current collated evidence on the effectiveness of this form of education.

**Objective:**

The goal of this study was to evaluate the effectiveness of virtual patients compared with traditional education, blended with traditional education, compared with other types of digital education, and design variants of virtual patients in health professions education. The outcomes of interest were knowledge, skills, attitudes, and satisfaction.

**Methods:**

We performed a systematic review on the effectiveness of virtual patient simulations in pre- and postregistration health professions education following Cochrane methodology. We searched 7 databases from the year 1990 up to September 2018. No language restrictions were applied. We included randomized controlled trials and cluster randomized trials. We independently selected studies, extracted data, and assessed risk of bias and then compared the information in pairs. We contacted study authors for additional information if necessary. All pooled analyses were based on random-effects models.

**Results:**

A total of 51 trials involving 4696 participants met our inclusion criteria. Furthermore, 25 studies compared virtual patients with traditional education, 11 studies investigated virtual patients as blended learning, 5 studies compared virtual patients with different forms of digital education, and 10 studies compared different design variants. The pooled analysis of studies comparing the effect of virtual patients to traditional education showed similar results for knowledge (standardized mean difference [SMD]=0.11, 95% CI −0.17 to 0.39, I^2^=74%, n=927) and favored virtual patients for skills (SMD=0.90, 95% CI 0.49 to 1.32, I^2^=88%, n=897). Studies measuring attitudes and satisfaction predominantly used surveys with item-by-item comparison. Trials comparing virtual patients with different forms of digital education and design variants were not numerous enough to give clear recommendations. Several methodological limitations in the included studies and heterogeneity contributed to a generally low quality of evidence.

**Conclusions:**

Low to modest and mixed evidence suggests that when compared with traditional education, virtual patients can more effectively improve skills, and at least as effectively improve knowledge. The skills that improved were clinical reasoning, procedural skills, and a mix of procedural and team skills. We found evidence of effectiveness in both high-income and low- and middle-income countries, demonstrating the global applicability of virtual patients. Further research should explore the utility of different design variants of virtual patients.

## Introduction

### Background

Health care education is confronted with many global challenges. Shorter hospital stays, specialization of care, higher patient safety measures, and shortage of clinical teachers all diminish the traditional opportunities for the training of health professions through direct patient contact [1,2]. Early health professions education is often dominated by theoretical presentations with insufficient connection to clinical practice [3]. The need to increase numbers and quality of the health workforce is especially visible in low-and-middle-income countries, where the need to scale up high-quality health education and introduce educational innovations is most pressing [4]. Therefore, the global medical education community is perpetually searching for methods that can be applied to improve the relevance, increase the spread, and accelerate the educational process for health professions [5].

Digital education (often referred to as e-learning) is “the act of teaching and learning by means of digital technologies” [6]. It encompasses a multitude of educational concepts, approaches, methods, and technologies. Digital health education comprises, for example, offline learning, mobile learning, serious games, or virtual reality environments. We have conducted this systematic review as part of a review series on digital health education [6-19] and focused it on the simulation modality called *virtual patients*.

Virtual patients are defined as interactive computer simulations of real-life clinical scenarios for the purpose of health professions training, education, or assessment [20]. This broad definition encompasses a variety of systems that use different technologies and address various learning needs [21]. The learner is cast into the role of a health care provider who makes decisions about the type and order of clinical information acquired, differential diagnosis, and management and follow-up of the patient. Virtual patients are hypothesized to primarily address learning needs in clinical reasoning [22,23]. However, an influence of the use of virtual patients on other educational outcomes has been reported in previous literature [21,24].

The educational use of virtual patients may be understood through experiential learning theory [25,26]. Following this theoretical model of action and reflection, virtual patients expose learners to simulated clinical experiences, providing mechanisms for information gathering and clinical decision making in a safe environment [27]. Exposing the learner to many simulated clinical scenarios supports learning diagnostic processes [28] while acquainting learners with a standardized set of clinical conditions common in the population, but rare or nonaccessible in highly specialized teaching hospitals [29].

Some concerns have been raised about educational use of virtual patients. Virtual patients should not replace but complement contact with real patients [27]. There are concerns around the use of virtual patients potentially resulting in less empathic learners [30]. The use of unfamiliar technology as part of virtual patients’ education can represent a barrier to learning, even for younger generations [31,32]. Virtual patients may also prove ineffective when technological objectives drive teaching instead of being motivated by learning needs [33].

This virtual patient simulation review has been preceded by several narrative reviews [22,34-37] and 2 systematic reviews with meta-analyses [38,39]. Our preliminary literature analysis showed that the number of studies including the term *virtual patient* or *virtual patients* has more than doubled on the MEDLINE database in comparison with available evidence provided in previous systematic reviews (February 2009 [38] and July 2010 [39]). Thus, our review will update the evidence base with studies not included in previous analyses.

### Objectives

The objective of this review was to evaluate the effectiveness of virtual patient simulation for delivering pre- and postregistration health care professions education using the following comparisons:

1. Virtual patient versus traditional education
2. Virtual patient blended learning versus traditional education
3. Virtual patient versus other types of digital education
4. Virtual patient design comparison

By traditional education, we mean all nondigital educational methods. This includes lectures, reading exercises, group discussion in classroom, and nondigital simulation as standardized patients or mannequin-based training. Virtual patient blended learning is the addition of virtual patients as a supplement to traditional education when the control intervention uses nondigital education methods only. Other types of digital education may include interventions such as video recordings, Web-based tutorials, or virtual classrooms.

We assessed the impact of virtual patient interventions on learners’ knowledge, skills, attitude, and satisfaction. Our secondary objective was to assess the cost-effectiveness, patient outcomes, and adverse effects of these interventions.

## Methods

### Protocol and Registration

While conducting the review, we adhered to the Cochrane methodology [40], followed a published protocol [41], and presented results following the Preferred Reporting Items for Systematic Reviews and Meta-Analyses guidelines [42].

### Eligibility Criteria

We included randomized controlled trials (RCTs) and cluster RCTs (cRCTs). We excluded crossover trials because of the high likelihood of carryover effect.

Participants in the included studies had to be enrolled in a pre- or postregistration health-related education or training program (see glossary in Multimedia Appendix 1). This included students from disciplines such as medicine, dentistry, nursing and midwifery, medical diagnostic and treatment technology, physiotherapy and rehabilitation, and pharmacy.

This review focused on screen-based virtual patient simulations that form a computerized, dynamically unfolding representation of patient cases. A virtual patient simulation is introduced by a case description and might contain answers given by the patient, clinical data (eg, laboratory results, medical images), and descriptions of patients’ signs and symptoms. Only the representations of the patient as a whole were of interest, rather than studies that focused on single parts of the body. As a matter of a policy followed in the Digital Health Education Collaboration [6] and aiming at avoiding duplication of reviews, we deliberately excluded virtual patients in 3-dimensional (3D) virtual learning environments from this study. We judged that a higher level of immersion of learners in 3D virtual environments, connected with potential technical challenges (eg, difficulties in navigating such environments, lags because of increased computational time or limited internet bandwidth), was likely to influence the educational outcomes and therefore merited a separate analysis covered already by the virtual reality review [13] of this Digital Health Education Collaboration series. We also excluded those virtual patient interventions which require nonstandard equipment (eg, haptic devices, mannequins) or those virtual patients which are human controlled (eg, simulated email correspondence or chat room conversations). We excluded studies in which virtual patients were just a small part of the intervention and those in which the influence of virtual patients was not evaluated separately.

Furthermore, 2-arm RCTs comparing virtual patients with a control group not involved in any type of subject-related learning activity were not considered eligible as previous meta-analyses have already shown a large positive effect when virtual patients were compared with no intervention [38].

We decided to introduce to the review a comparison of virtual patients blended learning with traditional education as a consequence of the discussion in the community on the need to eliminate traditional types of learning activities to make space for virtual patients. For instance, Berman et al [29] noticed that the students’ subjective learning effect perceptions and satisfaction with integration were lower at universities that increased the workload of students by adding virtual patients without releasing time resources in the curriculum. As most of health professions education is conducted on campus, an integrated effect of virtual patients is possible. Blending virtual patients with traditional education is challenging and qualitatively different than a nonintervention control group comparison.

Eligible primary outcomes were students’ (1) knowledge, (2) skills, (3) attitudes, and (4) satisfaction—together representing clinical competencies measured post intervention with validated or nonvalidated instruments. Secondary outcomes were (1) economic cost and cost-effectiveness, (2) patient outcomes, and (3) observed adverse effects.

### Search Methods for Identification of Studies

We searched the following 7 databases: MEDLINE (via Ovid), EMBASE (via Elsevier), The Cochrane Library (via Wiley), PsycINFO (via Ovid), Educational Resource Information Centre (ERIC; via Ovid), Cumulative Index to Nursing and Allied Health Literature (CINAHL; via EBSCO), and Web of Science Core Collection (via Thomson Reuters). We adapted the MEDLINE strategy and keywords presented in Multimedia Appendix 2 for use with each of the databases above. We searched databases from the year 1990 to September 20, 2018 to highlight recent developments and did not apply language restrictions. For all included studies, we searched references lists and conducted author and citation searches. We searched lists of references from other identified relevant systematic reviews while running our electronic searches.

### Data Collection and Analysis

#### Data Selection, Extraction, and Management

The search results were combined in a single EndNote library (version X7; Thomson Reuters) [43]. Overall, 2 authors independently screened titles and abstracts to identify potentially eligible studies. In the next phase, full-text versions of these papers were retrieved and 2 review authors independently assessed these papers against eligibility criteria. We piloted data extraction to maximize consistency in the information extracted. Disagreements were resolved through discussion. A third review author was consulted to arbitrate when differences in opinion arose. All relevant data were extracted using a structured form in Microsoft Excel. We contacted study authors for crucial missing information, particularly if required to judge inclusion criteria and study outcomes.

#### Data Items

Information was extracted from each included study on (1) the characteristics of study participants (field of study; stage of education: pre/postregistered; year of study; and country where the study was conducted and its World Bank income category: high-income/low-and-middle-income country), (2) the type of outcome measure (type of tool used to measure outcome and information on whether the tool was validated), (3) the type of virtual patient intervention (topic and language of presented virtual patient simulations; information on whether the language of virtual patient was native to the majority of participants; source of virtual patient simulations: internal/external; was the study an individual or group assignment, and in case of group assignments, the number of students in a group; whether access to virtual patient simulation was from home or in a computer laboratory; number of virtual patient cases presented; time when the virtual patients were available; and duration of use of virtual patients), and (4) the type of virtual patient system (name of the system; navigation scheme: linear, branched, and free access; control mechanism: menu-based, keyboard, or speech recognition; feedback delivery and timing; and whether video clips where included in virtual patient cases). A glossary of the terms in use in the review may be found in Multimedia Appendix 1.

#### Measures of Treatment Effect

We reported the treatment effects for continuous outcomes as mean values and SDs post intervention in each intervention group, along with the number of participants and *P* values. As the studies presented data using different tools, the mean differences were recalculated into standardized mean difference (SMD). We interpreted the effect size as small (SMD=0.2), moderate (SMD=0.5), and large (SMD=0.8) effect sizes [40]. If studies had multiple arms and no clear main comparison, we compared the virtual patient intervention arm with the most common control arm, excluding the nonintervention and mixed-intervention controls. If that was impossible to decide, we selected the least active control arm. If multiple outcomes in the same category (knowledge, skills, attitudes, and outcomes) were reported, we selected the primary measure, and if that was impossible, we calculated the mean value of all measures. For papers that reported median and range for the outcomes, we converted these to mean and SD using methods described by Wan [44]. If a study did not report SD but provided CIs, we estimated SD from those using a method described in previous literature [40].

#### Data Synthesis and Analysis

Owing to the significant differences between studies, we employed a random-effects model in the meta-analysis using Review Manager (version 5.3; The Nordic Cochrane Centre) [45]. We displayed the results of the meta-analysis in forest plots and evaluated heterogeneity numerically using I^2^ statistics. For comparisons with more than 10 outcomes in the meta-analysis, we attempted a subgroup analysis. As the planned 15 subgroup analyses in the protocol [41] did not explain the heterogeneity, we visualized the outcomes using albatross plots [46]. These plots were implemented using a script created for the purpose of the study by one of the review authors (AK) in the statistical package R (version 3.4.3; R Foundation for Statistical Computing) [47]. This explorative approach resulted in a new subgroup analysis in which we divided the control interventions into active (group discussion, mannequin-based simulation) and passive (lectures, reading assignments). Findings unsuitable for inclusion in a meta-analysis (eg, comparison of individual items in surveys) were presented using a narrative synthesis.

#### Assessment of Risk of Bias

Two authors independently assessed the risk of bias using the Cochrane tool [40]. We considered the following domains: random sequence generation, allocation sequence concealment, blinding of participants or personnel, blinding to outcome assessment, completeness of outcome data, selective outcome reporting, and other sources of bias (eg, differences in baseline evaluation, volunteer bias, commercial grants). For cRCTs, we also assessed the risk of the following additional biases: recruitment bias, baseline imbalance, loss of clusters, incorrect analysis, and comparability with individually randomized trials. The publication bias in our review was difficult to investigate in a formal way because of high levels of heterogeneity which limit the interpretation possibilities of funnel plots.

#### Summary of Findings Tables

We prepared *summary of findings* tables to present results of the meta-analysis [40]. We presented the results for major comparisons of the review and for each of the major primary outcomes. We considered the Grading of Recommendations Assessment, Development and Evaluation (GRADE) criteria to assess the quality of the evidence and downgraded the quality where appropriate [40].

## Results

### Included Studies

**Figure 1:**
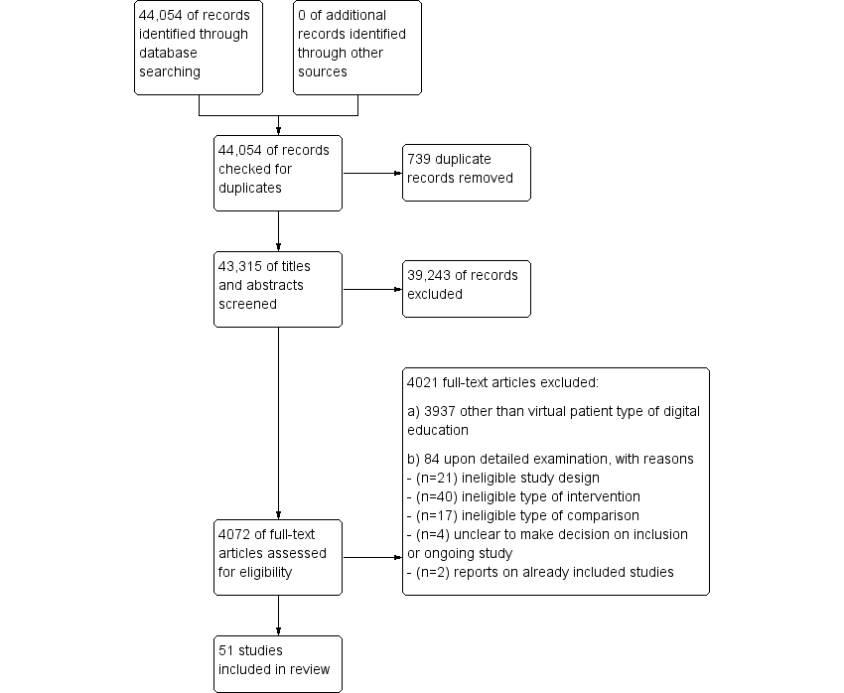
Preferred Reporting Items for Systematic Reviews and Meta-Analyses (PRISMA) study flow diagram.

Our searches yielded a total of 44,054 citations, and 51 studies with 4696 participants were included (Figure 1). Overall, 2 reports described results already included in the review [48,49].

### Types of Studies

All included studies were published in peer-reviewed journals. All included studies had an RCT design, with the exception of 3 cRCTs [50-52].

### Types of Comparisons

A total of 25 studies compared virtual patients with traditional education [53-77], 11 compared a blend of virtual patients and traditional education with traditional education [50,52,78-86], 5 studies compared virtual patients with different forms of digital health education [51,87-90], and 10 studies compared different types of virtual patient interventions [91-100].

The traditional education control group involved a reading assignment in 6 studies [59,60,62,67-69]; 4 studies each involving a lecture [63,66,72,77], group assignment [53,65,71,73], and mannequin-based training [58,64,70,76]; and 1 each involving standardized patients [74] and ward-based education [75]. In 5 studies, the intervention was a mix of different forms of traditional education (eg, lecture, small group assignment, and mannequin-based training) [54-57,61].

The digital education control group was in 2 studies—a Web tutorial or course [88,90] and video recording [87,89]—and in 1 study, a mix of traditional lectures and Web materials including video clips [51] was used.

Studies comparing different types of virtual patients contrasted narrative with problem-solving structure of virtual patients [91]; virtual patients with and without usability enhancements [94]; different forms of feedback in virtual patients [95,96]; worked with unworked versions of virtual patients [97]; differences between self-determined and mandatory access to virtual patients [98]; virtual patients collections in which all the cases were presented at once to those automatically activated spaced in time [99]; effects of virtual patient solving with virtual patient construction exercises [100]; linear versus branched design of virtual patients [92]; and finally the addition of representation scaffolding (see glossary in Multimedia Appendix 1) to virtual patients [93].

Furthermore, 41 studies had 2 study arms (see the first table in Multimedia Appendix 3), 7 studies had 3 arms [62,88,91,95-98], and 3 studies had 4 arms [63,65,67].

### Types of Participants

In total, 41 studies involved preregistered professionals (see the first table in Multimedia Appendix 3), with 8 studies focused on postregistered participants [68,69,74,76,78,90,94,97]; 2 studies involved both pre- and postregistered participants [59,87].

In 37 out of 51 studies, participants were from the field of medicine. The studies from fields other than medicine were as follows: 6 studies in nursing [58,64,70,73,78,80]; 2 in pharmacy [53,92]; and 1 each in physical therapy [61], osteopathic medicine [84], and dentistry [83]. In addition, 3 studies involved interprofessional education [74,76,90].

A total of 44 out of 51 studies were conducted in high-income countries; 19 were from the United States (see the first table in Multimedia Appendix 3); 5 from Germany [65,82,93,98,99]; 3 each from Australia [52,70,91] and Sweden [83,85,87]; 2 each from Canada [59,60], the Netherlands [86,88], and the United Kingdom [66,77]; and 1 study was conducted each in Belgium and Switzerland [92], Denmark [100], France [54], Hong Kong [51], Japan [67], Poland [50], Singapore [64], and Slovenia [71]. From the 7 studies conducted in low-and-middle-income countries, 3 were from China [63,73,80], 2 from Colombia [55,56], and 1 each from the Republic of South Africa [94] and Iran [75].

In Multimedia Appendix 4 we present the technical characteristics of virtual patient systems, topics of educational content presented, applied instructional design methods, setting of use, information on the validity of outcome measurement, and applied educational theories in the included studies. Multimedia Appendix 5 summarizes the reasons for excluding studies following a review of their full-text versions.

### Effects of Interventions

#### Knowledge

In total, 33 studies assessed outcomes of knowledge. In all studies, knowledge was measured using paper-based tests (see the second table in Multimedia Appendix 3). In 19 studies, the test consisted of multiple-choice questions (MCQs). Other knowledge test designs contained multiple-response questions [100], true/false questions [50], and key feature format questions [82]. In 4 studies, the participants had to formulate free-text answers [75,85,94,99]. In 4 studies [63,66,93,97], the knowledge tests comprised a mix of different formats. Li et al [63] used a combination of multiple-choice and short answer questions; Miedzybrodzka et al [66] used MCQs and modified essays; Harris et al [97] applied MCQs with confidence levels combined with script concordance testing questions; and Braun et al [93] used a test consisting of multiple-choice items, key feature problems, and problem-solving tasks. Secomb et al [70] measured cognitive growth using a survey requiring selection of the most significant items regarding learning environment preferences. In 3 studies [62,73,92], the nature of the knowledge test was unclear. In the case of MCQs in which the nature of items was unclear or mixed, we classified the outcome as knowledge instead of, for example, clinical reasoning skills, but the borderline between those was sometimes blurred. Meta-knowledge (eg, knowledge about the clinical reasoning process itself) was classified as knowledge outcomes following the framework by Kraiger [101].

The effects of interventions on knowledge outcomes are summarized in the second table in Multimedia Appendix 3.

##### Virtual Patient Versus Traditional Education

In 4 [60,63,67,72] of 18 studies comparing virtual patients with traditional education, the intervention resulted in more positive knowledge outcomes. In 2, the control group attended a lecture [63,72], whereas in the remaining 2 studies, students participated in a reading exercise [60,67]. In 1 study [53], the control intervention arm (Problem-based learning (PBL) small group discussion) had significantly better results than the virtual patient intervention (SMD=−0.65, 95% CI −1.02 to −0.28, *P=*.001). In the remaining 13 studies, the difference did not reach a statistically significant level (see the second table in Multimedia Appendix 3).

We excluded 2 studies [62,65] from our meta-analysis because of missing crucial outcome data. Jeimy et al [59] presented outcomes of a knowledge test compared item-by-item and the study was therefore excluded from the meta-analysis. We also excluded 1 study [72] owing to its outlier value of SMD=12.5 being most likely because of reporting error and excluded another study [70] as we regarded meta-knowledge as very different from the other types of core knowledge outcomes.

**Figure 2:**
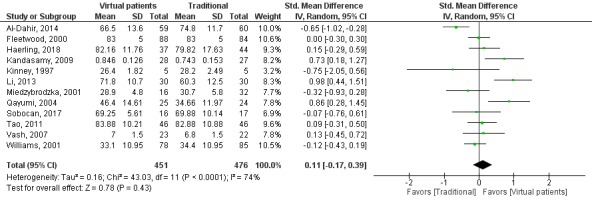
Forest plot of virtual patient to traditional education comparison for knowledge outcomes. df: degrees of freedom; IV: interval variable; random: random effects model.

The pooled effect for knowledge outcomes (SMD=0.11, 95% CI −0.17 to 0.39, I^2^=74%, n=927; Figure 2) suggests that virtual patient interventions are as efficient as traditional education.

##### Virtual Patient Blended Learning Versus Traditional Education

In 4 [50,52,80,82] of the 5 studies comparing virtual patients as a supplement with traditional education in the domain of knowledge, the group having the additional resource scored better than the control group. Only in 1 study [85] did the addition of virtual patients not lead to statistically significant difference in knowledge outcomes (*P*=.11).

**Figure 3:**
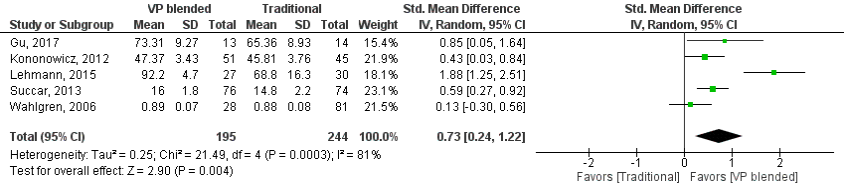
Forest plot of virtual patient blended learning to traditional education comparison for knowledge outcomes. df: degrees of freedom; IV: interval variable; random: random effects model; VP: virtual patients.

The pooled effect for knowledge outcomes (SMD=0.73, 95% CI 0.24 to 1.22, I^2^=81%, n=439; Figure 3) suggests moderate effects preferring the mix of virtual patients with traditional education over traditional education alone.

##### Virtual Patient Versus Other Types of Digital Education

A total of 2 studies compared the difference in knowledge outcomes between virtual patients and digital health education interventions. Courteille et al [87] compared virtual patients with a video-recorded lecture, whereas Trudeau et al [90] compared with a static Web course. Neither of these comparisons showed significant differences in knowledge outcomes.

##### Virtual Patient Design Comparison

In total, 8 studies focused on detecting the difference between variants of virtual patient design in the domain of knowledge. Only in 1 study by Friedman et al [96] were the differences at a statistically significant level. In this study, the pedagogic design of virtual patients was better than problem-solving and high-fidelity designs (*P*<.01). Comparing linear and branched virtual patients [92], scaffolded versus nonscaffolded [93], worked and unworked examples [97], virtual patient with usability extensions [94], self-determined versus mandatory integration [98], spaced versus nonspaced release of cases [99], and virtual patient solving versus virtual patient design exercises [100] resulted in no significant differences in knowledge outcomes.

#### Skills

A total of 28 studies assessed skills outcomes (see the third table in Multimedia Appendix 3). Skills were assessed by performance on a mannequin in 9 studies [50,54,64,68,69,73,80,82,88], by performance on a live standardized patient in 8 studies [57,67,78,81,89,91,95,100], and performance on virtual patients [93] and real patients [83] in 1 study each. In 6 studies, outcomes were measured by a written assignment involving description of photographed clinical cases [63], radiographs [65], carrying out and structuring a mental state examination based on videotaped material [77], solving paper cases [74,86], and a modular paper-based test [75]. In 2 studies [55,56], outcomes were measured by a mix of paper cases and virtual patients. Kumta et al [51] combined computer-based assessment, objective structured clinical examination (OSCE), and clinical examination comprising patients in the ward into 1 score.

The effects of interventions on skills outcomes are summarized in the third table in Multimedia Appendix 3.

##### Virtual Patient Versus Traditional Education

In 9 of 14 studies comparing virtual patients with traditional education, the intervention resulted in better skills outcomes (see the third table in Multimedia Appendix 3). The virtual patient intervention showed larger effects than lectures [63,77], reading exercises [67-69], group discussions [73], and activities comprising traditional methods, including lectures or hands-on training with mannequins [54-56].

Those skills which improved were clinical reasoning [55,56,63,77], procedural skills [54,67-69], and a mix of procedural and team skills [73].

We did not include in the meta-analysis 2 studies with incomplete reported data [65,74]. We also excluded skills outcomes from the study by Haerling [58] as these were available for a randomly selected subgroup only and from Wang et al [76] as they were measured for teams of students only and not individually.

**Figure 4:**
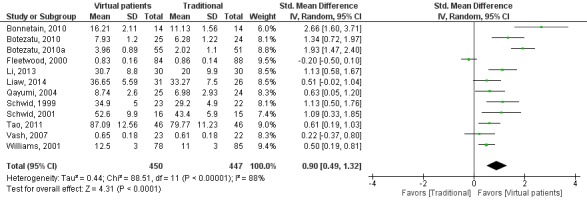
Forest plot of virtual patient to traditional education comparison for skills outcomes. df: degrees of freedom; IV: interval variable; random: random effects model; VP: virtual patients.

The pooled effect on skills outcomes was (SMD=0.90, 95% CI 0.49 to 1.32, I^2^=88%, n=897; Figure 4). Overall, this suggests that virtual patients have moderate to large positive effects in comparison with traditional education in the investigated types of skills.

##### Virtual Patient Blended Learning Versus Traditional Education

In 3 [82,83,86] out of 7 studies, the groups using virtual patients blended learning scored better than the control group in the skills domain. Lehmann et al [82] demonstrated significantly improved procedural skills (*P*<.001), whereas Weverling et al [86] reported on improved clinical reasoning skills (*P*<.001) and Schittek Janda et al [83] on communication skills (*P*<.01). Furthermore, 2 studies [78,80] involving nursing students showed no significant difference. The study by Bryant et al [78] evaluated communication skills (*P*=.38), whereas Gu et al [80] measured procedural skills (*P*>.05). We excluded 3 studies [50,81,83] from the meta-analysis because of insufficient data provided in the report or item-by-item comparison of a skills checklist.

**Figure 5:**
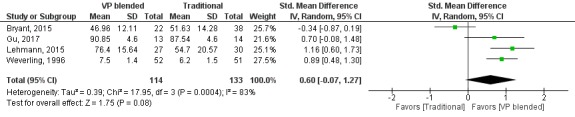
Forest plot of virtual patient blended learning to traditional education comparison for skills outcomes. df: degrees of freedom; IV: interval variable; random: random effects model; VP: virtual patients.

The pooled effect for skills outcomes (SMD=0.60, 95% CI −0.07 to 1.27, I^2^=83%, n=247; Figure 5) suggests that virtual patients blended with traditional education have moderate positive effects in comparison with traditional education alone.

##### Virtual Patient Versus Other Types of Digital Education

Out of 3 studies comparing skills outcomes in virtual patients with other types of digital education studies, Kumta et al [51] showed a significant difference (*P*<.001). In this study, virtual patients were better than a range of different traditional teaching methods supplemented by Web content that included video clips, PowerPoint presentations, digital notes, and handouts. The target outcomes were clinical skills assessed by OSCE stations and examination of patients in the wards. In a study by Dankbaar et al [88], virtual patients were not significantly better in teaching procedural skill than an electronic module only (*P*>.05). Finally, in the study by Foster et al [89], virtual patients showed no significant difference when compared with video recordings in teaching communication skills (*P*>.05).

##### Virtual Patient Design Comparison

From the 4 studies that compared the influence of different virtual patient designs on skills outcomes, Foster et al [95] showed that virtual patients with emphatic feedback were significantly better in training communication skills than those virtual patients without feedback (*P*<.03). In the study by Bearman et al [91], narrative virtual patients were significantly better than problem-solving virtual patients in conveying communication skills (*P*=.03). In a study by Braun et al [93], the addition of representational scaffolding to a virtual patient intervention significantly improved diagnostic efficiency (*P*=.045). Finally, in the study by Tolsgaard et al [100], there was no significant difference in integrated clinical performance when students constructed or solved virtual patients (*P*=.54).

### Attitudes

A total of 11 studies reported attitudinal outcomes (see the third table in Multimedia Appendix 1). The attitudes related to confidence, preparedness, comfort, self-efficacy, and perceived ability in topics such as history taking and clinical breast examination [79], diagnostic and management abilities [59], contrast reaction management and teamwork [76], ethical, legal, and communication issues [57,81], opioid therapy [90], cultural competence [84], procedural knowledge in pediatric basic life support [82], performing pharmacy triage [92], caring for distress disorders patients [74], and anxiety [77].

The effects of interventions on attitudinal outcomes are presented in the fourth table in Multimedia Appendix 3. Furthermore, 3 studies presented pooled scores on students‘ self-assessment. In the study by Lehmann et al [82], students felt more confident in their knowledge and skills on performing pediatric basic life support with additional access to virtual patients that supplemented their traditional course (*P*<.001). There were no significant differences in the remaining 2 studies focusing on communication-related self-efficacy [81] and attitudes related to opioid therapy [90].

In the study by Williams et al [77], more items related to self-assessment of competences (in dealing with ethical aspects and managing anxiety) were scored lower in the virtual patient group than in the traditional education groups. There were no differences in analyzed items related to attitudes in 4 studies [57,59,74,79]. In the study by Smith et al [84], the results regarding attitudes toward clinical cultural competence were presented separately for bilingual and English-speaking students, which makes it difficult to aggregate not knowing the number of bilingual students in each study group. However, the descriptive conclusion of the authors was that general cultural competence measures were the same for the virtual patient and control group. In 2 studies [76,92], the results were compared item-by-item and only within the groups (pre/posttest), not between the study groups.

### Satisfaction

In total, 17 studies measured satisfaction resulting from an intervention (see the fifth table in Multimedia Appendix 3). All outcomes in this category were measured by satisfaction questionnaires. Different facets of satisfaction were measured, which we classified in the following 5 dimensions: general impression (global score or willingness to recommend), comfort in use (learning style preference, engagement or motivation, positive climate or safety, and enjoyment or pleasure), integration in curriculum (time constraints, relevance, and level of difficulty), academic factors (feedback quality, structure, and clarity), and satisfaction with technical features (usability and information technology readiness).

In 4 out of 17 studies evaluating the satisfaction of students receiving a virtual patient intervention, the result was presented as 1 aggregated score of several items. Furthermore, 3 of those studies compared different design variants of virtual patients. In the study by Friedman et al [96], the pedagogic format (menus, guided) resulted in higher satisfaction scores than the high-fidelity (free text, unguided) format (*P*<.01). There was no statistically significant difference between the virtual patients with and without usability enhancements [94] (*P*=.13) and solving versus constructing virtual patients [100] (*P*=.46). One study [58] presented comparison of virtual patients with mannequin-based training using a single score for student satisfaction and self-confidence in learning, showing no difference between the simulation modalities (*P*=.11).

In the remaining 13 out 17 studies, the survey responses were compared item-by-item. In 4 studies, the majority of the items indicated preference for the virtual patient intervention, in comparison with lecture [63], reading assignment [67], video-based learning [89], and Web tutorial [88]. In 7 studies, most items were indifferent between the groups [53,59,62,65,66,74,92]. In 1 study [76], most items (5 out of 6) in a satisfaction survey were better rated in the mannequin-based training than in the virtual patient group.

### Secondary Outcomes

One study had cost-effectiveness as an outcome [58]. In 9 studies, statements were made regarding the cost of the intervention—either monetary or in development time [53,60,62,64-66,79,95]. Only 1 study provided numerical data on both types of intervention [95]. The comparison was qualitative in 3 studies [64,65,78]. In 5 studies, estimations of costs were made for the virtual patient group without contrasting it with the cost of the control intervention [53,60,62,66,79]. None of the included studies had patient outcomes or adverse effects as the main outcome measure. Even though none of the studies reported direct patient outcomes, in 2 studies, the participants were observed by raters while performing tasks on real patients as an outcome assessment [51,83]. In the study by Kumta et al [51], the score was included in more complex assessment (including MCQ tests and OSCE examination) and the patient-related outcome was not explicitly reported. In the study by Schittek Janda et al [83], first year students of dentistry were asked to perform history taking with real patients and were rated by the instructor. The patients’ perspective was, however, not considered. Even though none of the studies had adverse effects as the major outcome, 6 studies [53,55,67,70,84,88] reported findings related to noticed unexpected effects of the intervention.

#### Cost

Haerling [58] showed a better cost-utility ratio of US $1.08 for virtual patients versus US $3.62 for the mannequin-based training. Foster et al [95] compared the cost of human-provided (Mechanical Turk) feedback with backstory video feedback; the cost of human answers was US $0.05 per question assisted, whereas videos required 4 hours of development time and the license cost of a video game (Sims 3 by Electronic Arts). This does not provide a direct answer to the question of which method was more cost-efficient as it depends on the number of participants and time of use. It is also important to notice that the human-generated feedback in virtual patients showed positive effects on the communication skills outcomes, whereas the backstory video did not. Bryant et al [78] estimated, but without providing numerical evidence, that the cost of a virtual patient was similar to that of a course text that was eliminated by the new intervention. Liaw et al [64], without providing concrete numbers, noticed that despite “initial startup costs for developing the virtual patient simulation, its implementation was less resource intensive than the mannequin-based simulation.” The cost savings were because of reduced instructor time, use of expensive equipment, or simulation facilities. Maleck et al [65] saw cost savings in the virtual patient group because of spared radiograph printouts. The cost of the virtual patient intervention was expressed in hours of work; in 2 cases, the cost was 12 to 15 hours per virtual patient [53,60]; in 1 case it was 15 to 30 hours [62] and 100 hours in another [66]. The cost expressed in amounts of money was estimated at US $500 for content development and technical implementation [62] and US $4800 for a total clerkship restructuring, including adding virtual patients [60]. It is worth noticing that in both cases the virtual patients were developed by students. Deladisma et al [79] used in their study a virtual patient system that involved a speech recognition engine, tracked user’s body movements, and projected a life-sized avatar on the wall. The cost of the technology used in the pilot study (including 2 networked personal computers, 1 data projector, and 2 Web cameras) was estimated in 2006 to be less than US $7000 [102].

#### Patient Outcomes

In the study by Schittek Janda et al [83], an experienced clinician rated the professional behavior (language precision, order of question, and empathy) of first year students’ of dentistry toward real patients as significantly higher (*P*<.01) in the group having access to a supplementary virtual patient case than in the group that underwent standard instruction.

#### Adverse Effects

Dankbaar et al [88] hypothesize based on their study results that high-fidelity virtual patients may increase motivation, but at the same time be more distracting for novice students and by that impede learning. Authors of 2 studies [70,84] observe that the language of virtual patients might be a significant factor showing greater effects on nonnative English speaking and bilingual learners than in native English speakers. In the study by Qayumi et al [67], it is observed that that lower-achieving students benefit more from virtual patients than high performers. In the study by Botezatu et al [55], students knowing about the possibility of being assessed by virtual patients opposed being tested with paper cases. In the study by Al-Dahir et al [53], it is observed that analysis of individual learner traces in the virtual patient system negates benefits of social learning.

### Subgroup Analysis

None of the initially planned subgroup analyses explained the heterogeneity of the results.

Among many analyzed aspects, we looked into differences regarding the efficiency of learning with virtual patients between the health professions disciplines. Most of the located studies involved students of medicine as participants. For instance, when comparing virtual patients with traditional education in the domain of skills, out of the 12 outcomes included for subgroup analyses, only 2 were from other health profession disciplines than medicine (ie, studies from nursing [64,73]). When analyzing knowledge outcomes out of the 12 included studies, 4 were nonmedical but represented 3 very different disciplines, nursing [58,73], pharmacy [53], and physiotherapy [61]. The conducted subgroup analyses showed no significant differences between the subgroups and high heterogeneity.

While analyzing aspects of instructional design implemented in the virtual patient scenarios, we were able to locate a very balanced number of studies implementing the narrative and problem-solving designs [91] in the domain of knowledge outcomes (6 studies in each branch). Yet, the pooled results showed no difference (narrative: SMD=0.12, 95% CI −0.41 to 0.64, I^2^=85%, n=525 versus problem solving: SMD=0.11, 95% CI −0.17 to 0.38, I^2^=51%, n=520; subgroup differences *P*=.97). Interestingly, when looking into the domain of skills outcomes, all studies had either the problem-solving or unclear design (in 2 cases). This might be an indication that narrative (linear, branched) virtual patients are seen as being better suited for knowledge outcomes rather than skills.

**Figure 6:**
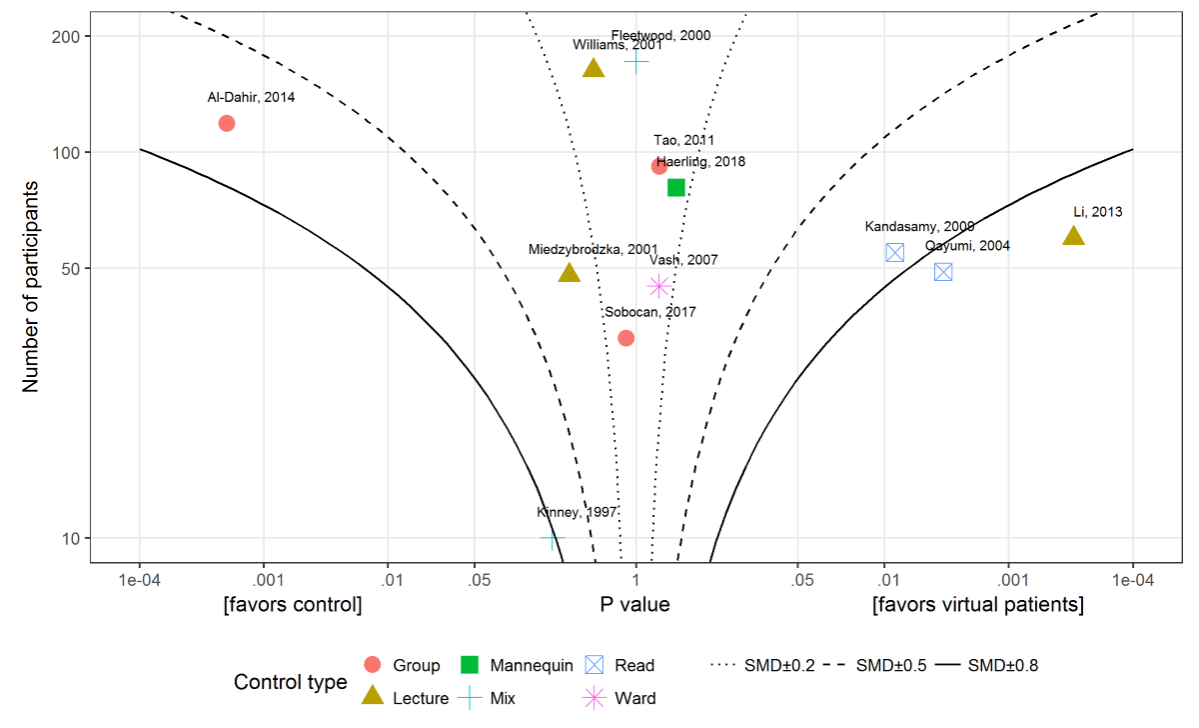
Albatross plot for studies comparing virtual patient with traditional education for knowledge outcomes. SMD: standardized mean difference.

Finally, we were unable to see any pattern in efficiency when analyzing the timing of feedback as being either during activity or post activity. However, in almost half of the studies, we were unable to decide, based on the description of the intervention, which model of feedback was implemented or whether the study had a mixed (during/post activity) mode of providing feedback.

**Figure 7:**
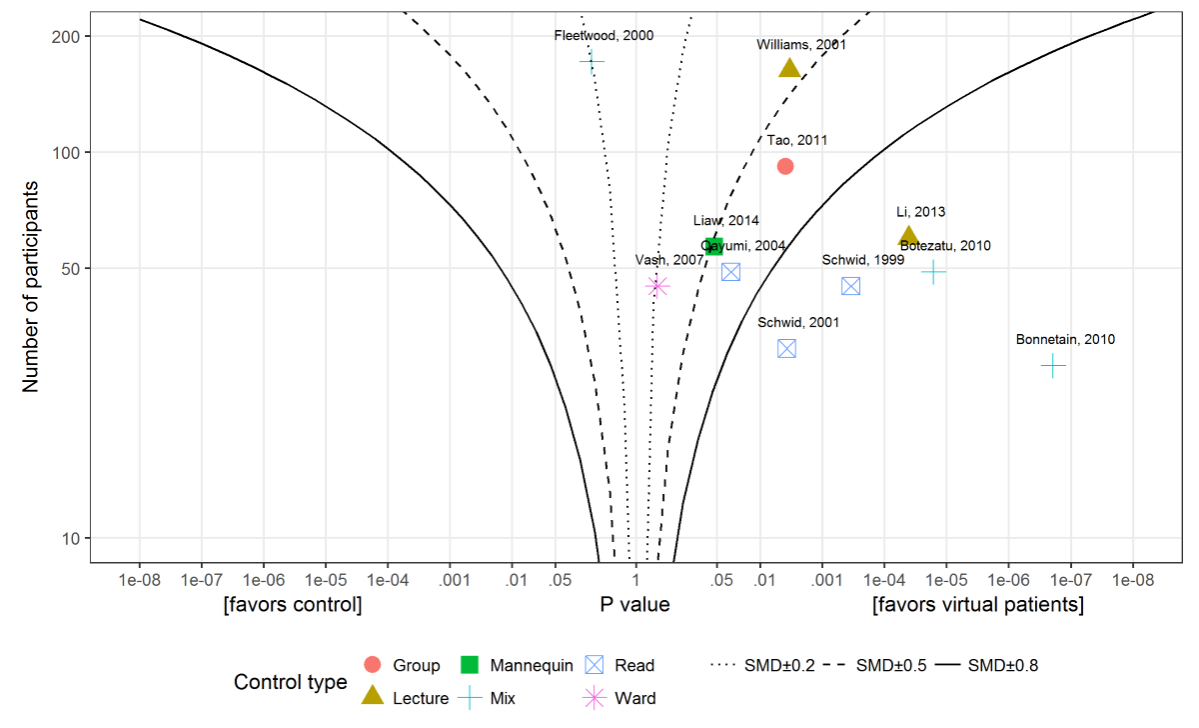
Albatross plot for studies comparing virtual patient with traditional education for skills outcome. SMD: standardized mean difference.

To further explore the reasons for heterogeneity, we visualized the outcomes in the form of albatross plots of the knowledge and skills outcomes for virtual patients to traditional education comparisons. Figure 6 presents an albatross plot for knowledge and Figure 7 for skills outcomes. Comparisons of virtual patients to passive forms of learning (reading exercises and lectures) tended to display large positive effect sizes, whereas those comparing virtual patients to active learning (group discussion or mannequin-based learning) show small effects or even negative effects (left hand side in the Figures 6 and 7 and Multimedia Appendix 6).

### Risk of Bias

**Figure 8:**
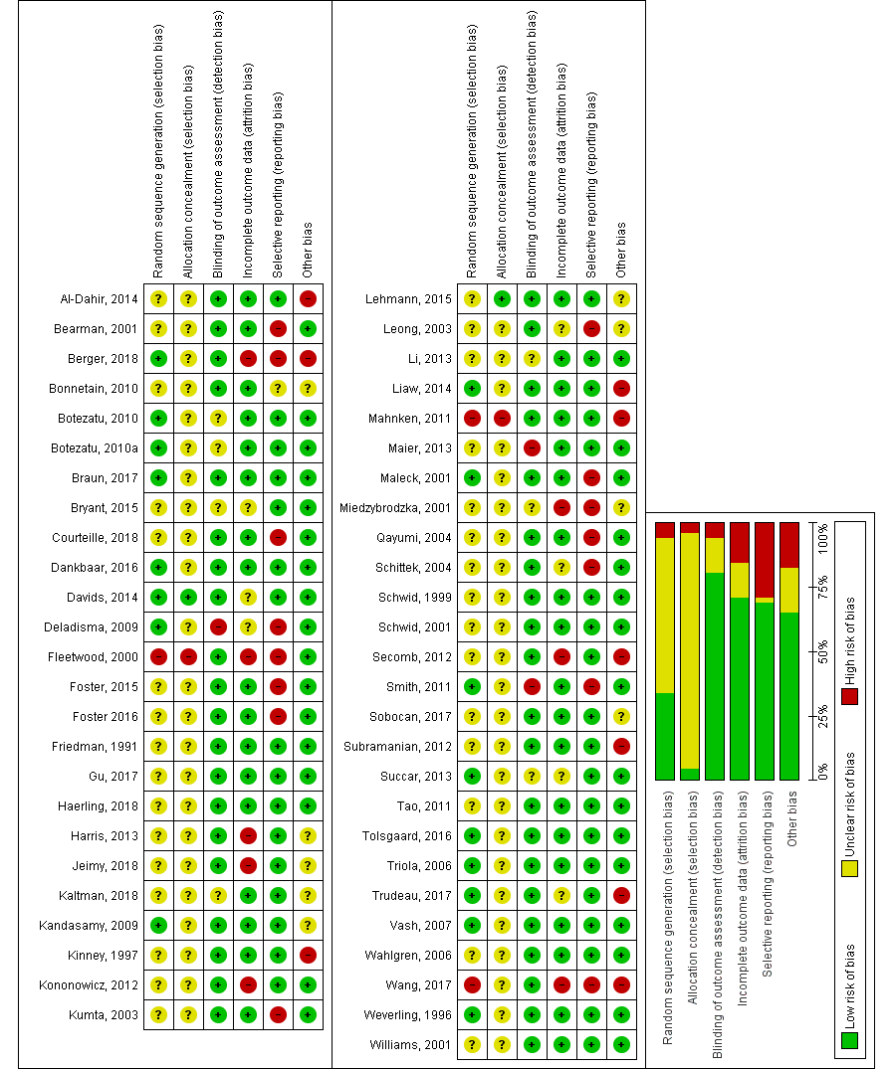
Risk of bias summary (+ low risk of bias; - high risk of bias, ? unclear risk of bias).

Following the Cochrane methodology [40], we have assessed the risk of bias in all included studies. The results of the analysis are summarized in Figure 8.

Overall, we do not consider allocation bias as a significant issue in the review as most of the studies either described an adequate randomization method (17 of 51 studies) or even when the description was unclear (31 of 51), it was judged unlikely that the randomization was seriously flawed. Performance bias in comparisons with traditional education is an issue but at the same time is impossible to avoid in this type of research. The blinding of participants in virtual patient design comparisons is possible, but those studies are still relatively uncommon (n=10). The risk of assessor bias was avoided in many studies by using automated or formalized assessment instruments. Consequently, we assessed the risk as low in 42 of 51 studies. However, it is often unclear whether the instruments (eg, MCQ tests, assessment rubrics) were properly validated. We felt that in the majority of studies, attrition bias was within acceptable levels (low risk in 36 of 51 studies). This does not exclude volunteer bias, which is likely to be common, but its influence is difficult to estimate. As there is little tradition of publishing protocols in medical education research, it was problematic to assess selective reporting bias, but we judged the risk as low in 35 out of 51 studies. We were unable to reliably assess publication bias considering the high heterogeneity of studies. None of the cRCT studies considered in the statistical analysis had corrections for clustering, but we have decreased the number of participants in those studies using a method from the Cochrane Handbook to compensate for that. We present more details of the risk of bias analysis in Multimedia Appendix 7.

We rated down the quality of evidence for knowledge and skills outcomes in virtual patients to traditional education comparison because of the high heterogeneity of included studies and limitations in study design (lack of participant blinding, nonvalidated instruments, and potential volunteer bias). For attitudinal and satisfaction outcomes and for other types of comparisons, we additionally rated down the quality as the outcomes were presented as independent items in questionnaires that were not amenable to statistical analysis or the analyses contained just a handful of studies and the CIs were wide. Summary of findings table (GRADE) are presented in Multimedia Appendix 8.

## Discussion

### Principal Findings

The aim of this review was to evaluate the effectiveness of virtual patients in comparison with other existing educational methods.

There is low quality evidence that virtual patients are at least as effective as traditional education for knowledge outcome and more effective for skills outcomes. On the basis of the visual analysis of albatross plots, we may hypothesize that replacing passive forms of traditional education with virtual patients brings more benefit than replacing active learning methods. We collected positive evidence of effectiveness from both high-income and low-and-middle-income countries demonstrating the global applicability of virtual patients. Students were generally satisfied with the use of virtual patients, but we also located studies in our review where the use of virtual patients was connected with diminished confidence.

The strength of our systematic review is the broad perspective which shows the landscape of RCTs in the domain of virtual patients. Our systematic review updates the evidence on virtual patient effectiveness, which was last summarized in a meta-analysis almost a decade ago.

### Limitations

The limitation of our work is that the wide scope of the review does not allow nuances in the studies to be explored in detail. We were unable to make a firm assessment of publication bias. The high heterogeneity of the results leads to the conclusion that without further consideration of needs and implementation details, we cannot expect that the introduction of virtual patients will always lead to detectable positive outcomes. Evidence to determine the effective factors is sparse and represented by only 10 studies in our review, with very diverse research questions.

Our review is limited by the decision to exclude crossover design studies. However, this has been discussed in detail in the potential biases in the review process section in Multimedia Appendix 7. We excluded studies published before 1991 as we consider the technology available before the World Wide Web to be materially different from that currently available. Finally, we are limited by the sparse description of the interventions in some of the papers, which occasionally might have led to misclassification of the studies.

### Comparison With Prior Work

Extending the results of the meta-analysis by Cook et al [38] and in agreement with the one by Consorti et al [39], our review shows that virtual patients have an overall positive pooled effect when compared with some other types of traditional educational methods. Our observations regarding the influence of the type of outcome (knowledge/skills) and comparison (active/passive traditional learning) supplement the evidence in the previous reviews [38,39], which included studies until 2010. This time point divides the evidence collected in 2 parts: (1) time already covered by previous reviews (1991-2010) and (2) time not included in the previous reviews (2011-2018). It is interesting to note that, though the former timeframe spans over 20 years compared with 8 years in the latter, more studies were included from the latter period, 22 studies (until 2010) versus 29 studies (after 2010). This demonstrates increased interest in virtual patients and medical education in general. The research community around digital health education has long been criticized for publishing media-comparative studies [103,104]. Media-comparative research aims to make comparisons between different media formats such as paper, face-to-face, and digital education [104]. Both Friedman and Cook argue [103,104] that the limitations of this type of comparison boils down to the inability to produce an adequate control group as interventions are bound to be influenced by too many confounding factors to be generalizable. Even though there are still many media-comparative studies, the number of studies comparing different forms of digital education seem to increase: 3/22 (14%) until 2010 versus 11/29 (38%) after 2010. The number of studies in which students worked from home as an intervention has also increased; before 2011 there was just 1/16 (6%; in 6 studies it was unclear), whereas after 2011, it was 11/22 (50%; in 7 studies it was unclear) studies. However, this potentially raises concerns about how controlled the interventions and measures were, and thus the validity of the conclusions.

Our observation that virtual patient simulations predominantly effect skills rather than knowledge outcomes can be interpreted as an indication that for lower levels of Bloom's taxonomy [105], (*remember, understands*) there is little added value of introducing virtual patients when compared with traditional methods of education. Virtual patients can have greater impact when applied where knowledge is combined with skills and applied in problem solving, and when direct patient contact is not yet possible. We found little evidence to support the use of virtual patients at higher levels of the taxonomy. We also warn against using our result in justifying diminished hours of bedside teaching as this was investigated in just 1 study [75] and did not show positive outcomes. Consequently, virtual patients can be said to be a modality for learning in which learners actively use and train their clinical reasoning and critical thinking abilities before bedside learning, as was previously suggested in their critical literature review by Cook and Triola [22].

The perceptions of students toward studying with virtual patients are generally positive. However, some exceptions can be noted. In 1 study [77], students were less confident in their skills when compared with facilitated group discussion and lecture. This is in contrast with no observable differences or even better performance in the virtual patient group when considering the objective outcomes in those studies. This could be explained by disbelief in the effectiveness of the new computer-based methods of learning or anxiety of losing direct patient contact.

The results of our subgroup analysis, though inconsistent, encourage the introduction of more active forms of education. Yet, we note that the range from active to passive learning forms a continuum, and the decision on how to classify each intervention is hampered by sparse descriptions in the reports. Nevertheless, questioning the utility of passive learning is not a new finding and is observed elsewhere, for instance, in the literature on the flipped-classroom learning approach [106]. As the effects of comparing virtual patients with other forms of active learning were small and we could not detect any other variables explaining the heterogeneity, it seems reasonable to individually consider other factors such as cost of use, time flexibility, personnel shortage, and availability in different settings (eg, students’ homes or locations remote from academic centers) when determining which methods to use.

The need for more guidance within virtual patient simulations is apparent in studies differing by instructional methods where narrative virtual patient design was better than more autonomous problem-oriented designs [91]. Feedback given by humans at distance in a virtual patient system was better than an animated backstory in increasing empathy [95], whereas more active constructing virtual patients with more time on a task but no feedback had no more positive result on the outcomes than learning from a virtual patient scenario [100]. This reminds us that presenting realistic patient scenarios with a great degree of freedom cannot be an excuse for neglecting guidance in relation to learning objectives [107,108].

### Outlook

We join the plea of Friedman [103] and Cook [104] to abandon media-comparative research as it is difficult to interpret and we instead encourage greater focus on exploring the utility of different design variants of virtual patient simulations. The current knowledge on the influence of these factors is sparse. A carefully planned study backed up in sound educational theory should provide many valuable research opportunities. However, sufficiently powered samples are needed, as the effects are likely to be small. The second consideration pertains to the need to use previously validated measurement tools that are well-aligned with the learning objectives. Comparisons of outcomes in tools on an item-by-item basis is methodologically questionable and makes the aggregations of results difficult in systematic reviews. We also call for more studies in other health professions disciplines than medicine, as our subgroup analysis showed that evidence of virtual patient effectiveness in such programs as nursing, physiotherapy, or pharmacy is underrepresented. Investigations into patient outcomes and cost-effectiveness of virtual patients are not yet explored directly and form a key avenue for future efforts.

### Conclusions

Low to modest and mixed evidence suggests that when compared with traditional education, virtual patients can more effectively improve skills, and at least as effectively improve knowledge outcomes as traditional education. Education with virtual patients provides an active form of learning that is beneficial for clinical reasoning skills. Implementations vary and are likely to be broad across pre- and postregistration education, although current studies do not provide clear guidance on when to use virtual patients. We recommend further research be focused on exploring the utility of different design variants of virtual patients.

## Appendix

Multimedia Appendix 1 Glossary.

Multimedia Appendix 2 MEDLINE (Ovid) search strategy.

Multimedia Appendix 3 Summary of included studies.

Multimedia Appendix 4 Summary of technical and educational features of included studies.

Multimedia Appendix 5 Summary of excluded studies.

Multimedia Appendix 6 Subgroup analysis details.

Multimedia Appendix 7 Quality of the evidence.

Multimedia Appendix 8 Summary of finding tables.

## Abbreviations

3D: 3-dimensional
cRCT: cluster randomized controlled trials
GRADE: Grading of Recommendations Assessment, Development and Evaluation Working Group
MCQ: multiple-choice question
OSCE: objective structured clinical examination
RCT: randomized controlled trial
SMD: standardized mean difference
