# Supplementary material for: Virtual Patient Simulations in Health Professions Education: Systematic Review and Meta-Analysis by the Digital Health Education Collaboration
Source: J Med Internet Res. 2019 Jul 2;21(7):e14676. doi: 10.2196/14676 (PMC6632099; doi:10.2196/14676)
Supplement: Multimedia Appendix 1 [file jmir_v21i7e14676_app1.doc]

# Multimedia Appendix 1: Glossary

| **Feature** | **Description** |
| --- | --- |
| Backstory video | A feature in virtual patient simulations (e.g. [1]) in which the learner follows a video clip with computer-generated characters role-playing scenes illustrating the virtual patients’ conditions e.g. to increase the learner’s empathy or provide feedback. |
| Branched navigation | Type of virtual patient system navigation scheme in which the actions of the learner (i.e. selecting from a list or menu of response options) will determine how the virtual patient scenario will unfold in the next screen. In a true branched virtual patient the case will have alternate endings determined by the learner’s actions. |
| Campus-based access | Type of access to educational content which requires learners to use virtual patient software being physically present in a classroom at academic institution’s premises, which is often conducted with a face-to-face instructor-led learning activity. |
| Consequential feedback | See Intrinsic feedback |
| Continuing Medical Education | By CME, we mean *“all educational activities which serve to maintain, develop, or increase the knowledge, skills, and professional performance and relationships that a physician uses to provide services for patients, the public, or the profession”* [2]. |
| Continuing Professional Development | CPD is defined as *“a range of learning activities through which health and care professionals maintain and develop throughout their career to ensure that they retain their capacity to practice safely, effectively and legally within their evolving scope of practice”* [3]. |
| Direct feedback | See explicit feedback |
| Dynamic physiology engine | A mathematical model to simulate some dynamic (time-dependent) often physiological processes in the human body. The learner is allowed to control some input variables of the model to experience realistic, real-time consequences of performed changes. |
| Empathy feedback | A Mechanical Turk mechanism (see term in this table) used e.g. in [1] to improve students’ empathic communication in virtual patient simulation. |
| Explicit feedback | A definitive response of the virtual patient system to students’ actions to inform whether the decision made was correct or not. Often includes an explanation why the option was correct of incorrect (i.e. instructional feedback). |
| Free access navigation | See Problem-solving approach |
| Free text interaction | A type of virtual patient system user interface which allows the learner to engage in a natural language dialogue with the virtual patient by typing in questions and seeing responses of the virtual patient in a text chat mode. |
| High-fidelity format | Fidelity in medical simulation is often understood as the degree to which a simulation looks, feels, and acts like a real human patient [4]. The multifactorial nature of the term makes the perceived level of fidelity context specific and difficult to define. In the papers included in this review high fidelity was understood by Friedman et al. [5] as the possibility to ask questions to the virtual patient in natural language and by constraining feedback to informing about clinical consequences of decisions only. This was contrasted with low fidelity in the pedagogic format which involved selection of options via menus and rich instructional feedback. Dankbaar et al. [6] represents higher fidelity by adding to a virtual patient simulation a dynamic physiology engine, a multimedia user interface, more realistic feedback and tasks which were either not present or less realistic in low-fidelity text-based cases. |
| Home access | Students interact with virtual patient simulations outside the campus using their own computer or mobile devices. |
| Curriculum integration | Virtual patient simulation is a mandatory or explicitly recommended part of a course in the official curriculum of an academic institution. |
| Intrinsic feedback | Type of feedback in which instead of informing the learner directly whether the decision was correct or not, the response of the system shows real-world consequences of learner’s actions, allowing the learner to infer whether or not the action was correct. |
| Laboratory study | The effectiveness of the virtual patient intervention was evaluated in an experiment designed specifically for research purposes and independent of the curriculum of an academic institution. |
| Linear navigation | Type of virtual patient system navigation scheme in the narrative approach to virtual patients when the learner receives instant feedback on the decisions or choices made, which in turn do not influence the way the case unfolds. Compare to branched navigation. |
| Mastery learning | A type of instructional design that requires the learner to repeat an assignment until a specified level of performance or competence is reached. |
| Mechanical Turk feedback | A type of anonymous feedback mechanism (e.g. [7]) that allows a human operator to follow a learner’s interaction with the virtual patient in order to provide instant feedback on learner actions which would be difficult or impossible to achieve automatically by the virtual patient system due to technical limitations (e.g. feedback related to human feelings or interpretations of empathy). |
| Menu-based interaction | Type of virtual patient system user interface which allows the learner to interact with the virtual patient by selecting choices e.g. questions in history taking, laboratory tests or in making diagnoses from a list of options. This can be contrasted with free text interaction or speech-recognition which is more difficult to implement technically, but is less likely to cause a cueing effect. |
| Narrative approach | A virtual patient design type which involves presenting the case as a series of interactions over time with a coherent storyline [8]. Can be further differentiated into linear or branched navigation. |
| Pedagogic format | A design model of virtual patients proposed by Friedman et al. [5] that uses menu-based interaction and explicit cues to help students master the content of the case. This model is contrasted with the high-fidelity design with less guidance and interaction with the virtual patient in natural language. |
| Post-registered education | A post-registration health professional educational programme is defined as any type of study after a qualification that is recognised by relevant governmental or professional bodies that enables the qualification holder entry into or continuation of work in the healthcare workforce in the same or a more independent or senior role. This definition includes continuing medical education (CME) and continuing professional development (CPD) programmes that use virtual patients. |
| Pre-registration education | We will follow the definition of pre-registration education or basic vocational training as stated in George et al. [9] : “*any type of study leading to a qualification that: (i) is recognised by the relevant governmental or professional bodies of the country where the studies were conducted; and (ii) entitles the qualification-holder to apply for entry level positions in the healthcare workforce*”. |
| Problem-solving approach | A type of virtual patient design [8] with a wide range of options (e.g. in history taking, wide range of laboratory tests) grouped in menus and submenus, which can be selected in any order. Learners decide how many interactions with the virtual patient they have until entering a final response (e.g. diagnosis or treatment recommendation), which is not controlled by a linear or branched navigation pathway. |
| Representation scaffolding | Representation is a "statement of the [virtual patient] case as far as it is summarised in the mind" [10]. A scaffolding exercise is an instructional method that helps students to handle a problem they could not solve without support. In the study by Braun et al. [10] representation scaffolding involved answering an additional question "Please sum up the case as you would present it to your attending expert" displayed during the virtual patient learning activity. |
| Self-determined use of virtual patients | The virtual patient simulation is recommended by teachers in a learning activity, but the timing and number of completed cases is selected arbitrarily by students. This approach to integration of virtual patients into the curriculum is contrasted with mandatory use of virtual patients which involve rigorous checks of some activity patterns in students’ learning (as investigated e.g. in [11]). |
| Spaced activation | Presentation of new virtual patient cases in a timed order. After a fixed time interval, a new batch of cases is released to the learner. This is different from continuous access in which all virtual patient cases are available from the beginning of the course (evaluated e.g. in [12]). |
| Speech-recognition interaction | The audio utterances of learners are recorded and processed by a speech recognition engine, then interpreted whereby the system gives a voice response in order to enable a natural conversation with the virtual patient (as e.g. in [13]). |
| Traditional teaching | Learning activities that do not use information technologies as the main modality for presenting educational content or interacting with students. This includes lectures on campus, classroom-based and teacher-led lessons and hospital rounds. We do not regard mere use of electronic formats (e.g. PowerPoint, PDF) presented in a classroom or lecture hall by a human teacher as e-Learning but categorise these as traditional teaching |
| Usability enhancements | Changes in the user interface of a virtual patient simulation in order to make it more user friendly and easy to navigate. The influence of such changes on learning effects in virtual patients was investigated e.g. in [14]. |
| Worked case | A demonstration of how a virtual patient scenario should be completed. Includes many commentaries on how to proceed or why decisions are made and may be contrasted with an unguided type of interaction with virtual patients (e.g. in [15]). |

# References

1. Foster A, Chaudhary N, Kim T, Waller JL, Wong J, Borish M, et al. Using Virtual Patients to Teach Empathy: A Randomized Controlled Study to Enhance Medical Students’ Empathic Communication. Simul Healthc 2016 Jun;11(3):181–9. PMID: 26841278

2. ACCME. CME Content: Definition and Examples. Online [Internet]. Accredit Counc Contin Med Educ. 2015. Available from: http://www.accme.org/accreditation-rules/policies/cme-content-definition-and-examples

3. HCPC. Continuing professional development (CPD) [Internet]. Heal Care Prof Counc. 2015. Available from: http://www.hcpc-uk.org/registrants/cpd

4. Hamstra SJ, Brydges R, Hatala R, Zendejas B, Cook DA. Reconsidering fidelity in simulation-based training. Acad Med 2014 Mar;89(3):387–92. PMID: 24448038

5. Friedman CP, France CL, Drossman DD. A randomized comparison of alternative formats for clinical simulations. Med Decis Making 1991;11(4):265–72. PMID: 1766329

6. Dankbaar MEW, Alsma J, Jansen EEH, van Merrienboer JJG, van Saase JLCM, Schuit SCE. An experimental study on the effects of a simulation game on students’ clinical cognitive skills and motivation. Adv Health Sci Educ Theory Pract Springer Netherlands; 2016 Aug;21(3):505–21. PMID: 26433730

7. Foster A, Chaudhary N, Murphy J, Lok B, Waller J, Buckley PF. The Use of Simulation to Teach Suicide Risk Assessment to Health Profession Trainees-Rationale, Methodology, and a Proof of Concept Demonstration with a Virtual Patient. Acad Psychiatry 2015 Dec;39(6):620–9. PMID: 25026950

8. Bearman M, Cesnik B, Liddell M. Random comparison of “virtual patient” models in the context of teaching clinical communication skills. Med Educ 2001 Sep;35(9):824–32. PMID: 11555219

9. George PP, Papachristou N, Belisario JM, Wang W, Wark PA, Cotic Z, et al. Online eLearning for undergraduates in health professions: A systematic review of the impact on knowledge, skills, attitudes and satisfaction. J Glob Health 2014 Jun;4(1):010406. PMID: 24976965

10. Braun LT, Zottmann JM, Adolf C, Lottspeich C, Then C, Wirth S, et al. Representation scaffolds improve diagnostic efficiency in medical students. Med Educ 2017 Nov;51(11):1118–1126. PMID: 28585351

11. Mahnken AH, Baumann M, Meister M, Schmitt V, Fischer MR. Blended learning in radiology: is self-determined learning really more effective? Eur J Radiol 2011 Jun;78(3):384–7. PMID: 21288674

12. Maier EM, Hege I, Muntau AC, Huber J, Fischer MR. What are effects of a spaced activation of virtual patients in a pediatric course? BMC Med Educ 2013 Mar 28;13(1):45. PMID: 23537162

13. Deladisma AM, Gupta M, Kotranza A, Bittner JG, Imam T, Swinson D, et al. A pilot study to integrate an immersive virtual patient with a breast complaint and breast examination simulator into a surgery clerkship. Am J Surg Elsevier Inc.; 2009 Jan;197(1):102–6. PMID: 19101251

14. Davids MR, Chikte UME, Halperin ML. Effect of improving the usability of an e-learning resource: a randomized trial. Adv Physiol Educ 2014 Jun;38(2):155–60. PMID: 24913451

15. Harris JM, Sun H. A randomized trial of two e-learning strategies for teaching substance abuse management skills to physicians. Acad Med 2013 Sep;88(9):1357–62. PMID: 23887001
