# Supplementary material for: Virtual Patient Simulations in Health Professions Education: Systematic Review and Meta-Analysis by the Digital Health Education Collaboration
Source: J Med Internet Res. 2019 Jul 2;21(7):e14676. doi: 10.2196/14676 (PMC6632099; doi:10.2196/14676)
Supplement: Multimedia Appendix 3 [file jmir_v21i7e14676_app3.doc]

# Multimedia Appendix 3: Summary of included studies

Table 1 General characteristics of included studies

| **Study** | **Cp** | **Design** | **S.Arms** | **Control** | **N** | **Field** | **Stage** | **Year** | **Econ** | **Country** | **Outcomes** |
| --- | --- | --- | --- | --- | --- | --- | --- | --- | --- | --- | --- |
| Al-Dahir 2014 | 1.T | RCT | 2 | Group | 119 | Ph | Pre | 4 | H | USA | K, Sf |
| Bonnetain 2010 | 1.T | RCT | 2 | Mix | 28 | M | Pre | 2 | H | France | Sk |
| Botezatu 2010 | 1.T | RCT | 2 | Mix | 49 | M | Pre | 4 | LM | Colombia | Sk |
| Botezatu 2010a | 1.T | RCT | 2 | Mix | 106 | M | Pre | 4 | LM | Colombia | Sk |
| Fleetwood 2000 | 1.T | RCT | 2 | Mix | 172 | M | Pre | 2 | H | USA | K, Sk, A |
| Haerling 2018 | 1.T | RCT | 2 | Man | 84 | N | Pre | 2 | H | USA | K, Sf, C |
| Jeimy 2018 | 1.T | RCT | 2 | Read | 52 | M | Mix | - | H | Canada | K, A, Sf |
| Kandasamy 2009 | 1.T | RCT | 2 | Read | 62 | M | Pre | 2 | H | Canada | K |
| Kinney 1997 | 1.T | RCT | 2 | Mix | 10 | PT | Pre | 3 | H | USA | K |
| Leong 2003 | 1.T | RCT | 3 | Read | 54 | M | Pre | 2-3 | H | USA | K, Sf |
| Li 2013 | 1.T | RCT | 4 | Lect | 120 | M | Pre | 4 | LM | China | K, Sk, Sf |
| Liaw 2014 | 1.T | RCT | 2 | Man | 57 | N | Pre | 3 | H | Singapore | Sk |
| Maleck 2001 | 1.T | RCT | 4 | Group | 192 | M | Pre | 3 | H | Germany | K, Sk, Sf |
| Miedzybrodzka 2001 | 1.T | RCT | 2 | Lect | 48 | M | Pre | 4 | H | UK | K, Sf |
| Qayumi 2004 | 1.T | RCT | 4 | Read | 99 | M | Pre | 3 | H | Japan | K, Sk, Sf |
| Schwid 1999 | 1.T | RCT | 2 | Read | 45 | M | Post | - | H | USA | Sk |
| Schwid 2001 | 1.T | RCT | 2 | Read | 31 | M | Post | 1 | H | USA | Sk |
| Secomb 2012 | 1.T | RCT | 2 | Man | 28 | N | Pre | 3 | H | Australia | K |
| Sobocan 2017 | 1.T | RCT | 2 | Group | 34 | M | Pre | 3 | H | Slovenia | K |
| Subramanian 2012 | 1.T | RCT | 2 | Lect | 33 | M | Pre | 3 | H | USA | K |
| Tao 2011 | 1.T | RCT | 2 | Group | 92 | N | Pre | ? | LM | China | K, Sk |
| Triola 2006 | 1.T | RCT | 2 | SP | 55 | I | Post | - | H | USA | Sk, A, Sf |
| Vash 2007 | 1.T | RCT | 2 | Ward | 48 | M | Pre | 4 | LM | Iran | K, Sk |
| Wang 2017 | 1.T | RCT | 2 | Man | 40 | I | Post | - | H | USA | K, A |
| Williams 2001 | 1.T | RCT | 2 | Lect | 166 | M | Pre | 4 | H | UK | K, Sk, A |
| Bryant 2015 | 2.B | RCT | 2 | Trad. | 60 | N | Post | - | H | USA | Sk |
| Deladisma 2009 | 2.B | RCT | 2 | Trad. | 21 | M | Pre | 3 | H | USA | A |
| Gu 2017 | 2.B | RCT | 2 | Trad. | 28 | N | Pre | 2 | LM | China | K, Sk |
| Kaltman 2018 | 2.B | RCT | 2 | Trad. | 99 | M | Pre | 1 | H | USA | Sk, A |
| Kononowicz 2012 | 2.B | cRCT | 2 | Trad. | 159 | M | Pre | 1 | H | Poland | K, Sk |
| Lehmann 2015 | 2.B | RCT | 2 | Trad. | 57 | M | Pre | 3-4 | H | Germany | K, Sk, A |
| Schittek 2004 | 2.B | RCT | 2 | Trad. | 39 | D | Pre | 1 | H | Sweden | Sk |
| Smith 2011 | 2.B | RCT | 2 | Trad. | 199 | OM | Pre | 2 | H | USA | A |
| Succar 2013 | 2.B | cRCT | 2 | Trad. | 188 | M | Pre | 3 | H | Australia | K |
| Wahlgren 2006 | 2.B | RCT | 2 | Trad. | 116 | M | Pre | 4 | H | Sweden | K |
| Weverling 1996 | 2.B | RCT | 2 | Trad. | 103 | M | Pre | - | H | The Netherlands | Sk |
| Courteille 2018 | 3.D | RCT | 2 | Video | 170 | M | Mix | - | H | Sweden | K, Sf |
| Dankbaar 2016 | 3.D | RCT | 3 | Tutorial | 79 | M | Pre | 4 | H | The Netherlands | Sk, Sf |
| Foster 2015 | 3.D | RCT | 2 | Video | 67 | M | Pre | 2 | H | USA | Sk, Sf |
| Kumta 2003 | 3.D | cRCT | 2 | Mix | 163 | M | Pre | 6 | H | Hong Kong | Sk |
| Trudeau 2017 | 3.D | RCT | 2 | Tutorial | 238 | I | Post | - | H | USA | K, A |
| Bearman 2001 | 4.V | RCT | 3 | V8 | 157 | M | Pre | 3 | H | Australia | Sk |
| Berger 2017 | 4.V | RCT | 2 | V9 | 117 | Ph | Pre | 5 | H | Belgium/Switzerland | K, A, Sf |
| Braun 2017 | 4.V | RCT | 2 | V10 | 100 | M | Pre | 4-5 | H | Germany | K, Sk |
| Davids 2014 | 4.V | RCT | 2 | V1 | 54 | M | Post | - | LM | South Africa | K, Sf |
| Foster 2016 | 4.V | RCT | 3 | V7 | 70 | M | Pre | 1 | H | USA | Sk |
| Friedman 1991 | 4.V | RCT | 3 | V2 | 80 | M | Pre | 3 | H | USA | K, Sf |
| Harris 2013 | 4.V | RCT | 3 | V3 | 170 | M | Post | 1-3 | H | USA | K |
| Mahnken 2011 | 4.V | RCT | 3 | V4 | 96 | M | Pre | 4 | H | Germany | K |
| Maier 2013 | 4.V | RCT | 2 | V5 | 197 | M | Pre | 4 | H | Germany | K |
| Tolsgaard 2016 | 4.V | RCT | 2 | V6 | 45 | M | Pre | 4 | H | Denmark | K, Sk, Sf |

**Cp** (Type of comparison): 1.T=Virtual patient vs traditional; 2.B=Virtual patient blended learning vs traditional education; 3.C=Virtual patient vs other types of digital education; 4.V=Virtual patient design comparison.
**Design** (Study design): RCT=Randomised Controlled Trial; cRCT=Cluster Randomised Controlled Trial.
**S.Arms** (Number of study arms):1-4.
**Control** (Intervention in control group): Read=Reading assignment; Group=Group (collaborative) activity; Lect=Lecture; Man=Mannequin; Ward=Bedside teaching; Paper C.=Paper-based case; Trad=Traditional (when virtual patient is blended with traditional education); SP=Standardised patient; Mix=Mix of methods (e.g. lecture followed by small group exercise with mannequin); Tutorial=Web-tutorial, e-module or on-line course; Video=Video-based module; V1=Addition of usability enhancements; V2=Pedagogic (menus, guided) vs High-fidelity (free text, unguided) format; V3=Worked vs unworked cases; V4=Self-determined vs mandatory use; V5=Spaced vs non-spaced activation; V6=Solving vs constructing virtual patients; V7=No feedback vs emphatic feedback; V8=Narrative vs problem-solving approach; V9=Linear vs Branched; V10=Addition of scaffolding; A glossary of terms is available in Multimedia Appendix 1.
**N** (Number of participants in the study, total).
**Field** (Field of study): M=Medicine; N=Nursing; PT=Physical therapy; OM=Osteopathic Medicine; D=Dentistry; Ph=Pharmacy; OT=Occupational Therapy; I=Interprofessional education.
**Stage** (Stage of education): Pre=Pre-registered; Post=Post-registered.
**Year** (Student’s year of study): 1-6.
**Econ** (World Bank Income Category): H=High Income Country; LM=Low&Middle Income Country.
**Country** (Place where the study was conducted).
**Outcomes** (outcomes measured): K=Knowledge; Sk=Skills; A=Attitudes; Sf=Satisfaction; C=Cost-Effectiveness.

Table 2 Characteristics of studies assessing knowledge

| **Study** | **Cp** | **ST** | **AM** | **V** | **Control** | **M1** | **SD1** | **n1** | **M2** | **SD2** | **n2** | **SMD** | **Sum** |
| --- | --- | --- | --- | --- | --- | --- | --- | --- | --- | --- | --- | --- | --- |
| Al-Dahir 2014 | 1.T | K | MCQ | Y | Group | 74.80 | 11.70 | 60 | 66.50 | 13.60 | 59 | -0.65 | ↓ |
| Fleetwood 2000 | 1.T | K | MCQ | N | Mix | 0.83 | 0.05 | 88 | 0.83 | 0.05 | 84 | 0.00 | ↔ |
| Haerling 2018 | 1.T | K | MCQ | N | Man | 79.82 | 17.63 | 44 | 82.16 | 11.76 | 37 | 0.15 | ↔ |
| Jeimy 2018 | 1.T | K | MCQ | N | Read | - | - | 29 | - | - | 23 | - | ↔ |
| Kandasamy 2009 | 1.T | K | MCQ | N | Read | 0.74 | 0.15 | 27 | 0.85 | 0.13 | 28 | 0.74 | ↑ |
| Kinney 1997 | 1.T | K | MCQ | Y | Mix | 28.20 | 2.49 | 5 | 26.40 | 1.82 | 5 | -0.83 | ↔ |
| Leong 2003 | 1.T | K | ? | N | Read | 0.66 | - | 27 | 0.63 | - | 27 | - | ↔ |
| Li 2013 | 1.T | K | Mix | N | Lect | 60.30 | 12.50 | 30 | 71.80 | 10.70 | 30 | 0.99 | ↑ |
| Maleck 2001 | 1.T | K | MCQ | Y | Group | 0.89 | - | 42 | 0.91 | - | 47 | - | ↔ |
| Miedzybrodzka 2001 | 1.T | K | Mix | N | Lect | 30.70 | 5.80 | 32 | 28.90 | 4.80 | 16 | -0.33 | ↔ |
| Qayumi 2004 | 1.T | K | MCQ | Y | Read | 34.66 | 11.97 | 24 | 46.40 | 14.61 | 25 | 0.88 | ↑ |
| Secomb 2012 | 1.T | M | Surv | Y | Man | 370.69 | 51.43 | 15 | 345.55 | 26.00 | 13 | -0.60 | ↔ |
| Sobocan 2017 | 1.T | K | MCQ | N | Group | 69.88 | 10.14 | 17 | 69.25 | 5.61 | 16 | -0.08 | ↔ |
| Subramanian 2012 | 1.T | K | MCQ | N | Lect | 0.62 | 0.02 | 15 | 0.87 | 0.02 | 15 | 12.50 | ↑ |
| Tao 2011 | 1.T | K | ? | N | Group | 82.88 | 10.88 | 46 | 83.88 | 10.21 | 46 | 0.09 | ↔ |
| Vash 2007 | 1.T | K | Txt | Y | Ward | 6.80 | 1.50 | 22 | 7.00 | 1.50 | 23 | 0.13 | ↔ |
| Wang 2017 | 1.T | K | MCQ | N | Man | 0.80 | - | 18 | 0.78 | - | 15 | - | ↔ |
| Williams 2001 | 1.T | K | MCQ | N | Lect | 34.40 | 10.95 | 85 | 33.10 | 10.95 | 78 | -0.12 | ↔ |
| Gu 2017 | 2.B | K | MCQ | N | Trad | 65.36 | 8.93 | 14 | 73.31 | 9.27 | 13 | 0.87 | ↑ |
| Kononowicz 2012 | 2.B | K | TF | Y | Trad | 45.81 | 3.76 | 45 | 47.37 | 3.43 | 51 | 0.43 | ↑ |
| Lehmann 2015 | 2.B | K | KF | Y | Trad | 68.80 | 16.30 | 30 | 92.20 | 4.70 | 27 | 1.91 | ↑ |
| Succar 2013 | 2.B | K | MCQ | N | Trad | 14.80 | 2.20 | 74 | 16.00 | 1.80 | 76 | 0.60 | ↑ |
| Wahlgren 2006 | 2.B | K | Txt | N | Trad | 0.88 | 0.08 | 81 | 0.89 | 0.07 | 28 | 0.17 | ↔ |
| Courteille 2018 | 3.C | K | MCQ | N | Video | - | - | 64 | - | - | 74 | - | ↔ |
| Trudeau 2017 | 3.C | K | MCQ | Y | Tutorial | 160.7 | 19.27 | 104 | 158.26 | 18.98 | 103 | -0.13 | ↔ |
| Berger 2017 | 4.V | K | ? | N | V9 | 6.81 | 1.10 | 41 | 7.07 | 1.50 | 30 | 0.20 | ↔ |
| Braun 2017 | 4.V | K | Mix | Y | V10 | 14.80 | 4.00 | 45 | 15.10 | 3.70 | 43 | 0.08 | ↔ |
| Davids 2014 | 4.V | K | Txt | N | V1 | 7.40 | 3.20 | 27 | 6.60 | 2.50 | 27 | -0.28 | ↔ |
| Friedman 1991 | 4.V | K | MCQ | N | V2 | 13.70 | 2.52 | 26 | 11.60 | 2.22 | 24 | -0.88 | ↑ |
| Harris 2013 | 4.V | K | Mix | Y | V3 | 211.10 | 28.14 | 32 | 202.10 | 26.70 | 38 | -0.33 | ↔ |
| Mahnken 2011 | 4.V | K | MCQ | N | V4 | 0.13 | 0.24 | 32 | 0.15 | 0.20 | 32 | 0.10 | ↔ |
| Maier 2013 | 4.V | K | Txt | N | V5 | 35.50 | 2.80 | 99 | 35.90 | 3.60 | 98 | 0.12 | ↔ |
| Tolsgaard 2016 | 4.V | K | MRQ | Y | V6 | 61.40 | 5.20 | 20 | 62.60 | 5.70 | 19 | 0.22 | ↔ |

**Cp** (Type of comparison): 1.T=Virtual patient vs traditional education; 2.B=Virtual patient blended learning vs traditional education; 3.C=Virtual patient vs other types of digital education; 4.V=Virtual patient design comparison.
**ST** (Subtype of competence): K=Core Knowledge; M=Meta-Knowledge.
**AM** (Assessment method): MCQ=Multiple Choice Questions; MRQ=Multiple Response Questions; TF=True/False; KF=Key Feature; Txt=Freetext answer; Surv=Survey; Mix=Mix of methods; ?=Unclear.
**V** (Was the assessment tool validated?): Y=Yes; N=No or unclear.
**Control** (Intervention in control group): Read=Reading assignment; Group=Group (collaborative) activity; Lect=Lecture; Man=Mannequin; Ward=Bedside teaching; Paper C.=Paper-based case; Trad=Traditional (when virtual patient is blended with traditional education); Mix=Mix of methods (e.g. lecture followed by small group exercise with mannequin); Video=Video-based module; Tutorial=Web-tutorial, e-module or on-line course; V1=Addition of usability enhancements; V2=Pedagogic (menus, guided) vs high-fidelity (free text, unguided) format; V3=Worked vs unworked cases; V4=Self-determined vs mandatory use; V5=Spaced vs non-spaced activation; V6=Solving vs constructing virtual patients; V9=Linear vs Branched; V10=Addition of scaffolding; A glossary of terms is available in additional file 1.
**M1,M2** (Arithmetic mean in control and intervention group); **SD1,SD2** (Standard deviation); **n1,n2** (Number of participants in study group); **SMD** (Standardised Mean Difference);
**Sum** (Summary of results): ↑=Intervention (second) group better; ↔=No significant difference; ↓=Control (first) group better.

**Table 3 Characteristics of studies assessing skills**

| **Study** | **Cp** | **ST** | **AM** | **V** | **Control** | **M1** | **SD1** | **n1** | **M2** | **SD2** | **n2** | **SMD** | **Sum** |
| --- | --- | --- | --- | --- | --- | --- | --- | --- | --- | --- | --- | --- | --- |
| Bonnetain 2010 | 1.T | Proc | Man | Y | Mix | 11.13 | 1.56 | 14 | 16.21 | 2.11 | 14 | 2.74 | ↑ |
| Botezatu 2010 | 1.T | CR | Mix | Y | Mix | 6.28 | 1.22 | 24 | 7.93 | 1.20 | 25 | 1.37 | ↑ |
| Botezatu 2010b | 1.T | CR | Mix | Y | Mix | 2.02 | 1.10 | 51 | 3.96 | 0.89 | 55 | 1.95 | ↑ |
| Fleetwood 2000 | 1.T | Com | SP | Y | Mix | 0.86 | 0.14 | 88 | 0.83 | 0.16 | 84 | -0.20 | ↔ |
| Li 2013 | 1.T | CR | Txt | N | Lect | 20.00 | 9.90 | 30 | 30.70 | 8.80 | 30 | 1.14 | ↑ |
| Liaw 2014 | 1.T | Proc | Man | Y | Man | 33.27 | 7.50 | 26 | 36.65 | 5.59 | 31 | 0.52 | ↔ |
| Maleck 2001 | 1.T | CR | Txt | N | Group | 0.32 | - | 42 | 0.35 | - | 47 | - | ↔ |
| Qayumi 2004 | 1.T | Proc | SP | N | Read | 6.98 | 2.93 | 24 | 8.74 | 2.60 | 25 | 0.64 | ↑ |
| Schwid 1999 | 1.T | Proc | Man | N | Read | 29.20 | 4.90 | 22 | 34.90 | 5.00 | 23 | 1.15 | ↑ |
| Schwid 2001 | 1.T | Proc | Man | N | Read | 43.40 | 5.90 | 15 | 52.60 | 9.90 | 16 | 1.12 | ↑ |
| Tao 2011 | 1.T | Int | Man | N | Group | 79.77 | 11.23 | 46 | 87.09 | 12.56 | 46 | 0.61 | ↑ |
| Triola 2006 | 1.T | CR | Txt | N | SP | - | - | 32 | - | - | 23 | - | ↔ |
| Vash 2007 | 1.T | CR | Txt | Y | Ward | 0.61 | 0.18 | 22 | 0.65 | 0.18 | 23 | 0.22 | ↔ |
| Williams 2001 | 1.T | CR | Txt | N | Lect | 11.00 | 3.22 | 85 | 12.50 | 3.22 | 78 | 0.47 | ↑ |
| Bryant 2015 | 2.B | Int | SP | N | Trad | 51.63 | 14.28 | 38 | 46.96 | 12.11 | 22 | -0.35 | ↔ |
| Gu 2017 | 2.B | Proc | Man | N | Trad | 87.54 | 4.60 | 14 | 90.85 | 4.60 | 13 | 0.72 | ↔ |
| Kaltman 2018 | 2.B | Com | SP | Y | Trad | - | - | 39 | - | - | 60 | - | mix |
| Kononowicz 2012 | 2.B | Proc | Man | Y | Trad | - | - | 75 | - | - | 84 | - | mix |
| Lehmann 2015 | 2.B | Proc | Man | Y | Trad | 54.70 | 20.57 | 30 | 76.40 | 15.64 | 27 | 1.18 | ↑ |
| Schittek 2004 | 2.B | Com | RP | N | Trad | 4.00 | - | 23 | 5.00 | - | 26 | - | ↑ |
| Weverling 1996 | 2.B | CR | Txt | Y | Trad | 6.20 | 1.50 | 51 | 7.50 | 1.40 | 52 | 0.90 | ↑ |
| Dankbaar 2016 | 3.C | Proc | Man | Y | Tutorial | 0.80 | 0.08 | 16 | 0.77 | 0.10 | 25 | -0.32 | ↔ |
| Foster 2015 | 3.C | Com | SP | Y | Video | 0.55 | 0.30 | 34 | 0.64 | 0.34 | 33 | 0.27 | ↔ |
| Kumta 2003 | 3.C | Int | Mix | Y | Mix | 52.89 | 5.60 | 44 | 58.72 | 6.80 | 52 | 0.93 | ↑ |
| Bearman 2001 | 4.V | Com | SP | Y | V8 | 38.80 | 4.80 | 41 | 35.70 | 5.30 | 38 | -0.61 | ↓ |
| Braun 2017 | 4.V | CR | VP | N | V10 | 0.09 | 0.06 | 45 | 0.12 | 0.07 | 43 | 0.46 | ↑ |
| Foster 2016 | 4.V | Com | SP | Y | V7 | 2.27 | 0.21 | 17 | 2.91 | 0.16 | 35 | 3.60 | ↑ |
| Tolsgaard 2016 | 4.V | Int | SP | Y | V6 | 59.10 | 12.80 | 20 | 60.80 | 11.50 | 19 | 0.14 | ↔ |

**Cp** (Type of comparison): 1.T=Virtual patient vs traditional education; 2.B=Virtual patient blended learning vs traditional education; 3.C=Virtual patient vs other types of digital education; 4.V=Virtual patient design comparison.
**ST** (Subtype of competence): CR=Clinical reasoning; Proc=Procedural skills; Com=Communication skills; Int=Integrated performance; Crit=Critical thinking.
**AM** (Assessment method): Man=Mannequin; SP=Standardised patient; VP=Virtual patient; RP=Real patient; Txt=Freetext answer; MCQ=Multiple choice questions; Mix=Mix of methods (e.g. lecture followed by small group exercise with mannequin).
**V** (Was the assessment tool validated?);Y=Yes; N=No or unclear.
**Control** (Intervention in control group): Read=Reading assignment; Group=Group (collaborative) activity; Lect=Lecture; Man=Mannequin; Ward=Bedside teaching; SP=standardised patient; Trad=Traditional (when virtual patient is supplement); Tutorial=Web-tutorial, e-module or on-line course; Video=Video-based module; Mix=Mix of methods (e.g. lecture followed by small group exercise with mannequin); V6=Solving vs constructing virtual patients; V7=No feedback vs emphatic feedback; V8=Narrative vs problem-solving approach. V10=Addition of scaffolding; A glossary of terms is available in additional file 1.
**M1,M2** (Arithmetic mean in control and intervention group); **SD1,SD2** (Standard deviation); **n1,n2** (Number of participants in study group); **SMD** (Standardised Mean Difference).
**Sum** (Summary of results): ↑=Intervention (second) group better; ↔=No significant difference; ↓=Control (first) group better; mix=Outcome compared item by item with mixed result - no aggregation of scores was possible.

**Table 4. Characteristics of studies asse**ssing attitudes

| **SID** | **Cp** | **V** | **Control** | **M1** | **SD1** | **n1** | **M2** | **SD2** | **n2** | **SMD** | **Q-** | **Q=** | **Q+** | **Subject of attitude** |
| --- | --- | --- | --- | --- | --- | --- | --- | --- | --- | --- | --- | --- | --- | --- |
| Fleetwood 2000 | 1.T | N | Mix | - | - | 88 | - | - | 84 | - | 0 | 2 | 1 | Preparedness to deal with ethical, legal, and communication issues |
| Jeimy 2018 | 1.T | N | Read | - | - | 29 | - | - | 23 | - | 0 | 5 | 0 | Confidence in diagnostic and management abilities |
| Triola 2006 | 1.T | N | SP | - | - | 32 | - | - | 23 | - | 0 | 5 | 0 | Comfort, reluctance and preparedness in caring for distress disorders patients |
| Wang 2017 | 1.T | N | Man | # | - | 18 | # | - | 15 | - | - | - | - | Comfort with contrast reaction management and teamwork |
| Williams 2001 | 1.T | N | Lect. | - | - | 85 | - | - | 78 | - | 6 | 2 | 0 | Perceived ability to assess, diagnose and manage anxiety |
| Deladisma 2009 | 2.B | N | Trad | - | - | ? | - | - | ? | - | 0 | 3 | 1 | Confidence in history taking and clinical breast examination |
| Kaltman 2018 | 2.B | Y | Trad | 21.17 | 3.31 | 29 | 22.08 | 2.25 | 39 | 0.33 | - | 1* | - | Communication-Related Self-Efficacy |
| Lehmann 2015 | 2.B | Y | Trad | 59.60 | 15.80 | 30 | 72.30 | 11.70 | 27 | 0.91 | - | - | 1* | Self-assessment of procedural knowledge and skills |
| Smith 2011 | 2.B | Y | Trad | ^ | - | 102 | ^ | - | 97 | - | - | - | - | Clinical Cultural Competence |
| Trudeau 2017 | 3.C | Y | Tutorial | 4.17 | 0.62 | 106 | 4.06 | 0.60 | 99 | -0.18 | - | 1* | - | Attitude about opioid therapy |
| Berger 2017 | 4.V | N | V9 | # | - | 29 | # | - | 41 | - | - | - | - | Intention to perform pharmacy triage. Confidence in knowledge and skills to triage cough |

**Cp** (Type of comparison): 1.T=Virtual patient vs traditional education; 2.B=Virtual patient blended learning vs traditional education; 3.C=Virtual patient vs other types of digital education; 4.V=Virtual patient design comparison.
**V** (Was the questionnaire validated?): Y=Yes; N=No or unclear.
**Control** (Intervention in control group): Trad=Traditional (when virtual patient is supplement); Group=Group (collaborative) activity; Read=Reading assignment; Paper C.=Paper-based case; Lect=Lecture; Man=Mannequin; SP=Standardised patient; Mix=Mix of methods (e.g. lecture followed by small group exercise with mannequin); Tutorial=Web-tutorial, e-module or on-line course; V9=Linear vs Branched .
**M1,M2** (Arithmetic mean in control and intervention group); **SD1,SD2** (Standard deviation); **n1,n2** (Number of participants in study group); **SMD** (Standardised Mean Difference).
**Q-** (Number of items in questionnaire in favour of control (first) group); **Q=** (Number of items in questionnaire without significant difference); **Q+** (Number of items in questionnaire in favour of intervention (second) group)
*) One aggregated score; ^) Results presented separately for subgroups (bilingual and English speaking students) ?) Number of participants in subgroups unclear: #) Compared item-by-item and presented p-values of pre-/post-test comparisons only

Table 5. Characteristics of studies assessing satisfaction

| **SID** | **Cp** | **V** | **Control** | **M1** | **SD1** | **n1** | **M2** | **SD2** | **n2** | **SMD** | **Q-** | **Q=** | **Q+** |
| --- | --- | --- | --- | --- | --- | --- | --- | --- | --- | --- | --- | --- | --- |
| Al-Dahir 2014 | 1.T | Y | Group | - | - | 60 | - | - | 59 | - | 2 | 8 | 1 |
| Haerling 2018 | 1.T | Y | Man | 82.57 | 9.88 | 44 | 78.99 | 10.73 | 37 | -0.35 | - | 1* | - |
| Jeimy 2018 | 1.T | N | Read | - | - | 29 | - | - | 23 | - | 0 | 11 | 0 |
| Leong 2003 | 1.T | N | Read | - | - | 27 | - | - | 27 | - | 0 | 1 | 0 |
| Li 2013 | 1.T | Y | Lect | - | - | 30 | - | - | 30 | - | 0 | 4 | 6 |
| Maleck 2001 | 1.T | N | Paper C. | - | - | 42 | - | - | 47 | - | 1 | 3 | 0 |
| Miedzybrodzka 2001 | 1.T | N | Group | - | - | 32 | - | - | 16 | - | 0 | 4 | 0 |
| Qayumi 2004 | 1.T | N | Read | - | - | 24 | - | - | 25 | - | 0 | 2 | 6 |
| Triola 2006 | 1.T | N | SP | - | - | 32 | - | - | 23 | - | 0 | 1 | 0 |
| Wang 2017 | 1.T | N | Man | - | - | 18 | - | - | 15 | - | 5 | 1 | 0 |
| Courteille 2018 | 3.C | N | Video | ^ | - | 74 | ^ | - | 64 | - | - | - | - |
| Dankbaar 2016 | 3.C | Y | Tutorial | - | - | 16 | - | - | 25 | - | 0 | 0 | 2 |
| Foster 2015 | 3.C | Y | Video | - | - | 34 | - | - | 33 | - | 0 | 1 | 4 |
| Berger 2017 | 4.V | N | V9 | - | - | 41 | - | - | 29 | - | 0 | 12 | 2 |
| Davids 2014 | 4.V | Y | V1 | 76.60 | 18.20 | 27 | 81.50 | 12.90 | 27 | 0.31 | - | 1* | - |
| Friedman 1991 | 4.V | N | V2 | 30.52 | 5.94 | 26 | 37.42 | 6.22 | 24 | 1.14 | - | - | 1* |
| Tolsgaard 2016 | 4.V | N | V6 | 4.10 | 0.60 | 20 | 4.00 | 0.70 | 19 | -0.15 | - | 1* | - |

**Cp** (Type of comparison): 1.T=Virtual patient vs traditional education; 3.C=Virtual patient vs other types of digital education; 4.V= Virtual patient design comparison.
**V** (Was the assessment tool validated?) Y=Yes; N=No or unclear.
Control (Intervention in control group); Read=Reading assignment; Group=Group (collaborative) activity; Lect=Lecture; Man=Mannequin; Paper C.=Paper-based case; SP=Standardised patient; Tutorial=Web-tutorial, e-module or on-line course; Video=Video-based module; V1=Addition of usability enhancements; V2=Pedagogic (menus, guided) vs high-fidelity (free text, unguided) format; V6=Solving vs constructing virtual patients; V9=Linear vs Branched.
**M1,M2** (Arithmetic mean in control and intervention group); **SD1,SD2** (Standard deviation); **n1,n2** (Number of participants in study group); **SMD** (Standardised Mean Difference).
**Q-** (Number of items in questionnaire in favour of control (first) group); **Q=** (Number of items in questionnaire without significant difference); **Q+** (Number of items in questionnaire in favour of intervention (second) group)
*) one aggregated score; ^) Results presented graphically only, without measure of dispersion and separately for subgroups (students/residents)
