# Supplementary material for: Virtual Patient Simulations in Health Professions Education: Systematic Review and Meta-Analysis by the Digital Health Education Collaboration
Source: J Med Internet Res. 2019 Jul 2;21(7):e14676. doi: 10.2196/14676 (PMC6632099; doi:10.2196/14676)
Supplement: Multimedia Appendix 4 [file jmir_v21i7e14676_app4.doc]

# Multimedia Appendix 4: Summary of technical and educational features of included studies

## Technical characteristics of virtual patient simulations

The technical characteristics of virtual patient simulations are summarised in Table 1. A glossary of the terms is presented in Multimedia Appendix 1.

### Virtual patient system

Many of the characteristics depend on the software used to display the virtual patient – i.e. the virtual patient system. The most popular system in the review was CASUS with six studies [1–6], followed by Web-SP with three studies [7–9], Virtual People Factory [10,11], Laerdal vSim [12,13] and Laerdal MicroSim [14,15] with two papers respectively. The remaining 17 explicitly named software systems occurred in the review just once (abcdeSIM; ACLS Simulator 3.11; Anesthesia Simulator 3.0; CAMPUS Card; CASES; CyberActive Technology CyberPatient; DecisionSim/VpSim; Electrolyte Workshop - HandsOn module; e-RAPIDS; MedEthEx Online; NUDOV; Physical Therapy Patient Simulator; Shadow Health Digital Clinical Experience (DCE); StepStone Interactive Medical Software; STS Training Module ('Simulative Training System'); VIPS/DIANA (Digital Animated Avator); Virtual Ophthalmology Clinic (VOC)). Berger et al. [16] designed virtual patients in a serious game authoring tool (ITyStudio) and compared them with patient cases presented in the Moodle learning management system. Kaltman et al. [17] used another general purpose e-learning authoring tool (Articulate Storyline) for developing virtual patients. Seventeen studies did not mention a virtual patient system by name.

### Navigation logic

Virtual patients have different navigation schemes which define the logic relating to how the learner progresses through the case. These schemes and resultant interaction between the user and the system often underpinned a designed pedagogical approach, for example the user must complete a task correctly before proceeding or the learner must not be cued in decisions regarding the work flow. The navigation schemes are divided according to the classification by Bearman et al [18] into problem-solving (free access) and narrative approach which is further differentiated into linear and branched. In almost half of the virtual patient interventions (25 out of 51) navigation followed the problem-solving approach. Five of them provided additionally access to a dynamic physiology simulation [14,19–22]. In 17 out of 51 studies, the case followed the narrative approach: in most (14 of 17) cases the progress was linear, in three studies was the navigation scheme branched [16,17,23]. One of the linear virtual patients included a dynamic physiology simulation [24]. In two studies the aim was to contrast variants of the problem-solving and narrative approach [18,25]. In seven studies the nature of navigational scheme in the virtual patient was unclear [26–32].

### Control elements

The navigation in virtual patients can be technically controlled using various interaction elements: selection of options in menus, keyboard entry or through speech recognition. The main interaction mechanism in 40 out of 51 studies were menus. In four studies the participants controlled the interaction by typing in the question to the virtual patient or requested data items by keyboard that was recognised by a natural language processor [10,11,33,34]. In one study user commands were analysed using a speech recognition engine [35]. Access to content was mixed (menu-based with free-text components) in four studies [25,36–38].

### Feedback delivery

Feedback is a key component in interaction with virtual patients as it provides learners with the opportunity to improve suboptimal performance. In some cases the authors of the reviewed studies decided that the complexity of the feedback process required human augmentation and could not be entirely delegated to computer mechanisms. In 35 out of 51 studies feedback was automatically provided by the virtual patient system and did not require any intervention from the human teacher. In four studies the feedback was provided individually by a human instructor - either by e-mail [22,34,39] or in an on-line discussion and face-to-face meetings [37]. In one study it was a combination of computer and tutor feedback [6]. Foster et al. [11] directly contrasted a personalised, human-based feedback model with an automated feedback model. In six studies the description of the feedback was not detailed enough to make a decision. In two studies it was declared that students did not receive any feedback [1,9].

### Feedback timing

The timing of feedback was mixed: 13 out of 51 studies providing feedback to students’ actions directly during the activity, 18 out of 51 post-activity, in eight cases it was a combination of during and post-activity feedback; we could not make a decision based on the description on the timing of feedback in further ten studies.

### Details of the feedback mechanism

Based on the free-text descriptions of feedback mechanisms we could establish that the feedback was often presented explicitly with direct textual explanation of the expected correct answer [2–4,16,17,19,20,23,24,27,28,36,40–44]. In some studies a score summary was given [12,13,19,20,27,30,38,44] or a number was presented expressing the plausibility (probability) of the answer [25]. In two cases the feedback was in the form of references to other sources of knowledge such as guidelines and review papers [23,45] or other learning modules [23]. Experts solutions were presented as examples for comparison by students rather than suggesting that the given answer is wrong [7,46]. In the following studies checklists were used to summarise the completeness of actions in the activity [12–14,16,20,30,34,36,38,43,44], in one case the list of expected answers was compared for alignment of priorities (i.e. position of a hypothesis in the differential diagnosis between student and expert) [43]. In two studies more complex validity checks were described as analysis of “relevance to working hypothesis of clinical data elicited” [25] and the check of “declarative rules” [14]. Peer student feedback was only mentioned in one study where it was provided in the form of ‘high score list’ to stimulate competition between learners [19].

A type of feedback in which instead of informing the learner directly whether the decision was correct or not, the response of the system showing real-world consequences of learner’s actions (intrinsic feedback, [47]) was rarely described. If present it had the form of changing values in a dynamic mathematical model [19–22], led to alternative branches in narration [16,23,25], presented predicted (or real world) further development of the case [7,8], resulted in transition of the patient between different discrete states [18,19] (e.g. patient moods: happy vs angry) or had the form of visualisations [24,48] (e.g. animations showing consequential system reactions to different instruments used).

## Educational content

Table 2 characterises the educational content of the virtual patient intervention, context of use and intensity (time on the task) of the intervention. In thirteen studies the content of virtual patients was a mix of topics from internal medicine [1,6–9,25–28,31,38,45,48], in eight studies the topic of cases was around life support and critical care [2,13,14,19,20,30,42,49], in five studies the problems covered by virtual patients were of psychiatric or psychosocial nature [10,11,18,43,50]. In three studies virtual patients were used to present topics from Radiology [3,5,32]. Two studies respectively covered the topics of Dermatology [29,46], Cardiology [15,23], Anaesthesia [21,22], Surgery [35,37], Nursing [12,33], Communication Skills [17,41]. The following topics were represented by a single study each: Bioethics, Dentistry, Genetics, Neurology, Ophthalmology, Orthopaedics, Paediatrics, Pharmacy Triage, Physiology, Physiotherapy.

In 31 of 51 studies the language of virtual patient content was English, in five studies the language was German, in two Spanish and Dutch. The remaining languages in use where Danish, French, Japanese, Polish and Swedish. In six studies the language of virtual patient was unclear. Only in three studies was the language of virtual patient content anything other than the official language of the country where the study was conducted [6,12,38]. In 38 out of 51 studies the content of virtual patients was developed at the institution where the study was conducted (Table 2). In nine studies the content was external (e.g. included in a commercial package or acquired thanks to a collaboration between institutions): [6,12–15,21,22,33,48]. In four studies the source of the content was unclear [29,30,42,44].

The number of virtual patient cases used in the intervention ranged from single virtual patients presented in 21 studies to 36 cases in [30]. The median number of virtual patient cases presented in the intervention was two, the average number was close to 5. In one study the number of presented virtual patients was unclear [21].

## Setting of use

In countries other than USA and Canada, virtual patients were introduced in the undergraduate medical programme most often in the fourth year of study: 11 studies [3,4,7–9,19,29,38,46,50,51]; followed by seven studies in year three [5,6,15,18,20,39,48]. The study by Lehmann et al. [49] involved both third and fourth year students; the study by Braun both fourth and fifth year students [1]. There were two studies respectively in the first [2,34] and second year [12,14] and one in the fifth [16] and sixth year [37]. In two studies the specific year was unclear [30,44]. In the USA and Canada (treated separately because of the division in baccalaureate pre-med education in years 1-4 and undergraduate education in the 5-8-year medical program) virtual patients were introduced in the first year of medical programme twice [11,17], five times in the second year [10,13,36,41,45], four times in the third year [25,35,42,52] and the fourth year of PharmD programme in one study [23]. The study by Leong et al. [28] involved students both from second and third year. We classify studies in which participated residents [21,22,24,27] as post-registered education.

In most of the studies (40 of 51) students worked on the virtual patients individually. In five studies interacting with virtual patients was a group assignment; in three cases a small group assignment with 2-4 students [5,38,50], in the remaining two a group of 8-10 students worked collaboratively in a PBL setting on solving the case [6,29]. In the study by Kumta et al [37] the intervention was a mixture of individual and group discussion around the virtual patient cases. In five studies it was not clear from the report whether the students worked individually or collaboratively.

In 35 of 51 studies the intervention was part of a course in a health professions curriculum, in 14 studies it was an extra-curricular research study in a laboratory setting. In two studies the setting of use was unclear based on the description (Table 2). Participants used the virtual patients on school premises as classroom or computer labs in 26 of 51 studies. In 12 studies the participants used virtual patients from home. In the study by Fleetwood et al. [36] access was both from home and computer lab. In the rest of the studies the environment in which virtual patients was used was unclear. The virtual patients in the intervention were available in a timeframe ranging from one day up to 24 weeks [27] with a median of 3 weeks. The time spent on the virtual patient assignment ranged from 9 minutes [45] to 14 hours [38]. The median time of use was 95 minutes. We did not have information on the time on the assignment in 12 out of 51 studies.

## Validity of outcome measurement

We classified the type of validity evidence of outcome measurements using a framework introduced by Cook et al. [53].

### Knowledge

In 12 out of 33 studies with knowledge outcomes the authors provided data on the validity evidence of the primary outcome measurement instruments [1,2,5,9,15,23,27,31,38,48,49,52].

In eight cases it was content validity [2,5,15,27,38,48,49,52]. This included expert panels, pilot testing, use of clinical guidelines and items from previously validated instruments. Seven studies provided data on internal structure validity [1,9,15,23,27,31,48]. Validation methods reported in this category included internal test consistency and item analysis. Two studies [27,31] presented validity evidence on relation to other variables (i.e. general training level and physician behaviours).

With the exception of two studies [27,31] the knowledge tests were not published separately as independent instruments, but were created for the purpose of the study or the course in which the tool was implemented. The stand-alone instrument used in [27] was P-CSA: Physicians Competence in Substance Abuse Test [54]. Trudeau et al [31] used in their study a tool for assessing physician pain management education called KnowPain-50 [55]. Additionally, in [6] the DTI: Diagnostic thinking inventory was used [56], yet because of the character of the instrument: test of meta-knowledge on the clinical reasoning process, which was very different from the rest of the instruments in this category, in the presence of a typical knowledge test for this study, we have not selected this outcome as the primary one for the meta-analysis.

### Skills

In 16 out of 28 studies with skills outcomes, the authors provided data on the validity evidence of the outcome measurement instrument. This involved expert panels, use of clinical guidelines as templates and use of items from previously validated instruments. Thirteen studies provided information on internal structure validity, which was presented by data on inter-rater reliability, internal consistency, factor analysis and inter-case reliability. Two studies [9,20] provided information about the validity of the instrument based on relation to other variables (i.e. general training level or concurrent measure). Tolsgaard et al. [9] noted that the instrument had response process validity, but it was not clear from the manuscript how it was verified.

The included studies used established or separately published and verified instruments: MISPQ (Medical Student Interviewing Performance Questionnaire [57]) in [10,11]; ECCS (Empathic Communication Coding System [58]) in [11]; RAPIDS tool [59] in [20], RIME framework [60] in [9]. IPP (Integrated Performance Proficiency) in [33], but we were unable to trace back its validity evidence. In the study by Kononowicz et al [2] the skills evaluation was based on the validated Cardiff Test 3.1 [61], however, because the designer of the instrument regarded all items in the checklist as separate variables, we did not aggregate it into one score and excluded as outcome from the meta-analysis. Fleetwood et al. [36] derived one of their instruments from the National Board of Medical Examiners Patient Satisfaction Scale, but this outcome was not selected as the main outcome for skills and their references are inaccessible. Excluded from analysis were skills outcomes reported by Haerling ([13]) as these were reported for a subgroup only, measured using Lasater Clinical Judgment Rubric (LCJR) ([62]) and Creighton Simulation Evaluation Instrument (C-SEI) [63].

### Attitudes

In all cases these outcomes were measured by self-assessment surveys. One previously validated tool were applied CCCQ (Clinical Cultural Competence Questionnaire) in Smith et al. [41]. Trudeau et al. [31] developed and validated a new tool, PPBS (The Pain Practice Behaviors Scale) for the purpose of their study. Content validity of the questionnaire in Lehmann et al. [49] was verified by an expert panel. Kaltman et al. [17] adapted a previously published questionnaire and tested its internal consistency.

### Satisfaction

Three studies validated their questionnaires by reporting evidence of internal consistency [10,13,23], two further studies were based on previously validated tools, but we were unable to establish the details of the validation process [19,29]. Davids et al. [24] applied a well-established usability tool: SUS (System Usability Scale, [64]).

## Use of educational theory

### Theories in relation to design of virtual patients or the intervention

Few (n=11) of the included studies referred to educational theories when designing virtual patients or the intervention. This could be explained by the virtual patient software and virtual patient cases being developed by others preceding the intervention. No more than two studies used the same theoretical perspective. The theories used for designing virtual patients or the intervention were: Schon's Reflection-In-Action [18]; Cognitive Load Theory [19,24]; Adult Learning Theory [17]; Mayer's Cognitive Theory of Multimedia [2,24]; Kolb's Experiential Learning Theory [20]; NLN Jeffries Simulation Theory [13]; Self Determination Theory [3]; Scaffolding effect [1]; Spacing Effect [4]; Situated Learning Theory [33].

### Theories in relation to interpretations of outcomes of the intervention

Given that an aim of all the interventions was to increase learning, it was surprising to find that only 9 of the included studies refer to learning theories in interpreting outcomes of the educational intervention. Only three theoretical concepts were used in more than one study to discuss results (Cognitive Load Theory, Expertise Reversal Effect, Constructivist Learning Theory), each used in two studies, respectively. List of theories used to interpret outcomes from interventions: Cognitive Load Theory [19,24]; Kalyuga’s Expertise Reversal Effect [9,19]; Social Constructivism Theory [23]; Mayer's Cognitive Theory of Multimedia [24]; NLN Jeffries Simulation Theory [13]; Spacing effect [4]; Perry's Schema of Cognitive and Ethical Development [15]; Situated Learning Theory [33]; Theory of Planned Behaviour [16].

## Unit of analysis issues

Three studies included in the review were conducted following a cluster RCT design. Two of them were studies evaluating virtual patients as an addition to traditional education [2,39], in one study the use of virtual patient was compared to a mix of learning activities including access to lecture materials and videos on-line [37]. The number of clusters ranged from 11 in [37], 16 in [2], to 32 in [39]. The number of participants in each cluster ranged from 6 in [39], to 12-15 in [2] and 14-15 in [37]. Each of the studies analysed data with individual students as the unit of analysis and no one reported any additional methods to correct this bias. Because none of the studies reported intra-cluster correlation coefficients (ICC) we used the estimate ICC=0.05 from the Cochrane Handbook to inflate variances by calculating effective sample size to cover the unit of analysis error ([65], p. 496).

Table 1 General characteristics of included studies

| **Study** | **Cp** | **Design** | **S.Arms** | **Control** | **N** | **Field** | **Stage** | **Year** | **Econ** | **Country** | **Outcomes** |
| --- | --- | --- | --- | --- | --- | --- | --- | --- | --- | --- | --- |
| Al-Dahir 2014 | 1.T | RCT | 2 | Group | 119 | Ph | Pre | 4 | H | USA | K, Sf |
| Bonnetain 2010 | 1.T | RCT | 2 | Mix | 28 | M | Pre | 2 | H | France | Sk |
| Botezatu 2010 | 1.T | RCT | 2 | Mix | 49 | M | Pre | 4 | LM | Colombia | Sk |
| Botezatu 2010a | 1.T | RCT | 2 | Mix | 106 | M | Pre | 4 | LM | Colombia | Sk |
| Fleetwood 2000 | 1.T | RCT | 2 | Mix | 172 | M | Pre | 2 | H | USA | K, Sk, A |
| Haerling 2018 | 1.T | RCT | 2 | Man | 84 | N | Pre | 2 | H | USA | K, Sf, C |
| Jeimy 2018 | 1.T | RCT | 2 | Read | 52 | M | Mix | - | H | Canada | K, A, Sf |
| Kandasamy 2009 | 1.T | RCT | 2 | Read | 62 | M | Pre | 2 | H | Canada | K |
| Kinney 1997 | 1.T | RCT | 2 | Mix | 10 | PT | Pre | 3 | H | USA | K |
| Leong 2003 | 1.T | RCT | 3 | Read | 54 | M | Pre | 2-3 | H | USA | K, Sf |
| Li 2013 | 1.T | RCT | 4 | Lect | 120 | M | Pre | 4 | LM | China | K, Sk, Sf |
| Liaw 2014 | 1.T | RCT | 2 | Man | 57 | N | Pre | 3 | H | Singapore | Sk |
| Maleck 2001 | 1.T | RCT | 4 | Group | 192 | M | Pre | 3 | H | Germany | K, Sk, Sf |
| Miedzybrodzka 2001 | 1.T | RCT | 2 | Lect | 48 | M | Pre | 4 | H | UK | K, Sf |
| Qayumi 2004 | 1.T | RCT | 4 | Read | 99 | M | Pre | 3 | H | Japan | K, Sk, Sf |
| Schwid 1999 | 1.T | RCT | 2 | Read | 45 | M | Post | - | H | USA | Sk |
| Schwid 2001 | 1.T | RCT | 2 | Read | 31 | M | Post | 1 | H | USA | Sk |
| Secomb 2012 | 1.T | RCT | 2 | Man | 28 | N | Pre | 3 | H | Australia | K |
| Sobocan 2017 | 1.T | RCT | 2 | Group | 34 | M | Pre | 3 | H | Slovenia | K |
| Subramanian 2012 | 1.T | RCT | 2 | Lect | 33 | M | Pre | 3 | H | USA | K |
| Tao 2011 | 1.T | RCT | 2 | Group | 92 | N | Pre | ? | LM | China | K, Sk |
| Triola 2006 | 1.T | RCT | 2 | SP | 55 | I | Post | - | H | USA | Sk, A, Sf |
| Vash 2007 | 1.T | RCT | 2 | Ward | 48 | M | Pre | 4 | LM | Iran | K, Sk |
| Wang 2017 | 1.T | RCT | 2 | Man | 40 | I | Post | - | H | USA | K, A |
| Williams 2001 | 1.T | RCT | 2 | Lect | 166 | M | Pre | 4 | H | UK | K, Sk, A |
| Bryant 2015 | 2.B | RCT | 2 | Trad. | 60 | N | Post | - | H | USA | Sk |
| Deladisma 2009 | 2.B | RCT | 2 | Trad. | 21 | M | Pre | 3 | H | USA | A |
| Gu 2017 | 2.B | RCT | 2 | Trad. | 28 | N | Pre | 2 | LM | China | K, Sk |
| Kaltman 2018 | 2.B | RCT | 2 | Trad. | 99 | M | Pre | 1 | H | USA | Sk, A |
| Kononowicz 2012 | 2.B | cRCT | 2 | Trad. | 159 | M | Pre | 1 | H | Poland | K, Sk |
| Lehmann 2015 | 2.B | RCT | 2 | Trad. | 57 | M | Pre | 3-4 | H | Germany | K, Sk, A |
| Schittek 2004 | 2.B | RCT | 2 | Trad. | 39 | D | Pre | 1 | H | Sweden | Sk |
| Smith 2011 | 2.B | RCT | 2 | Trad. | 199 | OM | Pre | 2 | H | USA | A |
| Succar 2013 | 2.B | cRCT | 2 | Trad. | 188 | M | Pre | 3 | H | Australia | K |
| Wahlgren 2006 | 2.B | RCT | 2 | Trad. | 116 | M | Pre | 4 | H | Sweden | K |
| Weverling 1996 | 2.B | RCT | 2 | Trad. | 103 | M | Pre | - | H | The Netherlands | Sk |
| Courteille 2018 | 3.D | RCT | 2 | Video | 170 | M | Mix | - | H | Sweden | K, Sf |
| Dankbaar 2016 | 3.D | RCT | 3 | Tutorial | 79 | M | Pre | 4 | H | The Netherlands | Sk, Sf |
| Foster 2015 | 3.D | RCT | 2 | Video | 67 | M | Pre | 2 | H | USA | Sk, Sf |
| Kumta 2003 | 3.D | cRCT | 2 | Mix | 163 | M | Pre | 6 | H | Hong Kong | Sk |
| Trudeau 2017 | 3.D | RCT | 2 | Tutorial | 238 | I | Post | - | H | USA | K, A |
| Bearman 2001 | 4.V | RCT | 3 | V8 | 157 | M | Pre | 3 | H | Australia | Sk |
| Berger 2017 | 4.V | RCT | 2 | V9 | 117 | Ph | Pre | 5 | H | Belgium/Switzerland | K, A, Sf |
| Braun 2017 | 4.V | RCT | 2 | V10 | 100 | M | Pre | 4-5 | H | Germany | K, Sk |
| Davids 2014 | 4.V | RCT | 2 | V1 | 54 | M | Post | - | LM | South Africa | K, Sf |
| Foster 2016 | 4.V | RCT | 3 | V7 | 70 | M | Pre | 1 | H | USA | Sk |
| Friedman 1991 | 4.V | RCT | 3 | V2 | 80 | M | Pre | 3 | H | USA | K, Sf |
| Harris 2013 | 4.V | RCT | 3 | V3 | 170 | M | Post | 1-3 | H | USA | K |
| Mahnken 2011 | 4.V | RCT | 3 | V4 | 96 | M | Pre | 4 | H | Germany | K |
| Maier 2013 | 4.V | RCT | 2 | V5 | 197 | M | Pre | 4 | H | Germany | K |
| Tolsgaard 2016 | 4.V | RCT | 2 | V6 | 45 | M | Pre | 4 | H | Denmark | K, Sk, Sf |

**Cp** (Type of comparison): 1.T=Virtual patient vs traditional; 2.B=Virtual patient blended learning vs traditional education; 3.C=Virtual patient vs other types of digital education; 4.V=Virtual patient design comparison.
**Design** (Study design): RCT=Randomised Controlled Trial; cRCT=Cluster Randomised Controlled Trial.
**S.Arms** (Number of study arms):1-4.
**Control** (Intervention in control group): Read=Reading assignment; Group=Group (collaborative) activity; Lect=Lecture; Man=Mannequin; Ward=Bedside teaching; Paper C.=Paper-based case; Trad=Traditional (when virtual patient is blended with traditional education); SP=Standardised patient; Mix=Mix of methods (e.g. lecture followed by small group exercise with mannequin); Tutorial=Web-tutorial, e-module or on-line course; Video=Video-based module; V1=Addition of usability enhancements; V2=Pedagogic (menus, guided) vs High-fidelity (free text, unguided) format; V3=Worked vs unworked cases; V4=Self-determined vs mandatory use; V5=Spaced vs non-spaced activation; V6=Solving vs constructing virtual patients; V7=No feedback vs emphatic feedback; V8=Narrative vs problem-solving approach; V9=Linear vs Branched; V10=Addition of scaffolding; A glossary of terms is available in Multimedia Appendix 1.
**N** (Number of participants in the study, total).
**Field** (Field of study): M=Medicine; N=Nursing; PT=Physical therapy; OM=Osteopathic Medicine; D=Dentistry; Ph=Pharmacy; OT=Occupational Therapy; I=Interprofessional education.
**Stage** (Stage of education): Pre=Pre-registered; Post=Post-registered.
**Year** (Student’s year of study): 1-6.
**Econ** (World Bank Income Category): H=High Income Country; LM=Low&Middle Income Country.
**Country** (Place where the study was conducted).
**Outcomes** (outcomes measured): K=Knowledge; Sk=Skills; A=Attitudes; Sf=Satisfaction; C=Cost-Effectiveness.

Table 2 Characteristics of studies assessing knowledge

| **Study** | **Cp** | **ST** | **AM** | **V** | **Control** | **M1** | **SD1** | **n1** | **M2** | **SD2** | **n2** | **SMD** | **Sum** |
| --- | --- | --- | --- | --- | --- | --- | --- | --- | --- | --- | --- | --- | --- |
| Al-Dahir 2014 | 1.T | K | MCQ | Y | Group | 74.80 | 11.70 | 60 | 66.50 | 13.60 | 59 | -0.65 | ↓ |
| Fleetwood 2000 | 1.T | K | MCQ | N | Mix | 0.83 | 0.05 | 88 | 0.83 | 0.05 | 84 | 0.00 | ↔ |
| Haerling 2018 | 1.T | K | MCQ | N | Man | 79.82 | 17.63 | 44 | 82.16 | 11.76 | 37 | 0.15 | ↔ |
| Jeimy 2018 | 1.T | K | MCQ | N | Read | - | - | 29 | - | - | 23 | - | ↔ |
| Kandasamy 2009 | 1.T | K | MCQ | N | Read | 0.74 | 0.15 | 27 | 0.85 | 0.13 | 28 | 0.74 | ↑ |
| Kinney 1997 | 1.T | K | MCQ | Y | Mix | 28.20 | 2.49 | 5 | 26.40 | 1.82 | 5 | -0.83 | ↔ |
| Leong 2003 | 1.T | K | ? | N | Read | 0.66 | - | 27 | 0.63 | - | 27 | - | ↔ |
| Li 2013 | 1.T | K | Mix | N | Lect | 60.30 | 12.50 | 30 | 71.80 | 10.70 | 30 | 0.99 | ↑ |
| Maleck 2001 | 1.T | K | MCQ | Y | Group | 0.89 | - | 42 | 0.91 | - | 47 | - | ↔ |
| Miedzybrodzka 2001 | 1.T | K | Mix | N | Lect | 30.70 | 5.80 | 32 | 28.90 | 4.80 | 16 | -0.33 | ↔ |
| Qayumi 2004 | 1.T | K | MCQ | Y | Read | 34.66 | 11.97 | 24 | 46.40 | 14.61 | 25 | 0.88 | ↑ |
| Secomb 2012 | 1.T | M | Surv | Y | Man | 370.69 | 51.43 | 15 | 345.55 | 26.00 | 13 | -0.60 | ↔ |
| Sobocan 2017 | 1.T | K | MCQ | N | Group | 69.88 | 10.14 | 17 | 69.25 | 5.61 | 16 | -0.08 | ↔ |
| Subramanian 2012 | 1.T | K | MCQ | N | Lect | 0.62 | 0.02 | 15 | 0.87 | 0.02 | 15 | 12.50 | ↑ |
| Tao 2011 | 1.T | K | ? | N | Group | 82.88 | 10.88 | 46 | 83.88 | 10.21 | 46 | 0.09 | ↔ |
| Vash 2007 | 1.T | K | Txt | Y | Ward | 6.80 | 1.50 | 22 | 7.00 | 1.50 | 23 | 0.13 | ↔ |
| Wang 2017 | 1.T | K | MCQ | N | Man | 0.80 | - | 18 | 0.78 | - | 15 | - | ↔ |
| Williams 2001 | 1.T | K | MCQ | N | Lect | 34.40 | 10.95 | 85 | 33.10 | 10.95 | 78 | -0.12 | ↔ |
| Gu 2017 | 2.B | K | MCQ | N | Trad | 65.36 | 8.93 | 14 | 73.31 | 9.27 | 13 | 0.87 | ↑ |
| Kononowicz 2012 | 2.B | K | TF | Y | Trad | 45.81 | 3.76 | 45 | 47.37 | 3.43 | 51 | 0.43 | ↑ |
| Lehmann 2015 | 2.B | K | KF | Y | Trad | 68.80 | 16.30 | 30 | 92.20 | 4.70 | 27 | 1.91 | ↑ |
| Succar 2013 | 2.B | K | MCQ | N | Trad | 14.80 | 2.20 | 74 | 16.00 | 1.80 | 76 | 0.60 | ↑ |
| Wahlgren 2006 | 2.B | K | Txt | N | Trad | 0.88 | 0.08 | 81 | 0.89 | 0.07 | 28 | 0.17 | ↔ |
| Courteille 2018 | 3.C | K | MCQ | N | Video | - | - | 64 | - | - | 74 | - | ↔ |
| Trudeau 2017 | 3.C | K | MCQ | Y | Tutorial | 160.7 | 19.27 | 104 | 158.26 | 18.98 | 103 | -0.13 | ↔ |
| Berger 2017 | 4.V | K | ? | N | V9 | 6.81 | 1.10 | 41 | 7.07 | 1.50 | 30 | 0.20 | ↔ |
| Braun 2017 | 4.V | K | Mix | Y | V10 | 14.80 | 4.00 | 45 | 15.10 | 3.70 | 43 | 0.08 | ↔ |
| Davids 2014 | 4.V | K | Txt | N | V1 | 7.40 | 3.20 | 27 | 6.60 | 2.50 | 27 | -0.28 | ↔ |
| Friedman 1991 | 4.V | K | MCQ | N | V2 | 13.70 | 2.52 | 26 | 11.60 | 2.22 | 24 | -0.88 | ↑ |
| Harris 2013 | 4.V | K | Mix | Y | V3 | 211.10 | 28.14 | 32 | 202.10 | 26.70 | 38 | -0.33 | ↔ |
| Mahnken 2011 | 4.V | K | MCQ | N | V4 | 0.13 | 0.24 | 32 | 0.15 | 0.20 | 32 | 0.10 | ↔ |
| Maier 2013 | 4.V | K | Txt | N | V5 | 35.50 | 2.80 | 99 | 35.90 | 3.60 | 98 | 0.12 | ↔ |
| Tolsgaard 2016 | 4.V | K | MRQ | Y | V6 | 61.40 | 5.20 | 20 | 62.60 | 5.70 | 19 | 0.22 | ↔ |

**Cp** (Type of comparison): 1.T=Virtual patient vs traditional education; 2.B=Virtual patient blended learning vs traditional education; 3.C=Virtual patient vs other types of digital education; 4.V=Virtual patient design comparison.
**ST** (Subtype of competence): K=Core Knowledge; M=Meta-Knowledge.
**AM** (Assessment method): MCQ=Multiple Choice Questions; MRQ=Multiple Response Questions; TF=True/False; KF=Key Feature; Txt=Freetext answer; Surv=Survey; Mix=Mix of methods; ?=Unclear.
**V** (Was the assessment tool validated?): Y=Yes; N=No or unclear.
**Control** (Intervention in control group): Read=Reading assignment; Group=Group (collaborative) activity; Lect=Lecture; Man=Mannequin; Ward=Bedside teaching; Paper C.=Paper-based case; Trad=Traditional (when virtual patient is blended with traditional education); Mix=Mix of methods (e.g. lecture followed by small group exercise with mannequin); Video=Video-based module; Tutorial=Web-tutorial, e-module or on-line course; V1=Addition of usability enhancements; V2=Pedagogic (menus, guided) vs high-fidelity (free text, unguided) format; V3=Worked vs unworked cases; V4=Self-determined vs mandatory use; V5=Spaced vs non-spaced activation; V6=Solving vs constructing virtual patients; V9=Linear vs Branched; V10=Addition of scaffolding; A glossary of terms is available in Multimedia Appendix 1.
**M1,M2** (Arithmetic mean in control and intervention group); **SD1,SD2** (Standard deviation); **n1,n2** (Number of participants in study group); **SMD** (Standardised Mean Difference);
**Sum** (Summary of results): ↑=Intervention (second) group better; ↔=No significant difference; ↓=Control (first) group better.

**Table 3 Characteristics of studies assessing skills**

| **Study** | **Cp** | **ST** | **AM** | **V** | **Control** | **M1** | **SD1** | **n1** | **M2** | **SD2** | **n2** | **SMD** | **Sum** |
| --- | --- | --- | --- | --- | --- | --- | --- | --- | --- | --- | --- | --- | --- |
| Bonnetain 2010 | 1.T | Proc | Man | Y | Mix | 11.13 | 1.56 | 14 | 16.21 | 2.11 | 14 | 2.74 | ↑ |
| Botezatu 2010 | 1.T | CR | Mix | Y | Mix | 6.28 | 1.22 | 24 | 7.93 | 1.20 | 25 | 1.37 | ↑ |
| Botezatu 2010b | 1.T | CR | Mix | Y | Mix | 2.02 | 1.10 | 51 | 3.96 | 0.89 | 55 | 1.95 | ↑ |
| Fleetwood 2000 | 1.T | Com | SP | Y | Mix | 0.86 | 0.14 | 88 | 0.83 | 0.16 | 84 | -0.20 | ↔ |
| Li 2013 | 1.T | CR | Txt | N | Lect | 20.00 | 9.90 | 30 | 30.70 | 8.80 | 30 | 1.14 | ↑ |
| Liaw 2014 | 1.T | Proc | Man | Y | Man | 33.27 | 7.50 | 26 | 36.65 | 5.59 | 31 | 0.52 | ↔ |
| Maleck 2001 | 1.T | CR | Txt | N | Group | 0.32 | - | 42 | 0.35 | - | 47 | - | ↔ |
| Qayumi 2004 | 1.T | Proc | SP | N | Read | 6.98 | 2.93 | 24 | 8.74 | 2.60 | 25 | 0.64 | ↑ |
| Schwid 1999 | 1.T | Proc | Man | N | Read | 29.20 | 4.90 | 22 | 34.90 | 5.00 | 23 | 1.15 | ↑ |
| Schwid 2001 | 1.T | Proc | Man | N | Read | 43.40 | 5.90 | 15 | 52.60 | 9.90 | 16 | 1.12 | ↑ |
| Tao 2011 | 1.T | Int | Man | N | Group | 79.77 | 11.23 | 46 | 87.09 | 12.56 | 46 | 0.61 | ↑ |
| Triola 2006 | 1.T | CR | Txt | N | SP | - | - | 32 | - | - | 23 | - | ↔ |
| Vash 2007 | 1.T | CR | Txt | Y | Ward | 0.61 | 0.18 | 22 | 0.65 | 0.18 | 23 | 0.22 | ↔ |
| Williams 2001 | 1.T | CR | Txt | N | Lect | 11.00 | 3.22 | 85 | 12.50 | 3.22 | 78 | 0.47 | ↑ |
| Bryant 2015 | 2.B | Int | SP | N | Trad | 51.63 | 14.28 | 38 | 46.96 | 12.11 | 22 | -0.35 | ↔ |
| Gu 2017 | 2.B | Proc | Man | N | Trad | 87.54 | 4.60 | 14 | 90.85 | 4.60 | 13 | 0.72 | ↔ |
| Kaltman 2018 | 2.B | Com | SP | Y | Trad | - | - | 39 | - | - | 60 | - | mix |
| Kononowicz 2012 | 2.B | Proc | Man | Y | Trad | - | - | 75 | - | - | 84 | - | mix |
| Lehmann 2015 | 2.B | Proc | Man | Y | Trad | 54.70 | 20.57 | 30 | 76.40 | 15.64 | 27 | 1.18 | ↑ |
| Schittek 2004 | 2.B | Com | RP | N | Trad | 4.00 | - | 23 | 5.00 | - | 26 | - | ↑ |
| Weverling 1996 | 2.B | CR | Txt | Y | Trad | 6.20 | 1.50 | 51 | 7.50 | 1.40 | 52 | 0.90 | ↑ |
| Dankbaar 2016 | 3.C | Proc | Man | Y | Tutorial | 0.80 | 0.08 | 16 | 0.77 | 0.10 | 25 | -0.32 | ↔ |
| Foster 2015 | 3.C | Com | SP | Y | Video | 0.55 | 0.30 | 34 | 0.64 | 0.34 | 33 | 0.27 | ↔ |
| Kumta 2003 | 3.C | Int | Mix | Y | Mix | 52.89 | 5.60 | 44 | 58.72 | 6.80 | 52 | 0.93 | ↑ |
| Bearman 2001 | 4.V | Com | SP | Y | V8 | 38.80 | 4.80 | 41 | 35.70 | 5.30 | 38 | -0.61 | ↓ |
| Braun 2017 | 4.V | CR | VP | N | V10 | 0.09 | 0.06 | 45 | 0.12 | 0.07 | 43 | 0.46 | ↑ |
| Foster 2016 | 4.V | Com | SP | Y | V7 | 2.27 | 0.21 | 17 | 2.91 | 0.16 | 35 | 3.60 | ↑ |
| Tolsgaard 2016 | 4.V | Int | SP | Y | V6 | 59.10 | 12.80 | 20 | 60.80 | 11.50 | 19 | 0.14 | ↔ |

**Cp** (Type of comparison): 1.T=Virtual patient vs traditional education; 2.B=Virtual patient blended learning vs traditional education; 3.C=Virtual patient vs other types of digital education; 4.V=Virtual patient design comparison.
**ST** (Subtype of competence): CR=Clinical reasoning; Proc=Procedural skills; Com=Communication skills; Int=Integrated performance; Crit=Critical thinking.
**AM** (Assessment method): Man=Mannequin; SP=Standardised patient; VP=Virtual patient; RP=Real patient; Txt=Freetext answer; MCQ=Multiple choice questions; Mix=Mix of methods (e.g. lecture followed by small group exercise with mannequin).
**V** (Was the assessment tool validated?);Y=Yes; N=No or unclear.
**Control** (Intervention in control group): Read=Reading assignment; Group=Group (collaborative) activity; Lect=Lecture; Man=Mannequin; Ward=Bedside teaching; SP=standardised patient; Trad=Traditional (when virtual patient is supplement); Tutorial=Web-tutorial, e-module or on-line course; Video=Video-based module; Mix=Mix of methods (e.g. lecture followed by small group exercise with mannequin); V6=Solving vs constructing virtual patients; V7=No feedback vs emphatic feedback; V8=Narrative vs problem-solving approach. V10=Addition of scaffolding; A glossary of terms is available in Multimedia Appendix 1.
**M1,M2** (Arithmetic mean in control and intervention group); **SD1,SD2** (Standard deviation); **n1,n2** (Number of participants in study group); **SMD** (Standardised Mean Difference).
**Sum** (Summary of results): ↑=Intervention (second) group better; ↔=No significant difference; ↓=Control (first) group better; mix=Outcome compared item by item with mixed result - no aggregation of scores was possible.

**Table 4. Characteristics of studies asses**sing attitudes

| **SID** | **Cp** | **V** | **Control** | **M1** | **SD1** | **n1** | **M2** | **SD2** | **n2** | **SMD** | **Q-** | **Q=** | **Q+** | **Subject of attitude** |
| --- | --- | --- | --- | --- | --- | --- | --- | --- | --- | --- | --- | --- | --- | --- |
| Fleetwood 2000 | 1.T | N | Mix | - | - | 88 | - | - | 84 | - | 0 | 2 | 1 | Preparedness to deal with ethical, legal, and communication issues |
| Jeimy 2018 | 1.T | N | Read | - | - | 29 | - | - | 23 | - | 0 | 5 | 0 | Confidence in diagnostic and management abilities |
| Triola 2006 | 1.T | N | SP | - | - | 32 | - | - | 23 | - | 0 | 5 | 0 | Comfort, reluctance and preparedness in caring for distress disorders patients |
| Wang 2017 | 1.T | N | Man | # | - | 18 | # | - | 15 | - | - | - | - | Comfort with contrast reaction management and teamwork |
| Williams 2001 | 1.T | N | Lect. | - | - | 85 | - | - | 78 | - | 6 | 2 | 0 | Perceived ability to assess, diagnose and manage anxiety |
| Deladisma 2009 | 2.B | N | Trad | - | - | ? | - | - | ? | - | 0 | 3 | 1 | Confidence in history taking and clinical breast examination |
| Kaltman 2018 | 2.B | Y | Trad | 21.17 | 3.31 | 29 | 22.08 | 2.25 | 39 | 0.33 | - | 1* | - | Communication-Related Self-Efficacy |
| Lehmann 2015 | 2.B | Y | Trad | 59.60 | 15.80 | 30 | 72.30 | 11.70 | 27 | 0.91 | - | - | 1* | Self-assessment of procedural knowledge and skills |
| Smith 2011 | 2.B | Y | Trad | ^ | - | 102 | ^ | - | 97 | - | - | - | - | Clinical Cultural Competence |
| Trudeau 2017 | 3.C | Y | Tutorial | 4.17 | 0.62 | 106 | 4.06 | 0.60 | 99 | -0.18 | - | 1* | - | Attitude about opioid therapy |
| Berger 2017 | 4.V | N | V9 | # | - | 29 | # | - | 41 | - | - | - | - | Intention to perform pharmacy triage. Confidence in knowledge and skills to triage cough |

**Cp** (Type of comparison): 1.T=Virtual patient vs traditional education; 2.B=Virtual patient blended learning vs traditional education; 3.C=Virtual patient vs other types of digital education; 4.V=Virtual patient design comparison.
**V** (Was the questionnaire validated?): Y=Yes; N=No or unclear.
**Control** (Intervention in control group): Trad=Traditional (when virtual patient is supplement); Group=Group (collaborative) activity; Read=Reading assignment; Paper C.=Paper-based case; Lect=Lecture; Man=Mannequin; SP=Standardised patient; Mix=Mix of methods (e.g. lecture followed by small group exercise with mannequin); Tutorial=Web-tutorial, e-module or on-line course; V9=Linear vs Branched .
**M1,M2** (Arithmetic mean in control and intervention group); **SD1,SD2** (Standard deviation); **n1,n2** (Number of participants in study group); **SMD** (Standardised Mean Difference).
**Q-** (Number of items in questionnaire in favour of control (first) group); **Q=** (Number of items in questionnaire without significant difference); **Q+** (Number of items in questionnaire in favour of intervention (second) group)
*) One aggregated score; ^) Results presented separately for subgroups (bilingual and English speaking students) ?) Number of participants in subgroups unclear: #) Compared item-by-item and presented p-values of pre-/post-test comparisons only

Table 5. Characteristics of studies assessing satisfaction

| **SID** | **Cp** | **V** | **Control** | **M1** | **SD1** | **n1** | **M2** | **SD2** | **n2** | **SMD** | **Q-** | **Q=** | **Q+** |
| --- | --- | --- | --- | --- | --- | --- | --- | --- | --- | --- | --- | --- | --- |
| Al-Dahir 2014 | 1.T | Y | Group | - | - | 60 | - | - | 59 | - | 2 | 8 | 1 |
| Haerling 2018 | 1.T | Y | Man | 82.57 | 9.88 | 44 | 78.99 | 10.73 | 37 | -0.35 | - | 1* | - |
| Jeimy 2018 | 1.T | N | Read | - | - | 29 | - | - | 23 | - | 0 | 11 | 0 |
| Leong 2003 | 1.T | N | Read | - | - | 27 | - | - | 27 | - | 0 | 1 | 0 |
| Li 2013 | 1.T | Y | Lect | - | - | 30 | - | - | 30 | - | 0 | 4 | 6 |
| Maleck 2001 | 1.T | N | Paper C. | - | - | 42 | - | - | 47 | - | 1 | 3 | 0 |
| Miedzybrodzka 2001 | 1.T | N | Group | - | - | 32 | - | - | 16 | - | 0 | 4 | 0 |
| Qayumi 2004 | 1.T | N | Read | - | - | 24 | - | - | 25 | - | 0 | 2 | 6 |
| Triola 2006 | 1.T | N | SP | - | - | 32 | - | - | 23 | - | 0 | 1 | 0 |
| Wang 2017 | 1.T | N | Man | - | - | 18 | - | - | 15 | - | 5 | 1 | 0 |
| Courteille 2018 | 3.C | N | Video | ^ | - | 74 | ^ | - | 64 | - | - | - | - |
| Dankbaar 2016 | 3.C | Y | Tutorial | - | - | 16 | - | - | 25 | - | 0 | 0 | 2 |
| Foster 2015 | 3.C | Y | Video | - | - | 34 | - | - | 33 | - | 0 | 1 | 4 |
| Berger 2017 | 4.V | N | V9 | - | - | 41 | - | - | 29 | - | 0 | 12 | 2 |
| Davids 2014 | 4.V | Y | V1 | 76.60 | 18.20 | 27 | 81.50 | 12.90 | 27 | 0.31 | - | 1* | - |
| Friedman 1991 | 4.V | N | V2 | 30.52 | 5.94 | 26 | 37.42 | 6.22 | 24 | 1.14 | - | - | 1* |
| Tolsgaard 2016 | 4.V | N | V6 | 4.10 | 0.60 | 20 | 4.00 | 0.70 | 19 | -0.15 | - | 1* | - |

**Cp** (Type of comparison): 1.T=Virtual patient vs traditional education; 3.C=Virtual patient vs other types of digital education; 4.V= Virtual patient design comparison.
**V** (Was the assessment tool validated?) Y=Yes; N=No or unclear.
Control (Intervention in control group); Read=Reading assignment; Group=Group (collaborative) activity; Lect=Lecture; Man=Mannequin; Paper C.=Paper-based case; SP=Standardised patient; Tutorial=Web-tutorial, e-module or on-line course; Video=Video-based module; V1=Addition of usability enhancements; V2=Pedagogic (menus, guided) vs high-fidelity (free text, unguided) format; V6=Solving vs constructing virtual patients; V9=Linear vs Branched.
**M1,M2** (Arithmetic mean in control and intervention group); **SD1,SD2** (Standard deviation); **n1,n2** (Number of participants in study group); **SMD** (Standardised Mean Difference).
**Q-** (Number of items in questionnaire in favour of control (first) group); **Q=** (Number of items in questionnaire without significant difference); **Q+** (Number of items in questionnaire in favour of intervention (second) group)
*) one aggregated score; ^) Results presented graphically only, without measure of dispersion and separately for subgroups (students/residents)

**Table 6**. Technical characteristics of the included virtual patient interventions

| **Study** | **VP System** | **Logic** | **Control** | **F. delivery** | **F. timing** | **Video** |
| --- | --- | --- | --- | --- | --- | --- |
| Al-Dahir 2014 | DecisionSim/VP Sim | B | M | C | D | ? |
| Bearman 2001 | - | O | M | C | D | Y |
| Berger 2017 | ITyStudio (authoring tool) | B | M | C | D | N |
| Bonnetain 2010 | MicroSim | F, p | M | C | P | ? |
| Botezatu 2010 | Web-SP | F | M | C | P | ? |
| Botezatu 2010a | Web-SP | F | M | C | P | ? |
| Braun 2017 | CASUS | L | M | N | N | N |
| Bryant 2015 | Shadow Health Digital Clinical Experience (DCE) | F | K | C | D/P | N |
| Courteille 2018 | - | L | M | C | P | ? |
| Dankbaar 2016 | abcdeSIM | F, p | M | C | D/P | N |
| Davids 2014 | Electrolyte Workshop - HandsOn module | L, p | M | C | D/P | N |
| Deladisma 2009 | VIPS/DIANA (Digital Animated Avator) | F | R | ? | ? | N |
| Fleetwood 2000 | MedEthEx Online | F | O | C | P | Y |
| Foster 2015 | Virtual People Factory | F | K | C | D | N |
| Foster 2016 | Virtual People Factory | F | K | C/T | P | Y |
| Friedman 1991 | - | O | O | C | D/P | N |
| Gu 2017 | vSim | F | M | C | P | N |
| Haerling 2018 | vSim | F | M | C | P | N |
| Harris 2013 | - | ? | M | C | P | Y |
| Jeimy 2018 | - | ? | M | ? | ? | N |
| Kandasamy 2009 | - | L | M | C | D | N |
| Kaltman 2018 | Articulate Storyline (authoring tool) | B | M | C | D | Y |
| Kinney 1997 | Physical Therapy Patient Simulator | F | M | ? | ? | N |
| Kononowicz 2012 | CASUS | L | M | C | D | Y |
| Kumta 2003 | - | L | O | T | D/P | N |
| Lehmann 2015 | CAMPUS Card | L | M | ? | ? | Y |
| Leong 2003 | - | ? | M | C | D | Y |
| Li 2013 | - | ? | M | ? | ? | Y |
| Liaw 2014 | e-RAPIDS | F, p | M | C | D/P | N |
| Mahnken 2011 | CASUS | L | M | C | D | N |
| Maier 2013 | CASUS | L | M | C | D | Y |
| Maleck 2001 | CASUS | L | M | ? | ? | N |
| Miedzybrodzka 2001 | - | L | M | C | ? | Y |
| Qayumi 2004 | CyberActive Technology CyberPatient | F | M | C | ? | N |
| Schittek 2004 | - | F | K | T | P | ? |
| Schwid 1999 | ACLS Simulator 3.11 | F, p | M | C | D/P | N |
| Schwid 2001 | Anesthesia Simulator 3.0 | F, p | M | T | P | N |
| Secomb 2012 | MicroSim | F | M | C | P | ? |
| Smith 2011 | - | L | M | C | D | Y |
| Sobocan 2017 | CASUS | L | M | C/T | D/P | ? |
| Subramanian 2012 | StepStone Interactive Medical Software | L | M | C | D | N |
| Succar 2013 | Virtual Ophthalmology Clinic (VOC) | F | M | T | P | Y |
| Tao 2011 | 'Simulative Training System' | ? | M | C | P | Y |
| Tolsgaard 2016 | Web-SP | F | M | N | N | N |
| Triola 2006 | - | F | M | C | P | Y |
| Trudeau 2017 | - | ? | ? | ? | ? | Y |
| Vash 2007 | - | F | O | C | P | ? |
| Wahlgren 2006 | NUDOV | F | M | C | P | Y |
| Wang 2017 | - | ? | ? | ? | ? | Y |
| Weverling 1996 | CASES | F | M | C | P | N |
| Williams 2001 | - | F | M | C | D | Y |

**VP System** (Name of the virtual patient system). **Logic** (Navigation scheme through the virtual patient content; explained in Multimedia Appendix 1 1): F=Free access (problem-solving); L=Linear; B=Branching; p=includes a dynamic physiology simulation; O=Other; ?=Unclear.
**Control** (Control mechanism): M=Menu-based; K=Keyboard entry (natural language dialog); R=Oral (speech recognition); O=Other. **F. delivery** (Feedback delivery): C=Computer-provided; T=Tutor/facilitator; C/T=Combination; N=No feedback; ?=Unclear.
**F. timing** (Feedback timing): D=During activity; P=Post-activity; D/P=Combination; N=No feedback; ?=Unclear.
**Video** (Whether video clips where included in virtual patient cases): Y=Yes; N=No; ?=Unclear.

**Table 7**. Educational characteristics of the included virtual patient interventions

| **Study** | **Topic** | **Lang** | **L. Nat** | **Src.VP** | **Group** | **G.n** | **Int.** | **Access** | **VP.n** | **Av [w]** | **T.use [min]** |
| --- | --- | --- | --- | --- | --- | --- | --- | --- | --- | --- | --- |
| Al-Dahir 2014 | Cardiology | English | Y | I | I | - | C | C | 1 | - | 118 |
| Bearman 2001 | Psychiatry/Psychology | English | Y | I | I | - | C | C | 1 | ? | 60 |
| Berger 2017 | Pharmacy | French | Y | I | I | - | C | H | 1 | 2 | - |
| Bonnetain 2010 | Life support & critical care | Unclear | ? | E | I | - | C | C | 1 | - | - |
| Botezatu 2010 | Internal medicine | Spanish | Y | I | I | - | C | C | 6 | 3 | 360 |
| Botezatu 2010a | Internal medicine | Spanish | Y | I | I | - | C | C | 6 | 3 | 360 |
| Braun 2017 | Internal medicine | German | Y | I | I | - | L | C | 4 | - | 59 |
| Bryant 2015 | Nursing | English | Y | E | I | - | C | ? | 1 | 7 | - |
| Courteille 2018 | Orthopaedics | Unclear | ? | I | I | - | C | C | 1 | - | 45 |
| Dankbaar 2016 | Life support & critical care | Dutch | Y | I | I | - | L | H | 6 | 4 | 95 |
| Davids 2014 | Physiology | English | ? | I | I | - | L | ? | 1 | - | 18 |
| Deladisma 2009 | Surgery | English | Y | I | I | - | C | C | 1 | 1 | 10 |
| Fleetwood 2000 | Bioethics | English | Y | I | I | - | C | ? | 2 | - | - |
| Foster 2015 | Psychiatry/Psychology | English | Y | I | I | - | C | ? | 1 | - | - |
| Foster 2016 | Psychiatry/Psychology | English | Y | I | I | - | L | C | 1 | - | - |
| Friedman 1991 | Internal medicine | English | Y | I | I | - | C | C | 1 | - | 60 |
| Gu 2017 | Nursing | English | N | E | I | - | C | H | 10 | 10 | 287 |
| Haerling 2018 | Life support & critical care | English | Y | E | I | - | L | C | 1 | - | 30 |
| Harris 2013 | Internal medicine | English | Y | I | I | - | L | H | 2 | 24 | 125/136 |
| Jeimy 2018 | Internal medicine | English | Y | I | I | - | L | ? | 1 | - | - |
| Kaltman 2018 | Communication skills | English | Y | I | I | - | C | H | 3 | 3 | - |
| Kandasamy 2009 | Internal medicine | English | Y | I | I | - | C | C | 1 | - | 9 |
| Kinney 1997 | Physiotherapy/Occupational therapy | English | Y | I | ? | - | L | C | 1 | - | 75 |
| Kononowicz 2012 | Life support & critical care | Polish | Y | I | I | - | C | H | 6 | 6 | 90 |
| Kumta 2003 | Surgery | Unclear | ? | I | G/I | mix | C | H | 8 | 3 | 306 |
| Lehmann 2015 | Life support & critical care | German | Y | I | I | - | L | H | 2 | 2 | 45 |
| Leong 2003 | Internal medicine | English | Y | I | I | - | C | ? | 1 | - | 77 |
| Li 2013 | Dermatology | Unclear | ? | ? | G | 10 | C | C | 5 | 7 | - |
| Liaw 2014 | Life support & critical care | English | ? | I | I | - | C | C | 5 | - | 120 |
| Mahnken 2011 | Radiology | German | Y | I | I | - | C | H | 10 | 1 | 101/114 |
| Maier 2013 | Pediatrics | German | Y | I | I | - | C | H | 16 | 3 | 207/222 |
| Maleck 2001 | Radiology | German | Y | I | G | 2-3 | C | C | 10 | 1 | 240 |
| Miedzybrodzka 2001 | Genetics | English | Y | I | ? | - | ? | ? | 1 | - | 16 |
| Qayumi 2004 | Internal medicine | Japanese | Y | E | ? | - | L | C | 1 | - | 240 |
| Schittek 2004 | Dentistry | Unclear | ? | I | I | - | C | ? | 1 | 1 | 20 |
| Schwid 1999 | Anesthesia | English | Y | E | I | - | C | ? | ? | 4 | 120 |
| Schwid 2001 | Anesthesia | English | Y | E | I | - | C | ? | 10 | 12 | - |
| Secomb 2012 | Cardiology | English | Y/N | E | I | - | L | ? | 2 | 1 | - |
| Smith 2011 | Intercultural communication | English | Y/N | I | I | - | C | ? | 1 | 1 | 45 |
| Sobocan 2017 | Internal medicine | English | N | E | G | 8-10 | C | C | 4 | 4 | 720 |
| Subramanian 2012 | Life support & critical care | English | Y | ? | I | - | ? | C | 2 | - | 60 |
| Succar 2013 | Ophthalmology | English | Y | I | I | - | C | ? | 10 | - | - |
| Tao 2011 | Life support & critical care | Unclear | ? | ? | I | - | C | C | 36 | 1 | 360 |
| Tolsgaard 2016 | Internal medicine | Danish | Y | I | ? | - | L | H | 4 | 1 | 360/540 |
| Triola 2006 | Psychiatry/Psychology | English | Y | I | I | - | C | C | 2 | 1 | 60 |
| Trudeau 2017 | Internal medicine | English | Y | I | I | - | L | H | 4 | 4 | 120/240 |
| Vash 2007 | Internal medicine | English | N | I | G | 2 | C | C | 14 | 7 | 840 |
| Wahlgren 2006 | Dermatology | Swedish | Y | I | ? | - | C | C | 5 | - | 480 |
| Wang 2017 | Radiology | English | Y | I | I | - | L | C | 5 | ? | 39 |
| Weverling 1996 | Neurology | Dutch | Y | ? | I | - | C | C | 20 | 5 | 540 |
| Williams 2001 | Psychiatry/Psychology | English | Y | I | G | 3-4 | C | C | 1 | - | 55 |

**Topic** (Topic of presented cases).
**Lang** (Language of presented cases).
**L. Nat** (Information whether the language of virtual patient was native to the majority of participants): Y=Yes; N=No; ?=Unclear; Y/N=Mix (As a factor in the study).
**Src.VP** (Was the content of virtual patient cases developed at the local institution?): I=Internal (virtual patient content developed at the institution); E=External (virtual patient content purchased as a package); ?=Unclear; G/I=Mix.
**Group** (Was the study an individual or group assignment?): G=Group; I=Individual; ?=Unclear.
**G.n** (Number of learners in collaborative learning group): -=not applies.
**Int.** (Integration in curriculum); C=In curriculum; L=Laboratory; ?=Unclear.
**Access** (Type of access to virtual patient simulation): C=Campus-based computer laboratory; H=From Home; ?=Unclear.
**VP.n** (Number of virtual patient cases presented in intervention).
**Av [w]** (Time when the virtual patients were available) [in weeks].
**T.use [min]** (Average duration of use of virtual patients in intervention) [in minutes].

# References

1. Braun LT, Zottmann JM, Adolf C, Lottspeich C, Then C, Wirth S, et al. Representation scaffolds improve diagnostic efficiency in medical students. Med Educ 2017 Nov;51(11):1118–1126. PMID: 28585351

2. Kononowicz AA, Krawczyk P, Cebula G, Dembkowska M, Drab E, Frączek B, et al. Effects of introducing a voluntary virtual patient module to a basic life support with an automated external defibrillator course: a randomised trial. BMC Med Educ 2012 Jun 18;12(1):41. PMID: 22709278

3. Mahnken AH, Baumann M, Meister M, Schmitt V, Fischer MR. Blended learning in radiology: is self-determined learning really more effective? Eur J Radiol 2011 Jun;78(3):384–7. PMID: 21288674

4. Maier EM, Hege I, Muntau AC, Huber J, Fischer MR. What are effects of a spaced activation of virtual patients in a pediatric course? BMC Med Educ 2013 Mar 28;13(1):45. PMID: 23537162

5. Maleck M, Fischer MR, Kammer B, Zeiler C, Mangel E, Schenk F, et al. Do computers teach better? A media comparison study for case-based teaching in radiology. Radiographics 2001;21(4):1025–32. PMID: 11452078

6. Sobocan M, Turk N, Dinevski D, Hojs R, Pecovnik Balon B. Problem-based learning in internal medicine: virtual patients or paper-based problems? Intern Med J 2017 Jan;47(1):99–103. PMID: 27800653

7. Botezatu M, Hult H, Tessma MK, Fors U. Virtual patient simulation: knowledge gain or knowledge loss? Med Teach 2010;32(7):562–8. PMID: 20653378

8. Botezatu M, Hult H, Tessma MK, Fors UGH. Virtual patient simulation for learning and assessment: Superior results in comparison with regular course exams. Med Teach 2010 Apr;32(10):845–50. PMID: 20854161

9. Tolsgaard MG, Jepsen RMHG, Rasmussen MB, Kayser L, Fors U, Laursen LC, et al. The effect of constructing versus solving virtual patient cases on transfer of learning: a randomized trial. Perspect Med Educ 2016 Feb;5(1):33–8. PMID: 26754313

10. Foster A, Chaudhary N, Murphy J, Lok B, Waller J, Buckley PF. The Use of Simulation to Teach Suicide Risk Assessment to Health Profession Trainees-Rationale, Methodology, and a Proof of Concept Demonstration with a Virtual Patient. Acad Psychiatry 2015 Dec;39(6):620–9. PMID: 25026950

11. Foster A, Chaudhary N, Kim T, Waller JL, Wong J, Borish M, et al. Using Virtual Patients to Teach Empathy: A Randomized Controlled Study to Enhance Medical Students’ Empathic Communication. Simul Healthc 2016 Jun;11(3):181–9. PMID: 26841278

12. Gu Y, Zou Z, Chen X. The Effects of vSIM for Nursing^TM^ as a Teaching Strategy on Fundamentals of Nursing Education in Undergraduates. Clin Simul Nurs 2017 Apr;13(4):194–197.

13. Haerling KA. Cost-Utility Analysis of Virtual and Mannequin-Based Simulation. Simul Healthc 2018 Feb;13(1):33–40. PMID: 29373382

14. Bonnetain E, Boucheix J-M, Hamet M, Freysz M. Benefits of computer screen-based simulation in learning cardiac arrest procedures. Med Educ 2010 Jul;44(7):716–22. PMID: 20636591

15. Secomb J, McKenna L, Smith C. The effectiveness of simulation activities on the cognitive abilities of undergraduate third-year nursing students: a randomised control trial. J Clin Nurs 2012 Dec;21(23–24):3475–84. PMID: 23145517

16. Berger J, Bawab N, De Mooij J, Sutter Widmer D, Szilas N, De Vriese C, et al. An open randomized controlled study comparing an online text-based scenario and a serious game by Belgian and Swiss pharmacy students. Curr Pharm Teach Learn 2018;10(3):267–276. PMID: 29764629

17. Kaltman S, Talisman N, Pennestri S, Syverson E, Arthur P, Vovides Y. Using Technology to Enhance Teaching of Patient-Centered Interviewing for Early Medical Students. Simul Healthc 2018 Jun;13(3):188–194. PMID: 29771814

18. Bearman M, Cesnik B, Liddell M. Random comparison of “virtual patient” models in the context of teaching clinical communication skills. Med Educ 2001 Sep;35(9):824–32. PMID: 11555219

19. Dankbaar MEW, Alsma J, Jansen EEH, van Merrienboer JJG, van Saase JLCM, Schuit SCE. An experimental study on the effects of a simulation game on students’ clinical cognitive skills and motivation. Adv Health Sci Educ Theory Pract 2016 Aug;21(3):505–21. PMID: 26433730

20. Liaw SY, Chan SW-C, Chen F-G, Hooi SC, Siau C. Comparison of virtual patient simulation with mannequin-based simulation for improving clinical performances in assessing and managing clinical deterioration: randomized controlled trial. J Med Internet Res 2014 Sep 17;16(9):e214. PMID: 25230684

21. Schwid HA, Rooke GA, Ross BK, Sivarajan M. Use of a computerized advanced cardiac life support simulator improves retention of advanced cardiac life support guidelines better than a textbook review. Crit Care Med 1999 Apr;27(4):821–4. PMID: 10321676

22. Schwid HA, Rooke GA, Michalowski P, Ross BK. Screen-based anesthesia simulation with debriefing improves performance in a mannequin-based anesthesia simulator. Teach Learn Med 2001;13(2):92–6. PMID: 11302037

23. Al-Dahir S, Bryant K, Kennedy KB, Robinson DS. Online virtual-patient cases versus traditional problem-based learning in advanced pharmacy practice experiences. Am J Pharm Educ 2014 May 15;78(4):76. PMID: 24850938

24. Davids MR, Chikte UME, Halperin ML. Effect of improving the usability of an e-learning resource: a randomized trial. Adv Physiol Educ 2014 Jun;38(2):155–60. PMID: 24913451

25. Friedman CP, France CL, Drossman DD. A randomized comparison of alternative formats for clinical simulations. Med Decis Making 1991;11(4):265–72. PMID: 1766329

26. Jeimy S, Wang JY, Richardson L. Evaluation of virtual patient cases for teaching diagnostic and management skills in internal medicine: A mixed methods study. BMC Res Notes 2018;11(1):1–7. PMID: 29871699

27. Harris JM, Sun H. A randomized trial of two e-learning strategies for teaching substance abuse management skills to physicians. Acad Med 2013 Sep;88(9):1357–62. PMID: 23887001

28. Leong SL, Baldwin CD, Adelman AM. Integrating Web-based computer cases into a required clerkship: development and evaluation. Acad Med 2003 Mar;78(3):295–301. PMID: 12634211

29. Li J, Li QL, Li J, Chen ML, Xie HF, Li YP, et al. Comparison of three problem-based learning conditions (real patients, digital and paper) with lecture-based learning in a dermatology course: a prospective randomized study from China. Med Teach 2013;35(2):e963-70. PMID: 23009254

30. Tao H. Computer-based simulative training system—a new approach to teaching pre-hospital trauma care. J Med Coll PLA 2011 Dec;26(6):335–344.

31. Trudeau KJ, Hildebrand C, Garg P, Chiauzzi E, Zacharoff KL. A Randomized Controlled Trial of the Effects of Online Pain Management Education on Primary Care Providers. Pain Med 2017 Jun 1;18(4):680–692. PMID: 28034967

32. Wang CL, Chinnugounder S, Hippe DS, Zaidi S, O’Malley RB, Bhargava P, et al. Comparative Effectiveness of Hands-on Versus Computer Simulation-Based Training for Contrast Media Reactions and Teamwork Skills. J Am Coll Radiol 2017 Jan;14(1):103-110.e3. PMID: 27815053

33. Bryant R, Miller CL, Henderson D. Virtual Clinical Simulations in an Online Advanced Health Appraisal Course. Clin Simul Nurs 2015 Oct;11(10):437–444.

34. Schittek Janda M, Mattheos N, Nattestad A, Wagner A, Nebel D, Färbom C, et al. Simulation of patient encounters using a virtual patient in periodontology instruction of dental students: design, usability, and learning effect in history-taking skills. Eur J Dent Educ 2004 Aug;8(3):111–9. PMID: 15233775

35. Deladisma AM, Gupta M, Kotranza A, Bittner JG, Imam T, Swinson D, et al. A pilot study to integrate an immersive virtual patient with a breast complaint and breast examination simulator into a surgery clerkship. Am J Surg 2009 Jan;197(1):102–6. PMID: 19101251

36. Fleetwood J, Vaught W, Feldman D, Gracely E, Kassutto Z, Novack D. MedEthEx Online: a computer-based learning program in medical ethics and communication skills. Teach Learn Med 2000 Apr;12(2):96–104. PMID: 11228685

37. Kumta SM, Tsang PL, Hung LK, Cheng JCY. Fostering critical thinking skills through a web-based tutorial programme for final year medical students--A randomized controlled study. J Educ Multimed Hypermedia 2003;12(3):267–273.

38. Vash JH, Yunesian M, Shariati M, Keshvari A, Harirchi I. Virtual patients in undergraduate surgery education: a randomized controlled study. ANZ J Surg 2007;77(1–2):54–9. PMID: 17295822

39. Succar T, Zebington G, Billson F, Byth K, Barrie S, McCluskey P, et al. The impact of the Virtual Ophthalmology Clinic on medical students’ learning: a randomised controlled trial. Eye (Lond) 2013 Oct;27(10):1151–7. PMID: 23867718

40. Courteille O, Fahlstedt M, Ho J, Hedman L, Fors U, von Holst H, et al. Learning through a virtual patient vs. recorded lecture: a comparison of knowledge retention in a trauma case. Int J Med Educ 2018 Mar 28;9:86–92. PMID: 29599421

41. Smith BD, Silk K. Cultural competence clinic: an online, interactive, simulation for working effectively with Arab American Muslim patients. Acad Psychiatry 2011;35(5):312–6. PMID: 22007089

42. Subramanian A, Timberlake M, Mittakanti H, Lara M, Brandt ML. Novel educational approach for medical students: improved retention rates using interactive medical software compared with traditional lecture-based format. J Surg Educ 2012;69(2):253–6. PMID: 22365876

43. Triola M, Feldman H, Kalet AL, Zabar S, Kachur EK, Gillespie C, et al. A randomized trial of teaching clinical skills using virtual and live standardized patients. J Gen Intern Med 2006 May;21(5):424–9. PMID: 16704382

44. Weverling GJ, Stam J, ten Cate TJ, van Crevel H. [Computer-assisted education in problem-solving in neurology; a randomized educational study]. Ned Tijdschr Geneeskd 1996 Feb 24;140(8):440–3. PMID: 8720819

45. Kandasamy T, Fung K. Interactive Internet-based cases for undergraduate otolaryngology education. Otolaryngol Head Neck Surg 2009 Mar;140(3):398–402. PMID: 19248951

46. Wahlgren C-F, Edelbring S, Fors U, Hindbeck H, Ståhle M. Evaluation of an interactive case simulation system in dermatology and venereology for medical students. BMC Med Educ 2006 Aug 14;6:40. PMID: 16907972

47. Clark RC. Scenario-based e-learning: Evidence-based guidelines for online workforce learning. San Francisco: John Wiley & Sons.; 2013.

48. Qayumi AK, Kurihara Y, Imai M, Pachev G, Seo H, Hoshino Y, et al. Comparison of computer-assisted instruction (CAI) versus traditional textbook methods for training in abdominal examination (Japanese experience). Med Educ 2004 Oct;38(10):1080–8. PMID: 15461653

49. Lehmann R, Thiessen C, Frick B, Bosse HM, Nikendei C, Hoffmann GF, et al. Improving Pediatric Basic Life Support Performance Through Blended Learning With Web-Based Virtual Patients: Randomized Controlled Trial. J Med Internet Res 2015 Jul 2;17(7):e162. PMID: 26139388

50. Williams C, Aubin S, Harkin P, Cottrell D. A randomized, controlled, single-blind trial of teaching provided by a computer-based multimedia package versus lecture. Med Educ 2001 Sep;35(9):847–54. PMID: 11555222

51. Miedzybrodzka Z, Hamilton NM, Gregory H, Milner B, Frade I, Sinclair T, et al. Teaching undergraduates about familial breast cancer: comparison of a computer assisted learning (CAL) package with a traditional tutorial approach. Eur J Hum Genet 2001 Dec;9(12):953–6. PMID: 11840198

52. Kinney P, Keskula DR, Perry JF. The effect of a computer assisted instructional program on physical therapy students. J Allied Health 1997;26(2):57–61. PMID: 9268782

53. Cook DA, Zendejas B, Hamstra SJ, Hatala R, Brydges R. What counts as validity evidence? Examples and prevalence in a systematic review of simulation-based assessment. Adv Health Sci Educ Theory Pract 2014 May;19(2):233–50. PMID: 23636643

54. Harris JM, Sun H. The Physicians’ Competence in Substance Abuse Test (P-CSAT): a multidimensional educational measurement tool for substance abuse training programs. Drug Alcohol Depend 2012 May 1;122(3):236–40. PMID: 22055011

55. Harris JM, Fulginiti J V, Gordon PR, Elliott TE, Davis BE, Chabal C, et al. KnowPain-50: a tool for assessing physician pain management education. Pain Med 2008;9(5):542–54. PMID: 18266812

56. Bordage G, Grant J, Marsden P. Quantitative assessment of diagnostic ability. Med Educ 1990 Sep;24(5):413–25. PMID: 2215294

57. Black AE, Church M. Assessing medical student effectiveness from the psychiatric patient’s perspective: the Medical Student Interviewing Performance Questionnaire. Med Educ 1998 Sep;32(5):472–8. PMID: 10211287

58. Bylund CL, Makoul G. Empathic communication and gender in the physician-patient encounter. Patient Educ Couns 2002 Dec;48(3):207–16. PMID: 12477605

59. Liaw SY, Rethans J-J, Scherpbier A, Piyanee K-Y. Rescuing A Patient In Deteriorating Situations (RAPIDS): A simulation-based educational program on recognizing, responding and reporting of physiological signs of deterioration. Resuscitation 2011 Sep;82(9):1224–30. PMID: 21664026

60. Pangaro LN. A shared professional framework for anatomy and clinical clerkships. Clin Anat 2006 Jul;19(5):419–28. PMID: 16317746

61. Whitfield RH, Newcombe RG, Woollard M. Reliability of the Cardiff Test of basic life support and automated external defibrillation version 3.1. Resuscitation 2003 Dec;59(3):291–314. PMID: 14659599

62. Adamson KA, Gubrud P, Sideras S, Lasater K. Assessing the reliability, validity, and use of the Lasater Clinical Judgment Rubric: three approaches. J Nurs Educ 2012 Feb;51(2):66–73. PMID: 22132718

63. Todd M, Manz JA, Hawkins KS, Parsons ME, Hercinger M. The development of a quantitative evaluation tool for simulations in nursing education. Int J Nurs Educ Scholarsh 2008;5(1):Article 41. PMID: 19049492

64. Brooke J. SUS-A quick and dirty usability scale. Usability Eval Ind 1996;189(194):4–7.

65. Higgins JPT, Green S. Cochrane handbook for systematic reviews of interventions. Chichester: John Wiley & Sons.; 2008.
