# Supplementary material for: Virtual Patient Simulations in Health Professions Education: Systematic Review and Meta-Analysis by the Digital Health Education Collaboration
Source: J Med Internet Res. 2019 Jul 2;21(7):e14676. doi: 10.2196/14676 (PMC6632099; doi:10.2196/14676)
Supplement: Multimedia Appendix 5 [file jmir_v21i7e14676_app5.doc]

# Multimedia Appendix 5: Summary of excluded studies

On a closer examination of the acquired full-text versions of screened abstracts, we excluded 67 studies due to three major reasons: mismatched type of study design, intervention and comparison. Frequently multiple exclusion criteria applied.

Following the protocol, we excluded the following ineligible study designs: cross-over studies [1–7], studies without randomisation [8,9], no head-to-head comparison conducted across consecutive years [10–14], time series studies [15,16], qualitative method analyses as focus group studies [17] and case studies without comparison group [18,19].

Several interventions initially regarded as potential virtual patient simulations did not meet the inclusion criteria of this review. These were 3D immersive virtual reality environments in which the learner explored the virtual world by navigating a digital character [20–24], interventions involving mechanical mannequins [25–28], interventions with standardized patients - i.e. human actors role-playing patients [29–32], interventions where the responses of a virtual patient were human-controlled [33–37], studies where the virtual patient simulation required additional equipment as external probes or head mounted displays [38–40], those focused on simulation of a single organ or body part [41,42], low interactivity tutorials and short case vignettes including PowerPoint presentations of cases and e-Learning web sites where the simulation was not a gradually unfolding, interactive patient scenario [43–47], studies in which virtual patient simulation was intangibly mingled with other types of electronic content as instructional e-modules and videos, tutorials, flowcharts, diagnostic image collections [48–50], interventions in which virtual patient simulation were directly applied for knowledge, skills or attitudes assessment without a virtual patient-based learning phase to which the differences in performance could be attributed [51–55]. This category also included other types of interventions which could not be classified into the previously listed groups - as clinical decision support systems [56,57], emergency department simulations of patient flows comprised of short case vignettes [58], virtual human but not patients (e.g. interprofessional communication with staff members [59]) and simulation of procedures as cement handling in dentistry [60].

Finally, we excluded studies based on the type of comparison excluding comparisons to no intervention where the learners had not been given an alternative method to acquire the assessed competence within the study timeframe [61–69], and those comparison in which the interventions differed by a non-technology related parameter - e.g. type of applied paper-based virtual patient advance organizer [70], number of cases presented [71], presence of a human expert in the debriefing [72], order of sequencing lectures and virtual patients [73], collaboration setting: individual or small group learning [74]. We did not consider studies reporting types of outcomes not included in the protocol of the review – e.g. virtual patients usage patterns [75].

We did not consider in this review doctoral theses unless the results were subsequently published in a scientific journal. The rationale for that is that doctoral theses are not peer-reviewed. This excluded the following three studies: [76–78].

# References

1. Bateman J. Virtual patient design in undergraduate education. PhD [dissertation]. University of Warwick; 2013.

2. Bediang G, Franck C, Raetzo M-A, Doell J, Ba M, Kamga Y, et al. Developing clinical skills using a virtual patient simulator in a resource-limited setting. Stud Health Technol Inform 2013;192(1–2):102–6. PMID: 23920524

3. Cobbett S, Snelgrove-Clarke E. Virtual versus face-to-face clinical simulation in relation to student knowledge, anxiety, and self-confidence in maternal-newborn nursing: A randomized controlled trial. Nurse Educ Today 2016 Oct;45:179–84. PMID: 27537670

4. Lyon HC, Healy JC, Bell JR, O’Donnell JF, Shultz EK, Wigton RS, et al. Findings from an evaluation of PlanAlyzer’s double cross-over trials of computer-based, self-paced, case-based programs in anemia and chest pain diagnosis. Proceedings Symp Comput Appl Med Care 1991;88–93. PMID: 1807738

5. McCoy L. Virtual Patient Simulations for Medical Education: Increasing Clinical Reasoning Skills through Deliberate Practice. PhD [dissertation]. Arizona State University; 2014.

6. Tait L, Lee K, Rasiah R, Cooper JM, Ling T, Geelan B, et al. Simulation and Feedback in Health Education: A Mixed Methods Study Comparing Three Simulation Modalities. Pharm (Basel, Switzerland) 2018 May 3;6(2):41. PMID: 29751528

7. Thompson GA, Morrison RG, Holyoak KJ, Clark TK. Evaluation of an Online Analogical Patient Simulation Program. 19th IEEE Symp Comput Med Syst 2006. p. 623–628.

8. Barnett SG, Gallimore CE, Pitterle M, Morrill J. Impact of a Paper vs Virtual Simulated Patient Case on Student-Perceived Confidence and Engagement. Am J Pharm Educ 2016 Feb 25;80(1):16. PMID: 26941442

9. Heist BS, Kishida N, Deshpande G, Hamaguchi S, Kobayashi H. Virtual patients to explore and develop clinical case summary statement skills amongst Japanese resident physicians: a mixed methods study. BMC Med Educ 2016 Feb 1;16(1):39. PMID: 26830910

10. Gupta A, Singh S, Khaliq F, Dhaliwal U, Madhu S V. Development and validation of simulated virtual patients to impart early clinical exposure in endocrine physiology. Adv Physiol Educ 2018 Mar 1;42(1):15–20. PMID: 29341815

11. Kim S, Willett LR, Pan WJ, Afran J, Walker JA, Shea JA. Impact of Required Versus Self-Directed Use of Virtual Patient Cases on Clerkship Performance: A Mixed-Methods Study. Acad Med 2018 May;93(5):742–749. PMID: 29045276

12. Matsumura Y, Shinno H, Mori T, Nakamura Y. Simulating Clinical Psychiatry for Medical Students: a Comprehensive Clinic Simulator with Virtual Patients and an Electronic Medical Record System. Acad Psychiatry Academic Psychiatry; 2018;42(5):613–621.

13. Muntean V, Calinici T, Tigan S, Fors UGH. Language, culture and international exchange of virtual patients. BMC Med Educ 2013 Feb 11;13(1):21. PMID: 23394453

14. Palmer E, Devitt P. The assessment of a structured online formative assessment program: a randomised controlled trial. BMC Med Educ 2014 Jan 9;14(1):8. PMID: 24400883

15. Schneider A-T, Albers P, Müller-Mattheis V. E-Learning in Urology: Implementation of the Learning and Teaching Platform CASUS® - Do Virtual Patients Lead to Improved Learning Outcomes? A Randomized Study among Students. Urol Int 2015 Feb 4;94(4):412–8. PMID: 25871786

16. Turner RE, Evers WD, Wood OB, Lehman JD, Peck LW. Computer-based simulations enhance clinical experience of dietetics interns. J Am Diet Assoc 2000 Feb;100(2):183–90. PMID: 10670390

17. Bridgemohan CF, Levy S, Veluz AK, Knight JR. Teaching paediatric residents about learning disorders: use of standardised case discussion versus multimedia computer tutorial. Med Educ 2005 Aug;39(8):797–806. PMID: 16048622

18. Lin C-W, Clinciu DL, Swartz MH, Wu C-C, Lien G-S, Chan C-Y, et al. An integrative OSCE methodology for enhancing the traditional OSCE program at Taipei Medical University Hospital--a feasibility study. BMC Med Educ 2013 Jul 26;13(1):102. PMID: 23885884

19. Wong TKS, Chung JWY. Diagnostic reasoning processes using patient simulation in different learning environments. J Clin Nurs 2002 Jan;11(1):65–72. PMID: 11845757

20. Bindoff I, Ling T, Bereznicki L, Westbury J, Chalmers L, Peterson G, et al. A Computer Simulation of Community Pharmacy Practice for Educational Use. Am J Pharm Educ 2014 Nov 15;78(9):168. PMID: 26056406

21. Ingrassia PL, Ragazzoni L, Carenzo L, Colombo D, Ripoll Gallardo A, Della Corte F. Virtual reality and live simulation: a comparison between two simulation tools for assessing mass casualty triage skills. Eur J Emerg Med 2015 Apr;22(2):121–7. PMID: 24841770

22. Jones VF, Rowland ML, Brueckner-Collins J, Mack A, Gault S, Richardson A, et al. Comparison of LGBT cultural sensitivity teaching methods using the virtual reality world of second life versus a traditional workshop format. J Investig Med 2014;62(1):533.

23. LeFlore JL, Anderson M, Zielke MA, Nelson KA, Thomas PE, Hardee G, et al. Can a virtual patient trainer teach student nurses how to save lives--teaching nursing students about pediatric respiratory diseases. Simul Healthc 2012 Feb;7(1):10–7. PMID: 22228285

24. Youngblood P, Harter PM, Srivastava S, Moffett S, Heinrichs WL, Dev P. Design, development, and evaluation of an online virtual emergency department for training trauma teams. Simul Healthc 2008;3(3):146–53. PMID: 19088658

25. Liaw SY, Wen TZ, Tang CL, Chiang S, Chan SWC. Enhancing safe care for a deteriorating patient: A simulation-based interprofessional learning in undergraduate medicine and nursing education. Ann Acad Med Singapore 2012;41(9 Suppl. 1):S22.

26. Robinson JD, Bray BS, Willson MN, Weeks DL. Using human patient simulation to prepare student pharmacists to manage medical emergencies in an ambulatory setting. Am J Pharm Educ 2011 Feb 10;75(1):3. PMID: 21451755

27. Stefaniak JE, Turkelson CL. Does the sequence of instruction matter during simulation? Simul Healthc 2014 Feb;9(1):15–20. PMID: 24096920

28. Wilkinson JS, Barake W, Smith C, Thakrar A, Johri AM. A Cautionary Tale: a Comparison of Condensed Teaching Strategies To Develop Hand-Held Cardiac Ultrasound Skills in Internal Medicine Residents. Can J Cardiol 2014;30(10 Suppl. 1):S313–S314.

29. Ali J, Adam RU, Sammy I, Ali E, Williams JI. The simulated Trauma Patient Teaching Module--does it improve student performance? J Trauma 2007 Jun;62(6):1416–20. PMID: 17563658

30. Ali J, Al Ahmadi K, Williams JI, Cherry RA. The standardized live patient and mechanical patient models--their roles in trauma teaching. J Trauma 2009 Jan;66(1):98–102. PMID: 19131811

31. Brown CM, Lloyd EC, Swearingen CJ, Boateng BA. Improving resident self-efficacy in pediatric palliative care through clinical simulation. J Pain Symptom Manage 2011;41(1):231–2.

32. Reis S, Sagi D, Eisenberg O, Kuchnir Y, Azuri J, Shalev V, et al. The impact of residents’ training in Electronic Medical Record (EMR) use on their competence: report of a pragmatic trial. Patient Educ Couns 2013 Dec;93(3):515–21. PMID: 24011647

33. Butow P, Cockburn J, Girgis A, Bowman D, Schofield P, D’Este C, et al. Increasing oncologists’ skills in eliciting and responding to emotional cues: evaluation of a communication skills training program. Psychooncology 2008 Mar;17(3):209–18. PMID: 17575560

34. McGillion M, Promislow S, Watt-Watson J, Hunter J, Stinson J, Campbell F, et al. The experience of learners in the University of Toronto interfaculty pain curriculum simulation trial. Pain Res Manag 2011;15(2):108.

35. McGillion M, Dubrowski A, Stremler R, Watt-Watson J, Campbell F, McCartney C, et al. The Postoperative Pain Assessment Skills pilot trial. Pain Res Manag 2011;16(6):433–9. PMID: 22184553

36. Quail M, Brundage SB, Spitalnick J, Allen PJ, Beilby J. Student self-reported communication skills, knowledge and confidence across standardised patient, virtual and traditional clinical learning environments. BMC Med Educ 2016 Feb 27;16(1):73. PMID: 26919838

37. Raupach T, Muenscher C, Anders S, Steinbach R, Pukrop T, Hege I, et al. Web-based collaborative training of clinical reasoning: a randomized trial. Med Teach 2009 Sep;31(9):e431-7. PMID: 19811180

38. Chung GKWK, Gyllenhammer RG, Baker EL, Savitsky E. Effects of simulation-based practice on focused assessment with sonography for trauma (FAST) window identification, acquisition, and diagnosis. Mil Med 2013 Oct;178(10 Suppl):87–97. PMID: 24084309

39. Persky S, Eccleston CP. Impact of genetic causal information on medical students’ clinical encounters with an obese virtual patient: health promotion and social stigma. Ann Behav Med 2011 Jun;41(3):363–72. PMID: 21136226

40. Persky S, Eccleston CP. Medical student bias and care recommendations for an obese versus non-obese virtual patient. Int J Obes (Lond) 2011 May;35(5):728–35. PMID: 20820169

41. Dlugaiczyk J, Thiemer M, Neubert C, Schorn BA, Schick B. The aVOR app increases medical students’ competence in treating Benign Paroxysmal Positional Vertigo (BPPV). Otol Neurotol 2018;39(5):e401–e406.

42. Hu A, Wilson T, Ladak H, Haase P, Doyle P, Fung K. Evaluation of a three-dimensional educational computer model of the larynx: voicing a new direction. J Otolaryngol Head Neck Surg 2010 Jun;39(3):315–22. PMID: 20470679

43. Carpenter KM, Cohn LG, Glynn LH, Stoner SA. Brief Interventions for Tobacco Users: Using the Internet to Train Healthcare Providers. Int Electron J Health Educ 2008 Feb 8;11:1–12. PMID: 22096413

44. McCrow J, Sullivan KA, Beattie ER. Delirium knowledge and recognition: a randomized controlled trial of a web-based educational intervention for acute care nurses. Nurse Educ Today 2014 Jun;34(6):912–7. PMID: 24393287

45. Moja L, Moschetti I, Cinquini M, Sala V, Compagnoni A, Duca P, et al. Clinical evidence continuous medical education: a randomised educational trial of an open access e-learning program for transferring evidence-based information - ICEKUBE (Italian Clinical Evidence Knowledge Utilization Behaviour Evaluation) - study protoc. Implement Sci 2008 Jul 17;3(1):37. PMID: 18637189

46. Mounsey A, Reid A. A randomized controlled trial of two different types of web-based instructional methods: one with case-based scenarios and one without. Med Teach 2012;34(9):e654-8. PMID: 22657939

47. Pusic M V., Pachev GS, MacDonald WA. Embedding medical student computer tutorials into a busy emergency department. Acad Emerg Med 2007 Feb;14(2):138–48. PMID: 17267530

48. Baumlin KM, Bessette MJ, Lewis C, Richardson LD. EMCyberSchool: an evaluation of computer-assisted instruction on the Internet. Acad Emerg Med 2000 Aug;7(8):959–62. PMID: 10958144

49. Durmaz A, Dicle A, Cakan E, Cakir Ş. Effect of screen-based computer simulation on knowledge and skill in nursing students’ learning of preoperative and postoperative care management: a randomized controlled study. Comput Inform Nurs 2012 Apr;30(4):196–203. PMID: 22228217

50. Taslibeyaz E, Dursun OB, Karaman S. Interactive video usage on autism spectrum disorder training in medical education. Interact Learn Environ Taylor & Francis; 2017;25(8):1025–1034.

51. Deladisma AM, Cohen M, Stevens A, Wagner P, Lok B, Bernard T, et al. Do medical students respond empathetically to a virtual patient? Am J Surg 2007 Jun;193(6):756–60. PMID: 17512291

52. Dickerson R, Johnsen K, Raij A, Lok B, Stevens A, Bernard T, et al. Virtual patients: assessment of synthesized versus recorded speech. Stud Health Technol Inform 2006;119:114–9. PMID: 16404028

53. Epstein JH, Levin M, Jowell MS. Agent based simulation for training and assessing students in the field of anesthesiology. Proc 26th IEEE Int Symp Comput Med Syst IEEE; 2013. p. 332–336.

54. Hege I, Dietl A, Kiesewetter J, Schelling J, Kiesewetter I. How to tell a patient’s story? Influence of the case narrative design on the clinical reasoning process in virtual patients. Med Teach 2018;40(7):736–742. PMID: 29490538

55. Raij AB, Johnsen K, Dickerson RF, Lok BC, Cohen MS, Duerson M, et al. Comparing interpersonal interactions with a virtual human to those with a real human. IEEE Trans Vis Comput Graph 2007;13(3):443–57. PMID: 17356212

56. Bacchus CM, Quinton C, O’Rourke K, Detsky AS. A randomized crossover trial of Quick Medical Reference (QMR) as a teaching tool for medical interns. J Gen Intern Med 1994 Nov;9(11):616–21. PMID: 7853070

57. Schwartz S, Griffin T. Comparing different types of performance feedback and computer-based instruction in teaching medical students how to diagnose acute abdominal pain. Acad Med. 1993. p. 862–4. PMID: 8216661

58. Franc-Law JM, Ingrassia PL, Ragazzoni L, Della Corte F. The effectiveness of training with an emergency department simulator on medical student performance in a simulated disaster. CJEM 2010 Jan;12(1):27–32. PMID: 20078915

59. Kron FW, Fetters MD, Scerbo MW, White CB, Lypson ML, Padilla MA, et al. Using a computer simulation for teaching communication skills: A blinded multisite mixed methods randomized controlled trial. Patient Educ Couns 2017 Apr;100(4):748–759. PMID: 27939846

60. Tubelo RA, Branco VLC, Dahmer A, Samuel SMW, Collares FM. The influence of a learning object with virtual simulation for dentistry: A randomized controlled trial. Int J Med Inform 2016 Jan;85(1):68–75. PMID: 26601728

61. Albright G, Bryan C, Adam C, McMillan J, Shockley K. Using Virtual Patient Simulations to Prepare Primary Health Care Professionals to Conduct Substance Use and Mental Health Screening and Brief Intervention. J Am Psychiatr Nurses Assoc 2017;24(3):247–259. PMID: 28754067

62. Fleming M, Olsen D, Stathes H, Boteler L, Grossberg P, Pfeifer J, et al. Virtual reality skills training for health care professionals in alcohol screening and brief intervention. J Am Board Fam Med 2009;22(4):387–98. PMID: 19587253

63. Lange LL, Haak SW, Lincoln MJ, Thompson CB, Turner CW, Weir C, et al. Use of Iliad to improve diagnostic performance of nurse practitioner students. J Nurs Educ 1997 Jan;36(1):36–45. PMID: 8986960

64. Legris M-È, Séguin NC, Desforges K, Sauvé P, Lord A, Bell R, et al. Pharmacist Web-based training program on medication use in chronic kidney disease patients: impact on knowledge, skills, and satisfaction. J Contin Educ Health Prof 2011;31(3):140–50. PMID: 21953653

65. Olson N, An-Grogan Y, Avula U, Mary M, Vozenilek JA, Salzman DH. Computerized decision simulation as an educational primer for high fidelity simulation. Acad Emerg Med 2012;19(Suppl. 1):S328.

66. Shoemaker MJ, de Voest M, Booth A, Meny L, Victor J. A virtual patient educational activity to improve interprofessional competencies: A randomized trial. J Interprof Care 2015 Nov;29(4):395–7. PMID: 25412759

67. Sperl-Hillen JM, O’Connor PJ, Rush WA, Johnson PE, Biltz G, Asche SA, et al. Personalized Physician Learning Intervention Improved Glucose Control in Adults With Diabetes. Diabetes 2009;58(Suppl. 1A):48.

68. Sperl-Hillen JA, O’Connor PJ, Ekstrom HL, Rush WA, Asche SE, Fernandes OD, et al. Educating resident physicians using virtual case-based simulation improves diabetes management: A randomized controlled trial. Acad Med 2014;89(12):1664–1673. PMID: 25006707

69. Taglieri CA, Crosby SJ, Zimmerman K, Schneider T, Patel DK. Evaluation of the Use of a Virtual Patient on Student Competence and Confidence in Performing Simulated Clinic Visits. Am J Pharm Educ 2017 Jun;81(5):87. PMID: 28720915

70. Kenny RF. The Generative Effects of Instructional Organizers with Computer-Based Interactive Video. J Educ Comput Res 1995;12(3):275–296.

71. Lowdermilk DL, Fishel AH. Computer simulations as a measure of nursing students’ decision-making skills. J Nurs Educ 1991 Jan;30(1):34–9. PMID: 1847408

72. O’Connor PJ, Sperl-Hillen JM, Johnson PE, Rush WA, Asche SE, Dutta P, et al. Simulated physician learning intervention to improve safety and quality of diabetes care: a randomized trial. Diabetes Care 2009 Apr;32(4):585–90. PMID: 19171723

73. Marei HF, Donkers J, Al-Eraky MM, van Merrienboer JJG. The effectiveness of sequencing virtual patients with lectures in a deductive or inductive learning approach. Med Teach 2017 Dec;39(12):1268–1274. PMID: 28936901

74. Marei HF, Donkers J, Van Merrienboer JJG. The effectiveness of integration of virtual patients in a collaborative learning activity. Med Teach 2018;40(sup1):S96–S103. PMID: 29730966

75. Stathakarou N, Scully M, Kononowicz A, Henningsohn L, Zary N, McGrath C. MOOC Learners’ Engagement with Two Variants of Virtual Patients: A Randomised Trial. Educ Sci 2018 Mar 29;8(2):44.

76. Bebeau D. Exploring the effectiveness of a virtual learning methodology in occupational therapy education. PhD [dissertation]. Edgewood College; 2015.

77. Huhn K. Virtual physical therapy clinician: Development, validation and testing. PhD [dissertation]. University of Medicine and Dentistry of New Jersey; 2011.

78. Ousley TL. The Development of Critical Thinking with Technology in Nursing Education. PhD [dissertation]. Northcentral University; 2012.
