# Supplementary material for: Virtual Patient Simulations in Health Professions Education: Systematic Review and Meta-Analysis by the Digital Health Education Collaboration
Source: J Med Internet Res. 2019 Jul 2;21(7):e14676. doi: 10.2196/14676 (PMC6632099; doi:10.2196/14676)
Supplement: Multimedia Appendix 6 [file jmir_v21i7e14676_app6.doc]

# Multimedia Appendix 6: Subgroup analysis

**Knowledge outcomes virtual patient vs traditional education**

We conducted a subgroup analysis of 11 variables (discipline, income category of country, integration into curriculum, collaborative learning, source of content, use of series of cases, case progression, communication method, timing of feedback, native language use and subtype of ability) and none of them explained the heterogeneity (I^2^<50% in both groups). Due to insufficient studies in respective categories we did not conduct a subgroup analysis for the following variables: registration stage and classroom-based learning.

**Skills outcomes virtual patient vs traditional education**

We conducted a subgroup analysis of 8 variables (discipline, income category of country, collaborative learning, source of content, use of series of cases, communication method, timing of feedback and subtype of ability) and none of them explained the heterogeneity in a sufficient manner (I^2^<50% in both groups). Due to insufficient studies in respective categories we did not conduct a subgroup analysis for the following variables: integration into curriculum, case progression, registration stage, classroom-based learning and native language use.

**Post-hoc analysis of passive vs active forms of learning**

We observed in the post-hoc analysis using albatross plots that comparisons of virtual patients to passive forms of learning (reading exercises, lectures) tended to display large positive effect sizes, whereas those comparing virtual patients to active learning (group discussion or mannequin-based learning) show small effects or even negative effects (left hand side in the figures). These findings inspired us to investigate the change in effect size depending on the passive/active form of traditional education using formal methods. We presented separately in the subgroup analysis studies in which the control group was a mix of active and passive traditional forms of education [1–5]. The subgroup analysis did not explain entirely the heterogeneity for knowledge and skills outcomes. Figure 1 shows the result of subgroup analysis for skills outcomes which gives some indications to confirm the hypothesis. The pooled effect on skills outcomes was large to moderate in comparison to passive traditional education interventions (SMD=0.82, 95% CI:[0.51,1.13], I^2^=40, n=348) and small to moderate in comparison to active traditional education (SMD=0.49, 95% CI:[0.20,0.77], I^2^=0, n=194) with no significant difference in the test for subgroups at p=0.17.

**
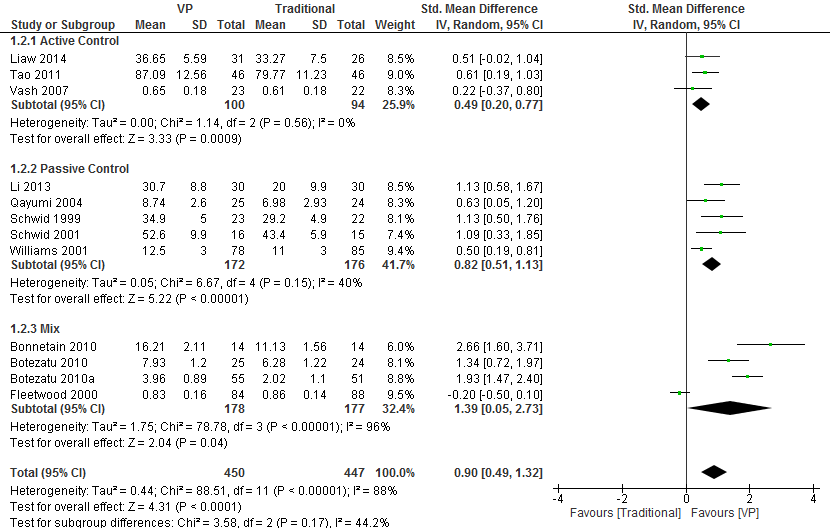
**

**Figure 1 Subgroup analysis comparing virtual patient to traditional education for skills outcome**

# References

1. Bonnetain E, Boucheix J-M, Hamet M, Freysz M. Benefits of computer screen-based simulation in learning cardiac arrest procedures. Med Educ 2010 Jul;44(7):716–22. PMID: 20636591

2. Botezatu M, Hult H, Tessma MK, Fors UGH. Virtual patient simulation for learning and assessment: Superior results in comparison with regular course exams. Med Teach 2010 Apr;32(10):845–50. PMID: 20854161

3. Botezatu M, Hult H, Tessma MK, Fors U. Virtual patient simulation: knowledge gain or knowledge loss? Med Teach 2010;32(7):562–8. PMID: 20653378

4. Fleetwood J, Vaught W, Feldman D, Gracely E, Kassutto Z, Novack D. MedEthEx Online: a computer-based learning program in medical ethics and communication skills. Teach Learn Med 2000 Apr;12(2):96–104. PMID: 11228685

5. Kinney P, Keskula DR, Perry JF. The effect of a computer assisted instructional program on physical therapy students. J Allied Health 1997;26(2):57–61. PMID: 9268782
