# Supplementary material for: Virtual Patient Simulations in Health Professions Education: Systematic Review and Meta-Analysis by the Digital Health Education Collaboration
Source: J Med Internet Res. 2019 Jul 2;21(7):e14676. doi: 10.2196/14676 (PMC6632099; doi:10.2196/14676)
Supplement: Multimedia Appendix 7 [file jmir_v21i7e14676_app7.doc]

# Multimedia Appendix 7: Quality of the evidence

## Risk of bias of included studies

### Allocation (selection bias)

In 17 out 51 studies the method for randomisation of participants into groups was adequate and described in sufficient detail. In most cases these were computer-generated lists [1–12] generated by a spreadsheet program (MS Excel), specialised statistical software programs, functionality of the learning management system (WebCT Vista), or the form of computer-generation was not specified. In three studies the randomisation was done by lottery [13–15], in two by random number tables [16,17]. In 31 studies the sequence generation process was not described in sufficient detail. We judged three studies as high risk in the domain of adequate sequence generation because of a low quality method of randomisation. In one study the randomisation was made by alternating from an alphabetised student roster [18]. In Mahnken et al [19] the details of sequence generation were not entirely clear but it was stated that allocation was consecutive. In Wang et al [20] participants were after randomisation swapped between groups because of unresolvable scheduling conflicts.

Only two studies explicitly described adequate allocation concealment. Davids et al [7] used sequentially numbered, opaque envelopes, whereas in Lehmann et al. [21] the students were "unaware of group allocation, which was randomly distributed by numbers". Two studies in which the sequence generation was consecutive were classified as high risk of bias of allocation concealment [18,19]. In the rest of the studies the allocation concealment was not explicitly described, hence we marked the risk as unclear, even though we doubt it had any influence on the study progress.

### Blinding (performance bias and detection bias)

Blinding of participants to group allocation was not possible in studies where virtual patients were compared with a traditional learning intervention. Our judgement is that this could introduce bias. The direction of the bias is difficult to predict as we are aware of the possibilities for a positive innovation bias towards technology-based intervention, as well as negative resistance to change caused for instance by the fear of diminished direct patient contact. This bias could be stronger in subjective outcomes (i.e. satisfaction surveys and attitude measurement) than objective outcomes (knowledge or skills measurements e.g. by MCQ or performance on standardised patients). Yet, we felt it would be unfair to explicitly mark those studies as high risk as the inability to blind study participants is a general methodological limitation in this field not characteristic to a particular study design.

In our opinion blinding of participants is possible when two new interventions are compared - e.g. different types of feedback in virtual patients. In such cases the participant might not be aware which part of the educational intervention is experimental which would lead to effective blinding. Nevertheless, the authors of only one study have recognised this possibility and declared explicitly: "The students themselves were not aware of the theoretical difference between the two types of tutorials or of the hypothesis under investigation." [22]. The other studies comparing virtual patient features did not comment this aspect and hence are to be marked as unclear on the risk of detection bias. Considering this together with the previously described inability to blind participants in comparison with traditional intervention we see little value in presenting performance bias in the risk of bias summary.

The assessment of outcome was in most cases judged as low risk. The outcomes were rated by MCQ tests which leave little space for subjective interpretation and often occurs automatically. In many papers the authors explicitly declared the assessor were not aware of the group allocation or used detailed checklist and worked in parallel to minimise bias. In the study by Liaw et al. [16] "to reduce bias, the participants were required to wear caps, gowns, and masks to blind their identities from the raters who may have known the participants’ training background". In the study by Kinney [23] the MCQ were graded by an Information Services Division Test Center. In seven studies it was unclear from the report whether the assessors were blinded [2,5,10,24–27]. The risk of bias due to lack of assessor bias was marked as high in three papers. In two studies the only outcome reported was a form of self-assessment [9,14], in one assessment of study groups were separated in time by one month [28]. We marked studies as low risk when they provided at least one outcome assessment method with low risk of bias.

### Incomplete outcome data (attrition bias)

Most of the studies reported limited and balanced attrition (36 out of 51). There were a few exceptions that raised our concern as high risk of bias. We noticed large differences in proportions of missing outcomes between intervention groups in three studies [1,20,29]. Five studies had a high proportion of missing outcomes [1,27,30–32]. In one study data were imputed which we classified as high risk of bias [18]. In seven studies we were unclear on the actual attrition or way of dealing with missing data [3,7,10,14,24,33,34].

### Selective reporting (reporting bias)

It is uncommon in medical education research to publish a protocol in advance of the actual study. Such protocols are often submitted to ethical review boards for approval but are not available for public access. Yet, we do not see much indication of reporting bias in the reviewed studies. We have concerns about missing or not detailed reported items in satisfaction questionnaires and checklist in [14,17,18,33,35,36]. In the study by Kumta et al. [37] the performance is measured using three methods but reported is just the aggregated score. Missing is partial score divided by different assessment modalities which could be an indication of bias. In the study by Bonnetain et al. [38] a pre-test was conducted, but the results are not presented in the paper, this is however unlikely to have significant impact on interpretation of data. Bearman et al. [22] reports for logistic reasons the results of only three out of six groups in the first week after the course which could, however, influence the results. In the studies [9,39] report satisfaction scores in a way that prevents us from calculating it separately for the intervention and control group. Berger et al [1] do not make a direct comparison between the intervention groups (just pre-/post analysis). Five studies [17,20,27,34,40] have missing measures of dispersion.

### Other potential sources of bias

Three studies had significant differences in baseline evaluation [19,20,41] which were marked as high risk of bias. To balance that it is important to say that in 16 out of 51 studies the study had no information or unclear information whether the groups were tested for baseline differences.

The calculated effect size in the study [42] was 12.5 which is unrealistically high. The most likely reason for that is error in the reported standard deviation. We attempted to contact the authors regarding this piece of information but without response. We have therefore marked the data as an outlier and excluded from calculations of pooled effect size.

The study by Bonnetain et al. [38] received a commercial grant for the research from the software developer. The authors declared it had no influence on the outcomes, but this cannot be entirely excluded. In the studies by Harris & Sun [29] and Trudeau et al. [3] a research grant was allocated to a for profit business employing the researchers. On this occasion, it is important to notice that in many studies in this review developers of the virtual patient software were among the authors or in acknowledgements of the study and it was not declared as a conflict of interest. Some of the products were distributed later or even at the time of study on commercial basis. The involvement of developers (both academic and commercial) could have a positive bias on the results yet this is a common practice in medical education informatics research and we do not evaluate it as a separate risk of bias.

In studies [3,11,13,29,39] the participants were financially compensated for the participation, in [6] the participants took part in a lottery as an incentive to participate in the study. We do not see this as source of bias.

In the study by Berger et al [1] participants were from two different countries with different curricula. This influenced the outcomes more than the tested intervention. Trudeau et al. [3] measured outcome in an uncontrolled environment (participants were tested at distance while working unsupervised at home). In two studies [16,20] participants were tested only on a modality which was the training method of just one of the study groups (unfamiliarity bias).

A potential issue could be volunteer bias. In ten studies [3,8,21,25,27,29,30,32,33,43] less than 50% of eligible participants in the cohort were willing or invited to participate in the study. We marked that as unclear risk of bias. In the study by Secomb et al. [32] only 5% of invited participants completed the study which we regard as high risk of bias. Unfortunately, the number of potential participants was available just in around half of the studies (26 of 51) and it is likely that this issue relates to more studies. For instance in [23] participated just 10 students from the junior year of physical therapy, but we do not know the total number of students in the year and the reasons why only ten of them completed the study.

### Risk of bias for cluster-randomised trials

In the three cluster-randomised trials included in the review [10,37,44] we rate the risk of recruitment bias as low because the clusters usually cover established groups of students in the programmes and it is therefore unlikely that the students were biased to be included in the trial after the clusters have been randomised. All studies with cluster RCT in the review had a baseline differences check in a pre-test and no significant differences between the study arms were found, which is rated by us as a low risk of baseline imbalance. There is no evidence of attrition of clusters in the trials. None of the three studies considered clusters in the statistical analysis and hence the risk of incorrect analysis is rated high. The likely reason why the authors of the studies preferred to randomise clusters instead of individuals is to decrease the risk of group contamination. At the same time, cluster randomisation is likely to increase the influence of confounding factors as teacher characteristics or internal group dynamics. The corrections to compensate inadequate statistical analysis did not change the outcome qualitatively which remained significant favouring virtual patient intervention in all three cases.

## Summary of quality of the evidence

Following the Cochrane methodology [45], we have evaluated the quality of evidence by judging the studies though an analysis of risk of bias. The prescribed risk of bias criteria were created for the purpose of reporting on clinical, not educational interventions. For that reason, they are not easy to apply in virtual patient educational research. Randomization and allocation concealment were often not described in sufficient details to make a judgment. There are also authors who question the relevance of those criteria pointing to results of meta-analyses both in the clinical and educational sectors which showed no difference in the effects of systematic bias across randomised and non-randomised studies [46].

Discussion amongst the review team caused us to question how to handle blinding of study participants. When a computer-based learning method is compared to an established non-computer based method, the blinding of students is inherently impossible. Consequently, a bias is possible, although it is debatable whether it will result in positive (novelty effect) or negative (resistance to change) effects. We have observed that the approach to this question varies among systematic review authors (e.g., [47] and [48]), some of those seeing it as a high or low risk of bias for the same studies. The point is, however, that a lack of student participant blinding is just the tip of an iceberg of problems which result from the comparison of computer and non-computer based forms of education, known in literature as media-comparative research [49,50]. Both Friedman and Cook argue that the limitations of this type of comparison boil down to the inability to produce an adequate control group since interventions are bound to be influenced by too many confounding factors to be generalisable. A simple remedy to that is to abandon this type of research as being impossible to implement rigorously and embark instead on comparisons on the same level of instructional design but not across media [50]. In this review this would limit us to the third and fourth type of comparison (virtual patient to e-Learning and comparison of virtual patients design variants) which is represented just by a handful of studies (n=5 for comparison of with e-Learning and, n=10 for virtual patient design comparisons). In those types of comparisons blinding is in fact possible, as a direct comparison of differences between two tested variations of a common resource e.g. the form of virtual patient guidance might be concealed from study participants [22]. Yet, since the expectation of curricular decision makers is still to see evidence showing that replacing a traditional method will not be detrimental to the students, there are still media-comparative studies published. We summarize them in this review which, unsurprisingly, leads to great heterogeneity and risk of bias. Yet, if we consider merely one possible confounding factor in many possible, as for instance the individual pedagogical quality of the lecturer in the control intervention, we see this may lead to significant differences across studies, very difficult to capture in the reports and summarise in reviews.

One more criterion to judge quality of evidence is the validity of instruments used in outcome measurements. A non-validated instrument is likely to delivery imprecise or even biased results. This was aptly summarised by Ruth Clark: "Tests that are not valid and not reliable are a waste of time at best, and misleading and/or illegal at worse" [51]. Yet, around 60% of knowledge, attitudes or satisfaction outcomes and 40% of skills outcomes lack information on validity of the instrument. In many studies validity referred to content (face) validity only and was judged by a local expert panel. Internal consistency (Cronbach Alpha) was reported in just 6 out of 33 knowledge tests. Established validated instruments were even less frequent: three of 33 for knowledge, six of 28 for skills, two of 11 for attitudes and one of 17 for satisfaction. It is difficult to judge how much it has in fact biased the results, but interestingly, out of those studies which used standardized validated tools only [39] (Empathic Communication Coding System) showed significant differences. It is easy to call to use more standardized and well validated instruments, but at the same time we should consider that for instance the number of standardized tests for knowledge will always be limited due to the breadth of topics covered. Standardization in clinical reasoning skills may lead to case-specific effects. The virtues of using existing validated and well-established tools may at the same time be compromised by the limited alignment of tasks involved in the intervention with the standardized tool. An example for that could be cognitive growth and meta-knowledge measuring tools like LEP (Learning Environment Preferences) in [32] or DTI (Diagnostic Thinking Inventory) in [43]. None of them showed any differences which might be due to a high level of measured knowledge abstraction within a short-term intervention, such that the period of intervention is too short to expect a change.

The attrition rate which involves those who drop out from the study after they were allocated to an intervention was in most studies (with a few exceptions) limited and well balanced, hence did not pose a risk. This, however, does not cover the volunteer bias which is frequently not discussed in the papers. Those students who are willing to participate in research are often high achievers [52]. Yet, those students are at the same time known to benefit less from variations in interventions due to their greater learning adaptability. This was shown as a side-effect in one of the studies in the review [36]. The analysed studies often fail to report on the total number of students in the cohort (e.g. academic year) from which the participants were recruited. Yet, the variability in sample size in studies indicates that this could be a common problem.

In clinical research the participation of the pharmaceutical industry in evaluation studies is to be judged as a source of bias. Yet, in medical education many of the developers of virtual patient systems and cases are among the authors of evaluation studies. This could bias how the interventions are evaluated, but this is rarely regarded as a conflict of interest by the community. Since this is common practice, the financial stakes in place are still relatively low and the relations between researchers and software developers are not sufficiently reported or possible for us to verify, we do not judge it as a separate risk. But in the future, when the production of virtual patients may turn more into a commercial product or service and not simply an outcome of academic development or research, this factor will need to be considered as well.

## Potential biases in the review process

A potential source of bias in the review was the decision made at the inception to exclude cross-over studies due to the risk of carry-over effects. We are aware of seven studies that were excluded from the review for this reason alone: [53–59]. To evaluate the risk of bias in the selection we analysed these excluded studies separately.

Four of the studies investigated differences between learning modalities: [54–56,58]. Three of them were based in North America, one in Australia [58]. In the study by Cobbett & Snelgrove-Clarke [54] no significant differences between a virtual patient intervention (VSim for Nursing) and mannequin-based scenario in nursing students' knowledge and confidence level were observed. Yet, the students felt more anxious after the experimental intervention than after mannequin-based training, and when directly compared their preferences, clearly favoured mannequin-based training. The two similar non-crossed studies comparing virtual patients to mannequins nursing were included in the review: [16,32] both had the same conclusion of no detectable changes in knowledge and skills. In addition, in one included study [60] noticed lower self-confidence after virtual patient intervention.

The second crossed-over study: Lyon et al. [55] showed no significant differences in knowledge outcomes in second year medical students between virtual patients (PlanAlyzer) and paper-cases, estimating that the former is more economical. A similar non-crossed study in radiology included in the review [17] also did not show differences, but stressed the practical benefits of not having the materials printed which saved resources retaining quality.

In the third study by McCoy et al. [56], a virtual patient intervention involving triads of first year osteopathic medicine students discussing a branched virtual patient case (DecisionSim/VpSim) was contrasted with small group (approx. 10 students) instructor-led discussion over PowerPoint cases displayed on a flat-screen. The traditional group turned out to perform better on an MCQ post-test. The result came as a surprise to the original investigator and was interpreted as potentially due to the item selection for the MCQ which relied too much on pattern recognition skills in ECG. Students in the study were used to practicing other types of skills with virtual patients, mainly related to inductive reasoning and not pattern recognition. While this might be so, in the face of the evidence we have collected in our systematic review the results are not that surprising and may be collated with the results of the study Al-Dahir et al. [41] who for the same virtual patient software compared individual pharmacy student of cardiac cases with PBL instructor led discussion groups showing superiority of the non e-Learning intervention. This example is aligned with the conclusion that comparison of two active learning methods will lead to small results. If in addition more individualised, human-based feedback is given than in a self-directed computer-based scenario, the guided group will perform better (as was shown explicitly in e.g. in the study Foster et al. [39] included in the review).

Finally, Tait et al. [58] investigated in a mixed-method study the differences in satisfaction level between three modalities: paper-based scenarios, virtual patients and standardised patient. Only 20 participants in total for three groups participated in the study. Results showed similar satisfaction level across all three study groups.

The three crossed studies investigating differences in virtual patient design were: [53,57,59]. Two of those, both located in United Kingdom, investigated the difference between linear and branched navigation in narrative patient scenarios [53,57]. This type of comparison was not present in the studies included in this review. The conclusions obtained from the crossed studies were contrary. Bateman [53] did not notice any differences in the outcomes when comparing branched and linear cases in the DecisionSim v2.0 software package, but estimated their design as more laborious than linear cases and hence less beneficial. Poulton et al [57] was able to show better outcomes in the branched virtual patients group (OpenLabyrinth system), but only in the questions related to the branching options. In addition to branching, Bateman was able to investigate a second factor in his study - the presence of structured clinical reasoning instruction (additional tasks to prompt reasoning) which was, in contrast to branching, found to positively influence the skills and satisfaction outcomes. Thompson et al [59] investigated the influence of case sequencing, explicit case comparison and writing case summaries using a crossed factorial design in the virtual patient system Medulator. Only writing case summaries had clearly positive effect on the skills outcomes in the study. No similar experiment was present in non-crossed studies included in this review.

The conclusion based on this analysis is that the exclusion of cross-over studies did not discard studies that substantially contradict findings from this review. As some of the studies had in addition factorial designs, compared the results of surveys item-by-item or did not present intermediate results before crossing, their inclusion was difficult in practical terms. Therefore, we judge that the exclusion of cross-over studies did not substantially bias our review.

Another type of bias could have been introduced while selecting the main comparison in studies with three or more arms. However, this was the case in one study only [26]. Li et al for comparison investigated a lecture-based group and two variants of PBL sessions: one paper-based and one ward-round-based. When selecting for contrast the PBL type which scored the best for the given samples on knowledge and skills tests (i.e. ward-round, real-patient based PBL), the differences to the virtual patient group were not statistically significant in post-hoc tests (without post-hoc correction the ward group was better in skills than virtual patient based). This is in line with our findings that active learning scenarios (e.g. different types of discussion groups contrasted with virtual patients) lead to small effect sizes or even preference for the control [41]. The attitudinal/satisfaction outcomes in the study were difficult to combine as compared item-by-item, yet there were two significant differences in items showing students were more confident in their skills when working with real than virtual patients. This aligns well with the previously discussed study [60] showing relatively less confidence by students using virtual patients. Our attempt to replace the selected effects of [26] in meta-analysis with the discussed alternatives, did not lead to lowering the level of heterogeneity to an acceptable level nor changed the general interpretation of the meta-analysis results. We conclude that our choice of study arms did not distort significantly the outcomes of the review.

## Overall completeness and applicability of evidence

Studies from North American represented the single largest geographical group with 19 out of 51 studies conducted in the United States of America. The country second in the number of reported studies was Germany with 5 studies. The use of virtual patients in low and middle-income countries was reported by 7 studies in total. This could limit the applicability of evidence to specific regions, but on the other hand our subgroup analyses did not show significant influence of income category and we report examples of positive outcomes of virtual patient interventions in low and middle income countries as well (e.g. [2,5,26,61]).

We found that 3^rd^ & 4^th^ year of study (which would be equivalent of 1^st^ & 2^nd^ year of medical school in North America or graduate-entry programmes) were the most studied population group. Twenty-six out of 51 studies reported results from this specific moment of education. This seems to be the natural place for locating virtual patients in the curriculum as it is the time when students begin to apply their core medical knowledge in practice. It could be hypothesised that for the earlier stages in the curriculum the use of virtual patients is too cognitively demanding, and that for the later stages there exist better, more realistic methods of education related to workplace-practice involving real human patients. Yet, in the early stages most of the few included studies showed positive effects [34,38,44]. The use of virtual patients in post-graduate education had positive reports [62,63], but there were studies showing no difference or negative results. In a large sample study in the review with a validated outcome measurement instrument that showed no difference after virtual patient intervention even when compared to no intervention [29]. In the study by Wang et al. [20] in a mixed group of health professionals (nurses, technologists and radiology residents) all but one items in the satisfaction survey were better rated for the mannequin-based then virtual patient group. The addition of virtual patients as a supplement to registered nurses complex skills training showed no significant difference when compared to the control group without access to virtual patients [24]. More research into the use of virtual patients in the early and late stages of the curriculum is needed and we encourage authors not to hesitate to report negative findings as this helps to develop a better understanding and improved placement of the virtual patient interventions in future. The publication bias in our review is difficult to investigate in a formal way due to high levels of heterogeneity which limit the interpretation possibilities of funnel plots.

# References

1. Berger J, Bawab N, De Mooij J, Sutter Widmer D, Szilas N, De Vriese C, et al. An open randomized controlled study comparing an online text-based scenario and a serious game by Belgian and Swiss pharmacy students. Curr Pharm Teach Learn 2018;10(3):267–276. PMID: 29764629

2. Botezatu M, Hult H, Tessma MK, Fors U. Virtual patient simulation: knowledge gain or knowledge loss? Med Teach 2010;32(7):562–8. PMID: 20653378

3. Trudeau KJ, Hildebrand C, Garg P, Chiauzzi E, Zacharoff KL. A Randomized Controlled Trial of the Effects of Online Pain Management Education on Primary Care Providers. Pain Med 2017 Jun 1;18(4):680–692. PMID: 28034967

4. Vash JH, Yunesian M, Shariati M, Keshvari A, Harirchi I. Virtual patients in undergraduate surgery education: a randomized controlled study. ANZ J Surg 2007;77(1–2):54–9. PMID: 17295822

5. Botezatu M, Hult H, Tessma MK, Fors UGH. Virtual patient simulation for learning and assessment: Superior results in comparison with regular course exams. Med Teach 2010 Apr;32(10):845–50. PMID: 20854161

6. Dankbaar MEW, Alsma J, Jansen EEH, van Merrienboer JJG, van Saase JLCM, Schuit SCE. An experimental study on the effects of a simulation game on students’ clinical cognitive skills and motivation. Adv Health Sci Educ Theory Pract 2016 Aug;21(3):505–21. PMID: 26433730

7. Davids MR, Chikte UME, Halperin ML. Effect of improving the usability of an e-learning resource: a randomized trial. Adv Physiol Educ 2014 Jun;38(2):155–60. PMID: 24913451

8. Kandasamy T, Fung K. Interactive Internet-based cases for undergraduate otolaryngology education. Otolaryngol Head Neck Surg 2009 Mar;140(3):398–402. PMID: 19248951

9. Smith BD, Silk K. Cultural competence clinic: an online, interactive, simulation for working effectively with Arab American Muslim patients. Acad Psychiatry 2011;35(5):312–6. PMID: 22007089

10. Succar T, Zebington G, Billson F, Byth K, Barrie S, McCluskey P, et al. The impact of the Virtual Ophthalmology Clinic on medical students’ learning: a randomised controlled trial. Eye (Lond) 2013 Oct;27(10):1151–7. PMID: 23867718

11. Tolsgaard MG, Jepsen RMHG, Rasmussen MB, Kayser L, Fors U, Laursen LC, et al. The effect of constructing versus solving virtual patient cases on transfer of learning: a randomized trial. Perspect Med Educ 2016 Feb;5(1):33–8. PMID: 26754313

12. Triola M, Feldman H, Kalet AL, Zabar S, Kachur EK, Gillespie C, et al. A randomized trial of teaching clinical skills using virtual and live standardized patients. J Gen Intern Med 2006 May;21(5):424–9. PMID: 16704382

13. Braun LT, Zottmann JM, Adolf C, Lottspeich C, Then C, Wirth S, et al. Representation scaffolds improve diagnostic efficiency in medical students. Med Educ 2017 Nov;51(11):1118–1126. PMID: 28585351

14. Deladisma AM, Gupta M, Kotranza A, Bittner JG, Imam T, Swinson D, et al. A pilot study to integrate an immersive virtual patient with a breast complaint and breast examination simulator into a surgery clerkship. Am J Surg 2009 Jan;197(1):102–6. PMID: 19101251

15. Weverling GJ, Stam J, ten Cate TJ, van Crevel H. [Computer-assisted education in problem-solving in neurology; a randomized educational study]. Ned Tijdschr Geneeskd 1996 Feb 24;140(8):440–3. PMID: 8720819

16. Liaw SY, Chan SW-C, Chen F-G, Hooi SC, Siau C. Comparison of virtual patient simulation with mannequin-based simulation for improving clinical performances in assessing and managing clinical deterioration: randomized controlled trial. J Med Internet Res 2014 Sep 17;16(9):e214. PMID: 25230684

17. Maleck M, Fischer MR, Kammer B, Zeiler C, Mangel E, Schenk F, et al. Do computers teach better? A media comparison study for case-based teaching in radiology. Radiographics 2001;21(4):1025–32. PMID: 11452078

18. Fleetwood J, Vaught W, Feldman D, Gracely E, Kassutto Z, Novack D. MedEthEx Online: a computer-based learning program in medical ethics and communication skills. Teach Learn Med 2000 Apr;12(2):96–104. PMID: 11228685

19. Mahnken AH, Baumann M, Meister M, Schmitt V, Fischer MR. Blended learning in radiology: is self-determined learning really more effective? Eur J Radiol 2011 Jun;78(3):384–7. PMID: 21288674

20. Wang CL, Chinnugounder S, Hippe DS, Zaidi S, O’Malley RB, Bhargava P, et al. Comparative Effectiveness of Hands-on Versus Computer Simulation-Based Training for Contrast Media Reactions and Teamwork Skills. J Am Coll Radiol Elsevier Inc; 2017 Jan;14(1):103-110.e3. PMID: 27815053

21. Lehmann R, Thiessen C, Frick B, Bosse HM, Nikendei C, Hoffmann GF, et al. Improving Pediatric Basic Life Support Performance Through Blended Learning With Web-Based Virtual Patients: Randomized Controlled Trial. J Med Internet Res 2015 Jul 2;17(7):e162. PMID: 26139388

22. Bearman M, Cesnik B, Liddell M. Random comparison of “virtual patient” models in the context of teaching clinical communication skills. Med Educ 2001 Sep;35(9):824–32. PMID: 11555219

23. Kinney P, Keskula DR, Perry JF. The effect of a computer assisted instructional program on physical therapy students. J Allied Health 1997;26(2):57–61. PMID: 9268782

24. Bryant R, Miller CL, Henderson D. Virtual Clinical Simulations in an Online Advanced Health Appraisal Course. Clin Simul Nurs 2015 Oct;11(10):437–444.

25. Kaltman S, Talisman N, Pennestri S, Syverson E, Arthur P, Vovides Y. Using Technology to Enhance Teaching of Patient-Centered Interviewing for Early Medical Students. Simul Healthc 2018 Jun;13(3):188–194. PMID: 29771814

26. Li J, Li QL, Li J, Chen ML, Xie HF, Li YP, et al. Comparison of three problem-based learning conditions (real patients, digital and paper) with lecture-based learning in a dermatology course: a prospective randomized study from China. Med Teach 2013;35(2):e963-70. PMID: 23009254

27. Miedzybrodzka Z, Hamilton NM, Gregory H, Milner B, Frade I, Sinclair T, et al. Teaching undergraduates about familial breast cancer: comparison of a computer assisted learning (CAL) package with a traditional tutorial approach. Eur J Hum Genet 2001 Dec;9(12):953–6. PMID: 11840198

28. Maier EM, Hege I, Muntau AC, Huber J, Fischer MR. What are effects of a spaced activation of virtual patients in a pediatric course? BMC Med Educ 2013 Mar 28;13(1):45. PMID: 23537162

29. Harris JM, Sun H. A randomized trial of two e-learning strategies for teaching substance abuse management skills to physicians. Acad Med 2013 Sep;88(9):1357–62. PMID: 23887001

30. Jeimy S, Wang JY, Richardson L. Evaluation of virtual patient cases for teaching diagnostic and management skills in internal medicine: A mixed methods study. BMC Res Notes 2018;11(1):1–7. PMID: 29871699

31. Kononowicz AA, Zary N, Edelbring S, Corral J, Hege I. Virtual patients--what are we talking about? A framework to classify the meanings of the term in healthcare education. BMC Med Educ 2015 Feb 1;15(1):11. PMID: 25638167

32. Secomb J, McKenna L, Smith C. The effectiveness of simulation activities on the cognitive abilities of undergraduate third-year nursing students: a randomised control trial. J Clin Nurs 2012 Dec;21(23–24):3475–84. PMID: 23145517

33. Leong SL, Baldwin CD, Adelman AM. Integrating Web-based computer cases into a required clerkship: development and evaluation. Acad Med 2003 Mar;78(3):295–301. PMID: 12634211

34. Schittek Janda M, Mattheos N, Nattestad A, Wagner A, Nebel D, Färbom C, et al. Simulation of patient encounters using a virtual patient in periodontology instruction of dental students: design, usability, and learning effect in history-taking skills. Eur J Dent Educ 2004 Aug;8(3):111–9. PMID: 15233775

35. Foster A, Chaudhary N, Murphy J, Lok B, Waller J, Buckley PF. The Use of Simulation to Teach Suicide Risk Assessment to Health Profession Trainees-Rationale, Methodology, and a Proof of Concept Demonstration with a Virtual Patient. Acad Psychiatry 2015 Dec;39(6):620–9. PMID: 25026950

36. Qayumi AK, Kurihara Y, Imai M, Pachev G, Seo H, Hoshino Y, et al. Comparison of computer-assisted instruction (CAI) versus traditional textbook methods for training in abdominal examination (Japanese experience). Med Educ 2004 Oct;38(10):1080–8. PMID: 15461653

37. Kumta SM, Tsang PL, Hung LK, Cheng JCY. Fostering critical thinking skills through a web-based tutorial programme for final year medical students--A randomized controlled study. J Educ Multimed Hypermedia 2003;12(3):267–273.

38. Bonnetain E, Boucheix J-M, Hamet M, Freysz M. Benefits of computer screen-based simulation in learning cardiac arrest procedures. Med Educ 2010 Jul;44(7):716–22. PMID: 20636591

39. Foster A, Chaudhary N, Kim T, Waller JL, Wong J, Borish M, et al. Using Virtual Patients to Teach Empathy: A Randomized Controlled Study to Enhance Medical Students’ Empathic Communication. Simul Healthc 2016 Jun;11(3):181–9. PMID: 26841278

40. Courteille O, Fahlstedt M, Ho J, Hedman L, Fors U, von Holst H, et al. Learning through a virtual patient vs. recorded lecture: a comparison of knowledge retention in a trauma case. Int J Med Educ 2018 Mar 28;9:86–92. PMID: 29599421

41. Al-Dahir S, Bryant K, Kennedy KB, Robinson DS. Online virtual-patient cases versus traditional problem-based learning in advanced pharmacy practice experiences. Am J Pharm Educ 2014 May 15;78(4):76. PMID: 24850938

42. Subramanian A, Timberlake M, Mittakanti H, Lara M, Brandt ML. Novel educational approach for medical students: improved retention rates using interactive medical software compared with traditional lecture-based format. J Surg Educ 2012;69(2):253–6. PMID: 22365876

43. Sobocan M, Turk N, Dinevski D, Hojs R, Pecovnik Balon B. Problem-based learning in internal medicine: virtual patients or paper-based problems? Intern Med J 2017 Jan;47(1):99–103. PMID: 27800653

44. Kononowicz AA, Krawczyk P, Cebula G, Dembkowska M, Drab E, Frączek B, et al. Effects of introducing a voluntary virtual patient module to a basic life support with an automated external defibrillator course: a randomised trial. BMC Med Educ 2012 Jun 18;12(1):41. PMID: 22709278

45. Higgins JPT, Green S. Cochrane handbook for systematic reviews of interventions. Chichester: John Wiley & Sons.; 2008.

46. Cook DA. Randomized controlled trials and meta-analysis in medical education: what role do they play? Med Teach 2012;34(6):468–73. PMID: 22489980

47. Lahti M, Hätönen H, Välimäki M. Impact of e-learning on nurses’ and student nurses knowledge, skills, and satisfaction: a systematic review and meta-analysis. Int J Nurs Stud 2014 Jan;51(1):136–49. PMID: 23384695

48. Rasmussen K, Belisario JM, Wark PA, Molina JA, Loong SL, Cotic Z, et al. Offline eLearning for undergraduates in health professions: A systematic review of the impact on knowledge, skills, attitudes and satisfaction. J Glob Health 2014 Jun;4(1):010405. PMID: 24976964

49. Friedman CP. The research we should be doing. Acad Med 1994 Jun;69(6):455–7. PMID: 8003158

50. Cook DA. The research we still are not doing: an agenda for the study of computer-based learning. Acad Med 2005 Jun;80(6):541–8. PMID: 15917356

51. Clark RC. Scenario-based e-learning: Evidence-based guidelines for online workforce learning. San Francisco: John Wiley & Sons.; 2013.

52. Callahan CA, Hojat M, Gonnella JS. Volunteer bias in medical education research: an empirical study of over three decades of longitudinal data. Med Educ 2007 Aug;41(8):746–53. PMID: 17661882

53. Bateman J. Virtual patient design in undergraduate education. PhD [dissertation]. University of Warwick; 2013.

54. Cobbett S, Snelgrove-Clarke E. Virtual versus face-to-face clinical simulation in relation to student knowledge, anxiety, and self-confidence in maternal-newborn nursing: A randomized controlled trial. Nurse Educ Today 2016 Oct;45:179–84. PMID: 27537670

55. Lyon HC, Healy JC, Bell JR, O’Donnell JF, Shultz EK, Wigton RS, et al. Findings from an evaluation of PlanAlyzer’s double cross-over trials of computer-based, self-paced, case-based programs in anemia and chest pain diagnosis. Proceedings Symp Comput Appl Med Care 1991;88–93. PMID: 1807738

56. McCoy L. Virtual Patient Simulations for Medical Education: Increasing Clinical Reasoning Skills through Deliberate Practice. PhD [dissertation]. Arizona State University; 2014.

57. Poulton T, Ellaway RH, Round J, Jivram T, Kavia S, Hilton S. Exploring the efficacy of replacing linear paper-based patient cases in problem-based learning with dynamic Web-based virtual patients: randomized controlled trial. J Med Internet Res 2014 Nov 5;16(11):e240. PMID: 25373314

58. Tait L, Lee K, Rasiah R, Cooper JM, Ling T, Geelan B, et al. Simulation and Feedback in Health Education: A Mixed Methods Study Comparing Three Simulation Modalities. Pharm (Basel, Switzerland) 2018 May 3;6(2):41. PMID: 29751528

59. Thompson GA, Morrison RG, Holyoak KJ, Clark TK. Evaluation of an Online Analogical Patient Simulation Program. 19th IEEE Symp Comput Med Syst 2006. p. 623–628.

60. Williams C, Aubin S, Harkin P, Cottrell D. A randomized, controlled, single-blind trial of teaching provided by a computer-based multimedia package versus lecture. Med Educ 2001 Sep;35(9):847–54. PMID: 11555222

61. Gu Y, Zou Z, Chen X. The Effects of vSIM for Nursing^TM^ as a Teaching Strategy on Fundamentals of Nursing Education in Undergraduates. Clin Simul Nurs 2017 Apr;13(4):194–197.

62. Schwid HA, Rooke GA, Ross BK, Sivarajan M. Use of a computerized advanced cardiac life support simulator improves retention of advanced cardiac life support guidelines better than a textbook review. Crit Care Med 1999 Apr;27(4):821–4. PMID: 10321676

63. Schwid HA, Rooke GA, Michalowski P, Ross BK. Screen-based anesthesia simulation with debriefing improves performance in a mannequin-based anesthesia simulator. Teach Learn Med 2001;13(2):92–6. PMID: 11302037
