# Supplementary material for: Virtual Patient Simulations in Health Professions Education: Systematic Review and Meta-Analysis by the Digital Health Education Collaboration
Source: J Med Internet Res. 2019 Jul 2;21(7):e14676. doi: 10.2196/14676 (PMC6632099; doi:10.2196/14676)
Supplement: Multimedia Appendix 8 [file jmir_v21i7e14676_app8.doc]

# Multimedia Appendix 8: Summary of findings tables

**Table 1. Summary of finding table: effects of virtual patient versus traditional education** on knowledge, skills, attitudes and satisfaction

| **Virtual patient versus traditional education** | | | | | |
| --- | --- | --- | --- | --- | --- |
| **Patient or population**: pre- or post-registration health professionals **Settings** pre-registration and post-registration healthcare professional education **Intervention**: virtual patient simulation  **Comparison**: traditional education | | | | | |
| **Outcomes** | **Illustrative comparative risks* (95% CI)** | **Relative effect (95% CI)** | **No of participants (studies)** | **Quality of the evidence (GRADE)** | **Comments** |
| **Knowledge**  Measured by MCQ (n=12), mix of paper-based question formats (n=3), preference survey (n=1), unclear (n=2) | The mean score in the intervention group was **0.11** standard deviations **higher** (0.17 lower to 0.39 higher) than in the control group. | Not estimable | 1213 participants  (18 studies; 4 excluded from the meta-analysis) | ⊕⊕⊝⊝ **Low**^a,b^ | An SMD of 0.11 indicates virtual patients are as effective as traditional education. |
| **Skills**  Measured by paper-based production tests (n=5), virtual patients and paper-based cases (n=2), on mannequins (n=5) and standardised patients (n=2) | The mean score in the intervention group was **0.90 higher** (0.49 higher to 1.32 higher) than in the control group. | Not estimable | 1041 participants  (14 studies;  2 excluded from the meta-analysis) | ⊕⊕⊝⊝ **Low**^a,b^ | An SMD of 0.90 indicates a large effect size favouring virtual patients. |
| **Attitudes**  Measured by self-assessment questionnaires (n=5) | Not estimable | Not estimable | 475 participants  (5 studies) | ⊕⊝⊝⊝ **Very Low** ^b,c,d^ |  |
| **Satisfaction**  Measured by satisfaction survey (n=10) | Not estimable | Not estimable | 640 participants  (10 studies) | ⊕⊝⊝⊝ **Very Low** ^b,c,d^ |  |
| *The **corresponding risk** (and its 95% confidence interval) is based on the assumed risk in the comparison group and the **relative effect** of the intervention (and its 95% CI).  **CI**: confidence interval; **SMD**: Standardised mean difference. | | | | | |
| GRADE Working Group grades of evidence **High quality**: further research is very unlikely to change our confidence in the estimate of effect. **Moderate quality**: further research is likely to have an important impact on our confidence in the estimate of effect and may change the estimate. **Low quality**: further research is very likely to have an important impact on our confidence in the estimate of effect and is likely to change the estimate. **Very low quality**: we are very uncertain about the estimate. | | | | | |

**Footnotes**

^a^Rated down by one level because of high heterogeneity of included studies;
^b^Rated down by one level because of limitations in study design (lack of participant blinding, non-validated instruments, volunteer bias);
^c^Rated down by one level as most of the studies present the outcomes as independent items in questionnaires that were not amenable to statistical analysis;

**Table 2 Summary of findings table virtual patient blended learning versus traditional education**

| **Virtual patient blended learning versus traditional education** | | | | | |
| --- | --- | --- | --- | --- | --- |
| **Patient or population**: pre- or post-registration health professionals **Settings** pre-registration and post-registration healthcare professional education **Intervention**: virtual patient simulation blended with traditional education  **Comparison**: traditional education | | | | | |
| **Outcomes** | **Illustrative comparative risks* (95% CI)** | **Relative effect (95% CI)** | **No of participants (studies)** | **Quality of the evidence (GRADE)** | **Comments** |
| **Knowledge**  Measured by MCQ (n=4), mix of paper-based question formats (n=1) | The mean score in the intervention group was **0.73 higher** (0.24 higher to 1.22 higher) than in the control group. | Not estimable | 439 participants  (5 studies) | ⊕⊝⊝⊝ **Very Low** ^a,b,c^ | An SMD of 0.73 indicates a moderate effect size favouring virtual patients blended with traditional education. |
| **Skills**  Measured by mannequins (n=3), standardised patients (n=2), real patients (n=1) and production tests (n=1) | The mean score in the intervention group was **0.60 higher**  (0.07 lower to 1.27 higher) than in the control group. | Not estimable | 554 participants  (7 studies; 3 excluded from the meta-analysis) | ⊕⊝⊝⊝ **Very Low** ^a,b,c^ | An SMD of 0.60 indicates a moderate effect size favouring virtual patients . |
| **Attitudes**  Measured by self-assessment questionnaires (n=4) | Not estimable | Not estimable | 324 participants  (4 studies) | ⊕⊝⊝⊝ **Very Low** ^a,b,d^ |  |
| **Satisfaction**  - | Not estimable | Not estimable | -  (0 studies) | n/a | No study compared satisfaction outcomes in studies were virtual patient was blended with virtual patients to traditional education alone. |
| *The **corresponding risk** (and its 95% confidence interval) is based on the assumed risk in the comparison group and the **relative effect** of the intervention (and its 95% CI).  **CI**: confidence interval; **SMD**: Standardised mean difference. | | | | | |
| GRADE Working Group grades of evidence **High quality**: further research is very unlikely to change our confidence in the estimate of effect. **Moderate quality**: further research is likely to have an important impact on our confidence in the estimate of effect and may change the estimate. **Low quality**: further research is very likely to have an important impact on our confidence in the estimate of effect and is likely to change the estimate. **Very low quality**: we are very uncertain about the estimate. | | | | | |

**Footnotes**

^a^Rated down by one level because of high heterogeneity of included studies;
^b^Rated down by one level because of limitations in study design (lack of participant blinding, non-validated instruments, volunteer bias);
^c^Rated down by one level because of small number of studies
^d^Rated down by one as most of the studies present the outcomes as independent items in questionnaires that were not amenable to statistical analysis and the number of studies were small;

**Table 3. Summary of findings table virtual patient versus other digital health education**

| **Virtual patient versus other digital health education** | | | | | |
| --- | --- | --- | --- | --- | --- |
| **Patient or population**: pre- or post-registration health professionals **Settings** pre-registration and post-registration healthcare professional education **Intervention**: virtual patient simulation  **Comparison**: other (non-virtual patient) digital health education | | | | | |
| **Outcomes** | **Illustrative comparative risks* (95% CI)** | **Relative effect (95% CI)** | **No of participants (studies)** | **Quality of the evidence (GRADE)** | **Comments^a^** |
| **Knowledge**  Measured by MCQ (n=2) | Not estimable | Not estimable | 345 participants  (2 studies) | ⊕⊝⊝⊝ **Very Low** ^a,b,c^ |  |
| **Skills**  Measured by mannequins (n=1), standardised patients (n=1) and mix of MCQ, OSCE and real patient examination (n=1) | Not estimable | Not estimable | 204 participants  (3 studies) | ⊕⊝⊝⊝ **Very Low** ^a,b,c^ |  |
| **Attitudes**  Measured by satisfaction (n=1) | Not estimable | Not estimable | 205 participants  (1 studies) | ⊕⊝⊝⊝ **Very Low** ^a,b,c^ |  |
| **Satisfaction**  Measured by satisfaction survey (n=2) | Not estimable | Not estimable | 108 participants  (2 studies) | ⊕⊝⊝⊝ **Very Low** ^a,b,c^ |  |
| *The **corresponding risk** (and its 95% confidence interval) is based on the assumed risk in the comparison group and the **relative effect** of the intervention (and its 95% CI).  **CI**: confidence interval; **SMD**: Standardised mean difference. | | | | | |
| GRADE Working Group grades of evidence **High quality**: further research is very unlikely to change our confidence in the estimate of effect. **Moderate quality**: further research is likely to have an important impact on our confidence in the estimate of effect and may change the estimate. **Low quality**: further research is very likely to have an important impact on our confidence in the estimate of effect and is likely to change the estimate. **Very low quality**: we are very uncertain about the estimate. | | | | | |

**Footnotes**

^a^Rated down by one level because of high heterogeneity of included studies;
^b^Rated down by one level because of limitations in study design (lack of participant blinding, non-validated instruments, volunteer bias);
^c^Rated down by one level as most of the studies present the outcomes as independent items in questionnaires that were not amenable to statistical analysis;

**Table 4**. Summary of findings table virtual patient versus other virtual patient design

| **Virtual patient versus other virtual patient design** | | | | | |
| --- | --- | --- | --- | --- | --- |
| **Patient or population**: pre- or post-registration health professionals **Settings** pre-registration and post-registration healthcare professional education **Intervention**: virtual patient  **Comparison**: other virtual patient design | | | | | |
| **Outcomes** | **Illustrative comparative risks* (95% CI)** | **Relative effect (95% CI)** | **No of participants (studies)** | **Quality of the evidence (GRADE)** | **Comments** |
| **Knowledge**  Measured by MCQ (n=2), mix of paper-based question formats (n=4), unclear (n=1) | Not estimable | Not estimable | 594 participants  (7 studies) | ⊕⊝⊝⊝ **Very Low** ^a,b,c^ |  |
| **Skills**  Measured by standardised patients (n=3), virtual patients (n=1_ | Not estimable | Not estimable | 258 participants  (4 studies) | ⊕⊝⊝⊝ **Very Low** ^a,b,c^ |  |
| **Attitudes**  Measured by survey (n=1) | Not estimable | Not estimable | 70 participants  (1 studies) | ⊕⊝⊝⊝ **Very Low** ^a,b,c^ |  |
| **Satisfaction**  Measured by satisfaction survey (n=4) | Not estimable | Not estimable | 213 participants  (4 studies) | ⊕⊝⊝⊝ **Very Low** ^a,b,c^ |  |
| *The **corresponding risk** (and its 95% confidence interval) is based on the assumed risk in the comparison group and the **relative effect** of the intervention (and its 95% CI).  **CI**: confidence interval; **SMD**: Standardised mean difference. | | | | | |
| GRADE Working Group grades of evidence **High quality**: further research is very unlikely to change our confidence in the estimate of effect. **Moderate quality**: further research is likely to have an important impact on our confidence in the estimate of effect and may change the estimate. **Low quality**: further research is very likely to have an important impact on our confidence in the estimate of effect and is likely to change the estimate. **Very low quality**: we are very uncertain about the estimate. | | | | | |

**Footnotes**

^a^Rated down by one level because of high heterogeneity of included studies;
^b^Rated down by one level because of limitations in study design (lack of participant blinding, non-validated instruments, volunteer bias);
^c^Rated down by one level as most of the studies present the outcomes as independent items in questionnaires that were not amenable to statistical analysis;
